# Ab-initio and density functional theory (DFT) computational study of the effect of fluorine on the electronic, optical, thermodynamic, hole and electron transport properties of the circumanthracene molecule

**DOI:** 10.1016/j.heliyon.2023.e19647

**Published:** 2023-09-03

**Authors:** L. Fomekong Tsague, G.W. Ejuh, A. Teyou Ngoupo, Y. Tadjouteu Assatse, R.A. Yossa Kamsi, M.T. Ottou Abe, J.M.B. Ndjaka

**Affiliations:** a University of Yaoundé I, Department of Physics, P.O. Box 812, Yaoundé, Cameroon; b University of Bamenda, National Higher Polytechnic Institute, Department of Electrical and Electronic Engineering, P. O. Box 39, Bambili, Cameroon; c University of Dschang, IUT-FV Bandjoun, Department of General and Scientific Studies, P.O. Box 134, Bandjoun, Cameroon

**Keywords:** Doped circumanthracene, Optoelectronic, Photonic, Organic solar cell, Internal reorganization energy

## Abstract

In this paper, a systematic study of the electronic, optical, thermodynamic, optoelectronic, and nonlinear optical properties with RHF, B3LYP, wB97XD and BPBE methods using the cc-pVDZ basis set have been described to investigate the influence of fluorine (F) atom, which is an electron donor, on the circumanthracene (C_40_H_16_). Global reactivity descriptors, hole and electron transport properties were also calculated and compared with other studies on the same molecule. DFT/B3LYP results show that the undoped C_40_H_16_ molecule (Egap = 2.135 eV) and its fluorine-doped derivatives (C_40_F_16_ and C_40_H_10_F_6_) are semiconducting materials. However, doping the C_40_H_16_ molecule with the fluorine atom, partially or totally, favors the creation of a strong donor-acceptor system by considerably reducing its energy gap (Egap). The energy gap values of molecules doped using DFT/B3LYP method are 2.020 eV and 2.081 eV for the C_40_F_16_ and C_40_H_10_F_6_ molecules, respectively. These gap energies are below 3 eV, which favours the electronic properties of these molecules. They can be used to design organic solar cells. The nonlinear optical parameters were calculated and compared with those of urea. The values of βmol and μ calculated for C_40_F_16_ and C_40_H_10_F_6_ are higher than those of urea; this shows that these two materials have good nonlinear optical properties and therefore, are very good candidates for the design of optoelectronics and photonics devices. Futhermore, our results show that the perfluorination effect on the circumanthracene molecule increases the hole and electron reorganization energies, the vertical and adiabatic electron affinities and ionization energies, the optoelectronic and nonlinear optical properties, the transition excitation energy and the reactivity indices. The reorganization energies values suggest that these materials have promising transport properties. The natural bond orbital (NBO) analysis was also performed to determine the stability energy and charge delocalization in molecules. The theoretical results of the compounds studied in our work are in agreement with the experimental results. This confirms their molecular structures.

## Highlights

• RHF, B3LYP, wB97XD and BPBE methods using the cc-pVDZ basis set have used to investigate the influence of fluorine on the circumanthracene.
• Global reactivity descriptors, hole and electron transport properties were also calculated and compared.
• Materials have good nonlinear optical properties and are very good candidates for the design of optoelectronics and photonics devices.
• The perfluorination effect on circumanthracene molecule increases the hole and electron reorganization energies and nonlinear optical properties.
• The values of the reorganization energies suggest that these materials have promising transport properties.

## 1 Introduction

In recent years, polycyclic aromatic hydrocarbons (PAHs) have received much attention in the field of optoelectronics. These aromatic hydrocarbons are small organic molecules that, in the crystalline state, are widely used as active elements in various optoelectronic devices such as thin-film organic field effect transistors, photovoltaic cells, light-emitting diodes and liquid crystals [[1], [2], [3], [4]]. The particularity of these small molecules compared to polymers is that chemical modification or addition of functional groups to the conjugated ring can easily increase their electronic, optoelectronic, optical and nonlinear optical properties [[5], [6], [7]]. In addition, chemical modification with strongly electronegative substituents is an effective approach to convert a p-type semiconductor to an n-type one [8,9]. Indeed, n-type materials based on PAHs are generally obtained by fixing strong electron-withdrawing groups such as the cyano group (CN) attached to the conjugated ring, or by peripheral substitution of hydrogen atoms by halogen atoms (fluorine (F), chlorine (Cl)) [[10], [11], [12]]. Attaching electron-withdrawing substituents such as CN, F and Cl to the π-conjugated ring or functionalizing the conjugated rings lowers the lowest molecular orbital energy level and the electron gap, and provides very high electron affinities, facilitating electrons injection. It has been shown by Pinheiro et al. that the controlled introduction of substitution defects is an effective way to tailor the electronic, magnetic and physicochemical properties of graphene conjugated materials [10,13]. Among PAH-based molecules, the role of circumacenes (coronene, circumanthracene, circumtetracene, ovalene and circumpentacene) has been recently highlighted due to their promising properties for organic and molecular electronics [14,15]. This family of planar and symmetric molecules could play an essential role in optoelectronic devices, as they are generally considered to be finite parts of graphene, their infinite counterpart [15,16]. Some preliminary results on optical and UV–Vis properties have shown that these molecules have promising properties in photovoltaic applications. Several studies have been conducted by different research groups on the calculation of electron affinity, ionization energy, optical absorption spectrum, and exciton binding energies of some perfluorinated polycyclic aromatic hydrocarbons (PAHs) [17,18]. The first ever studies on the electronic properties and systematic calculation on perfluorinated circumanthracenes was performed by Cardia et al. [19]. For this study, we used the fluorine atom as a dopant because it has a high electronegativity (3.98) and favors the injection of electrons from the valence band (HOMO energy) to the conduction band (LUMO energy). Thus, doping an organic compound with fluorine, like other electron attractors, makes their electronic properties interesting. The fluorine atom has a Vander Walls radius (R_vdw_ = 1.47 Å) equivalent to that of the hydrogen atom (R_vdw_ = 1.20 Å); consequently, substitution of the latter by fluorine will not alter the steric effect of the molecule [20]. Thus, the C–F bond, being polar and stronger than a C–H bond, confers great chemical stability on the molecule. Several scientists in the field of medicine have shown that one-fifth (1/5) of marketed drugs contain one or more fluorine atoms, mainly in the form of a C–F bond instead of a C–H bond [21]. Our motivation for the study of this molecule comes from the fact that it has already been synthesized among the circumacenes family and because it is very much studied due to its importance applications in electronic and optoelectronic devices. In this paper we will focus on the circumanthracene molecule and its perfluorinated derivatives, where all hydrogen atoms are replaced by fluorine atoms and then six hydrogen atoms are replaced by six fluorine atoms, to study their effects on the structural, electronic, hole and electron transport properties, which are calculated as a function of geometries and molecular boundary orbitals. Therefore, we decided to perform ab initio, density functional theory (DFT) and time-dependent density functional theory (TD-DFT) calculations to quantify the effect of the complete and partial substitution of peripheral hydrogen atoms by fluorine atoms on the different molecular properties. For each of the molecules considered, we will study the geometric structures, the electronic properties, the thermodynamic properties at different temperatures, the reorganization energies of holes and electrons, the optoelectronic and nonlinear optical properties, and absorption spectra compared to experimental values. We will focus on the work of other researchers to perform not only a systematic calculation of the optical and electronic properties, but also to study the structural properties, thermodynamic properties, optoelectronic properties, and nonlinear optics of circumanthracene (C_40_H_16_), circumanthracene-F (C_40_F_16_) and C_40_H_10_F_6_ molecule using the RHF, B3LYP, wB97XD, and BPBE methods with the cc-PVDZ basis set. In addition, we will also perform a self-consistent field calculations on the neutral and charged (±1) systems to evaluate: adiabatic and vertical electron affinities, adiabatic and vertical ionization energies, corrected quasiparticle energy band gap, transition excitation energy, exciton binding energy, molecular reorganization energies of holes and electrons.

## 2 Theoretical methodology

The molecular structures and geometrical parameters of circumanthracene (C_40_H_16_), circumanthracene-F (C_40_F_16_), and (C_40_H_10_F_6_) molecules were optimized using quantum mechanical calculation with the density functional theory DFT (B3LYP, wB97XD and BPBE), Hartree- Fock (HF), and time-dependent density function (TD-DFT) methods using the cc-pVDZ basis set which is the consistent basis set with Dunning correlation. The B3LYP method was used by S. Selvaraj et al. [22], for UV–Vis spectrum calculation, vibrational spectrum analysis, NMR analysis, electronic properties and NBO analysis. All calculations were implemented with the Gaussian 09W chemical quantum program [23] and visualized using the Gauss View, Rev 5.0.8 [24] molecular visualization program. Initially, the optimized geometries of our compounds were performed using the ab-initio HF method with the smaller 3-21G basis set. Subsequently, the obtained molecular structure results were used with the cc-pVDZ basis set [25]. Calculations of our compounds were firstly performed using the RHF method. The molecular structures were confirmed using the DFT/B3LYP method which is a primordial method that takes into account the inclusion of electronic correlations. Then, to confirm the molecular geometric structure of these molecules, we performed vibrational frequency analysis using the PED method and no imaginary frequencies were observed; this implies the stability of these molecules and also indicates that the structure of the molecule corresponds to at least a local minimum on the potential energy surface. Vibrational mode assignments based on the potential energy distribution were performed using the Vibrational Energy Distribution Analysis (VEDA) program [26]. The VEDA program depends on the local coordinates entered; it automatically reads the input data from the frequency output files of the Gaussian 09 program. Theoretical frequencies calculated with Gaussian software are generally associated with systematic errors due to insufficient consideration of electronic correlation effects and neglect of harmonicity [27]. To overcome these errors, scaled frequencies close to the experimental values are obtained from the unscaled frequencies by multiplying the theoretical values by an appropriate scaling factor. The calculated vibrational frequencies were evaluated using the scaling factors 0.908, 0.966, and 0.953 for the RHF/cc-pVDZ, B3LYP/cc-pVDZ and wB97XD/cc-pVDZ theory levels [28].

Studies in the ultraviolet–visible (UV–Vis), such as absorption wavelength, excitation energy, oscillator strengths and major contributions of all these molecules, were performed using time-dependent density functional theory (TD-DFT) with the cc-pVDZ basis set and compared with experimental studies of circumanthracene absorption spectra [10]. Electronic properties such as HOMO-LUMO energies, quasiparticle corrected HOMO-LUMO gap, excitation bond energy, Kohn-Sham HOMO-LUMO energy were calculated at RHF, B3LYP, wB97XD, and BPBE levels with the same basis set. The global reactivity descriptors and nonlinear optical properties have also been calculated. Thermodynamic properties at different temperatures were studied using the same methods and basis sets.

**Fig. 1:**
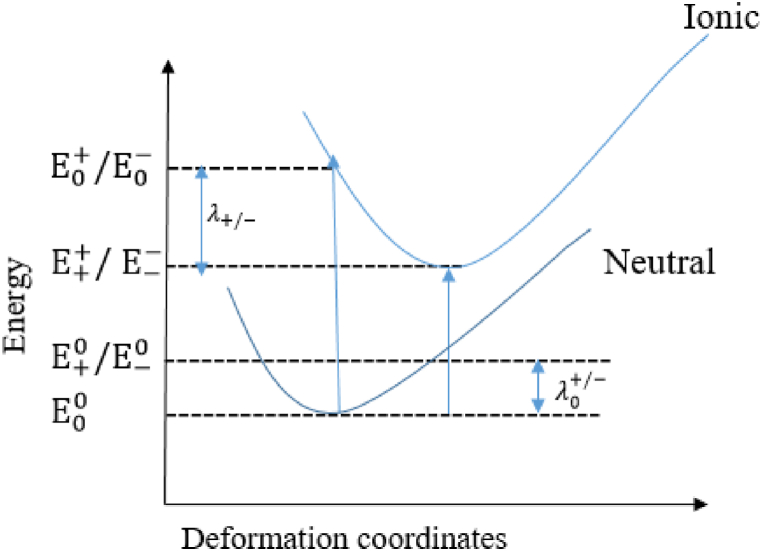
Schematic representation for the calculation of the internal reorganization energy (λ). The curves represent the potential energies of the neutral and cationic species.

The hole and electron reorganization energies of our compounds were calculated using the RHF, B3LYP, wB97XD, and BPBE methods with the cc-pVDZ basis. These methods and basis sets allowed us to optimize the geometries in both neutral and ionic states. The reorganization energies of electrons and holes were evaluated using the adiabatic potential energy surface method according to the Marcus model [29]. The Schematic representation for the calculation of the internal reorganization energy (λ) is presented in Fig. 1. Thus, the values of λ are calculated according to Equations (1), (2)).

(1) $$\lambda_{hole}=\lambda_{0}+\lambda_{+}=\left(E_{0}^{+}-E_{+}^{+}\right)+\left(E_{+}^{0}-E_{0}^{0}\right)$$

(2) $$\lambda_{electron}=\lambda_{0}+\lambda_{-}=\left(E_{0}^{-}-E_{-}^{-}\right)+\left(E_{-}^{0}-E_{0}^{0}\right)$$

where $E_{+}^{+}$/ $E_{-}^{-}$ and $E_{0}^{0}$ represent the energy of the cation/anion form and the neutral species in the optimized geometries, respectively; while $E_{+}^{0}$/ $E_{-}^{0}$ and $E_{0}^{+}$/ $E_{0}^{-}$ denote the energy of the cation/anion in the neutral form, and the energy of the neutral molecule in the cationic/anionic geometry, respectively.

Non-linearity on a microscopic scale can be defined by the induced dipole moment of materials active in non-linear optics. Thus, the induced dipole moment is written as a series development of the field (F) (Equation (3)).

(3) $$\mu_{i}\left(F\right)=\mu_{0}+\sum_{j}\alpha_{ij}F_{j}+\sum_{j,k}\beta_{ijk}F_{j}F_{k}+\cdot \cdot \cdot$$

where $\mu_{O}$ is the permanent dipole moment of the molecule in the absence of an electric field, $\alpha_{ij}$ and $\beta_{ijk}$ are the components of the polarizability tensor and are the components of the first static hyperpolarizability tensor.

Within the limits of weak polarization introduced by relatively weak electric fields, the energy E of a system (Equation (4)) can also be written as a Taylor series expansion induced by the finite field (FF) [[30], [31], [32]].

(4) $$E=E^{\left(0\right)}-\sum_{i}\mu_{i}F_{i}-\frac{1}{2}\sum_{i,j}\alpha_{ij}F_{i}F_{j}-\frac{1}{6}\sum_{i,j,k}\beta_{ijk}F_{i}F_{j}F_{k}-\frac{1}{24}\sum_{i,j,k,l}\gamma_{ijkl}F_{i}F_{j}F_{k}F_{l}-...$$

where E ^(0)^ is the total energy of the molecular system in the absence of an electric field; $\mu_{i}$, $\alpha_{ij}$, $\beta_{ijk}$ and $\gamma_{ijkl}$ are the components of the dipole moment, linear polarizability, first hyperpolarizability and second hyperpolarizability tensors, respectively. The i, j and k components of the tensor label the x, y and z axes respectively. From the above equations, the values of x, y, z and z can be obtained by differentiating the energy E with respect to F. The dipole moment μ (Equation (5)), mean polarizability α_0_ (Equation (6)), static first hyperpolarizability β (Equation (8)) and second hyperpolarizability γ (Equation (9)) are determined from the components calculated with the Gaussian program using the following formulas.

The total dipole moment of a molecule is obtained using the equation:

(5) $$\mu ={\left(\mu_{x}^{2}+\mu_{y}^{2}+\mu_{z}^{2}\right)}^{\frac{1}{2}}$$

The average polarizability, which represents the trace of the polarizability matrix, is defined by:

(6) $$\alpha_{0}=\frac{1}{3}\left(\alpha_{xx}+\alpha_{yy}+\alpha_{zz}\right)$$

The anisotropic polarizability is given by Equation (7).

(7) $$\Delta \alpha =\frac{1}{2}\sqrt{{\left(\alpha_{xx}-\alpha_{yy}\right)}^{2}+{\left(\alpha_{yy}-\alpha_{zz}\right)}^{2}+{\left(\alpha_{zz}-\alpha_{xx}\right)}^{2}+6\left(\alpha_{xx}^{2}+\alpha_{yy}^{2}+\alpha_{zz}^{2}\right)}$$

First hyper-polarizability (β) (Equation (8)) is a third order tensor that can be described by a 3 × 3 × 3 matrix.

(8) $$\beta ={\left[{\left(\beta_{xxx}+\beta_{xyy}+\beta_{xzz}\right)}^{2}+{\left(\beta_{yyy}+\beta_{yxx}+\beta_{yzz}\right)}^{2}+{\left(\beta_{zzz}+\beta_{zxx}+\beta_{zyy}\right)}^{2}\right]}^{\frac{1}{2}}$$

In the non-linear domain, second hyperpolarizability is given by:

(9) $$\gamma =\frac{1}{5}\left(\gamma_{xxxx}+\gamma_{yyyy}+\gamma_{zzzz}+2\left(\gamma_{xxyy}+\gamma_{xxzz}+\gamma_{yyzzz}\right)\right)$$

## 3 Results and discussion

### 3.1 Molecular structure and geometric properties

**Fig. 2:**
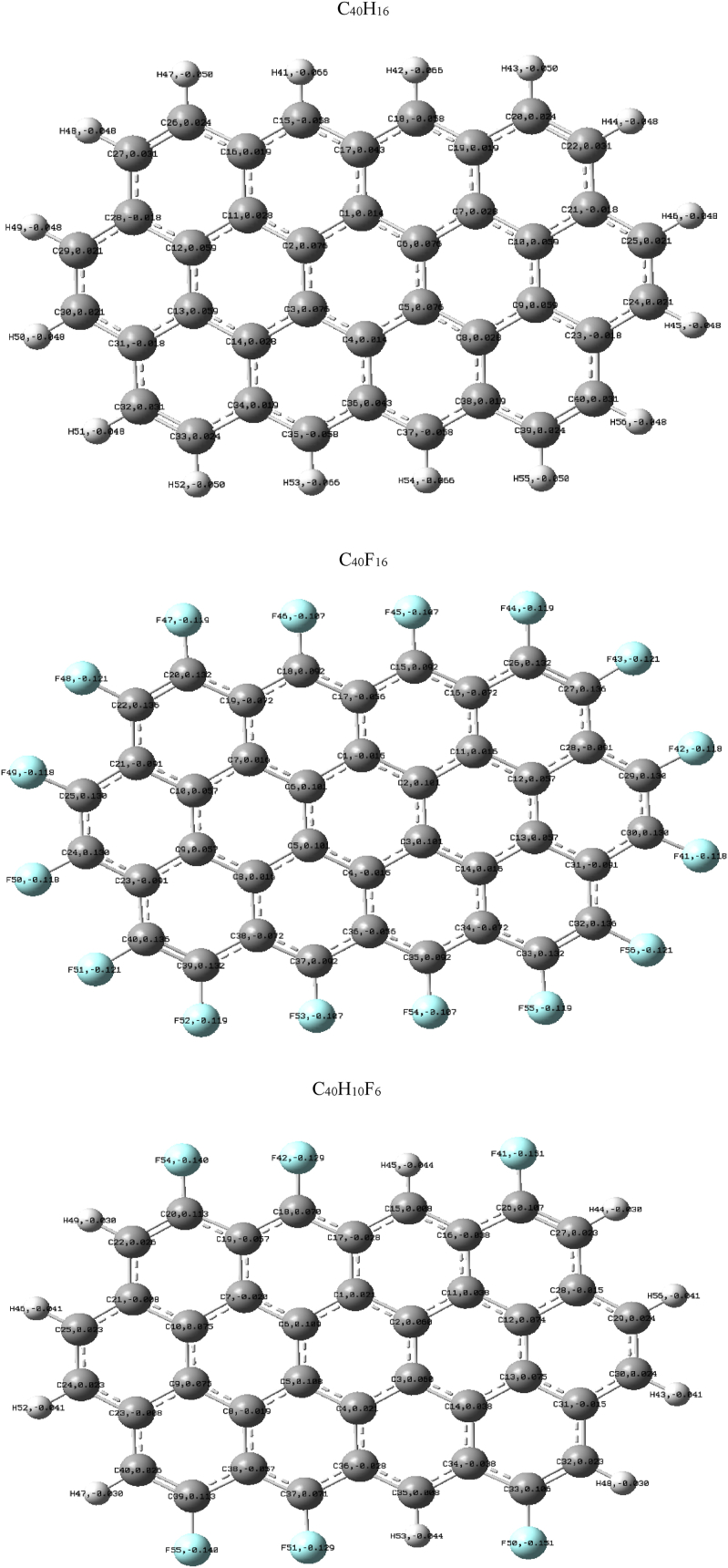
Optimized geometric structure of circumanthracene (C_40_H_16_), circumanthracene-F (C_40_F_16_), and C_40_H_10_F_6_ molecules obtained using B3LYP/cc-pVDZ.

The optimized geometrical parameters (bond lengths and valence angles) of the C_40_H_16_, C_40_F_16_, and C_40_H_10_F_6_ molecules obtained, using the RHF, B3LYP, wB97XD, and BPBE functionals with the cc-pVDZ basis set are given in Tables S1, S2, and S3 of the supplementary material document. These parameters are numbered according to the molecular structure given in Fig. 2. We found a slight difference between the geometric parameters calculated with the B3LYP, wB97XD, and BPBE functionals. Furthermore, the wB97XD functional differs significantly from the B3LYP and BPBE functions due to the short- and long-range interactions it takes into account during geometric optimization. Thus, the addition of dispersion effects influences the structural parameters of a molecule. We equally observed that doping the molecules with fluorine atoms slightly increases the structural parameters of these molecules; this observation is well pronounced for the C_40_F_16_ molecule compared to that of C_40_H_10_F_6_. This may be due to the fact that fluorine is more electronegative than hydrogen and carbon atoms; also, the C–F bond length is very polar. It is known in the literature that the RHF exchange functional underestimates the geometrical parameters of a molecule, while the exchange and correlation functions (B3LYP and BPBE) increase these parameters to allow a better agreement between theory and experiment [33,34]. This observation is made in our work because most of the geometric parameters obtained with B3LYP, wB97XD, and BPBE functionals are similar to experimental and theoretical values reported in literature [[35], [36], [37]]. The optimized molecular structures of C_40_H_16_, C_40_F_16_, and C_40_H_10_F_6_ molecules are presented in Fig. 2 using the B3LYP/cc-pVDZ method. This method is used because it allowed us to obtain the lowest value of the potential energy and to validate our results. We observed according to the Mulliken atomic charge analysis (Fig. 2) of these compounds that all hydrogen and fluorine atoms are negatively charged. The net charge on these atoms ranges from −0.66e to 0.76e, −0.121e to 0.131e, and −0.151e to 0.113e respectively for C_40_H_16_, C_40_F_16_, and C_40_H_10_F_6_ molecules using the B3LYP/cc-pVDZ method. We noticed that some carbon atoms were having positive charges, while others had negative charges. This implies that, in these molecules, some carbon atoms act as electron donors and others as electron acceptors. The representation of the C–C, C = C, and C–F bond lengths, C–C–C, C–C–H, and C–C–F valence angles obtained using the RHF, B3LYP, wB97XD, and BPBE functionals with the cc-pVDZ basis set are given in Fig. S1 (Supplementary Material). It is observed that the C–C, C = C, C–H, and C–F bond lengths of all these molecules vary in the following order: RHF ˂ wB97XD ˂ B3LYP ˂ BPBE. It is also found that the bond lengths increase slightly with doping and vary in the following order: C_40_H_16_< C_40_H_10_F_6_ < C_40_F_16_. For doped molecules (Fig. S1), we note that the maximum C–C distance lengths are obtained with the BPBE functional and the minimum lengths with the RHF functional. Thus, for the C_40_F_16_ molecule, the maximum and minimum C–C bond lengths are 1.445 Å and 1.395 Å respectively. In contrast, the maximum length obtained with the BPBE functional is 1.45 Å and the minimum length obtained with the RHF functional is 1.395 Å for the C_40_H_10_F_6_ molecule. For the undoped molecule, we find that the C–C bond length is maximal (1.45 Å) and minimal (1.395 Å) only with the RHF functional. These observations show that doping improves molecule bond lengths and enhances electronic and thermodynamic properties. We also observe thatC = C bond lengths, for the molecules C_40_H_16_ and C_40_F_16_, evolve in a similar way. This shows that the C–F bond, which is more polar than the C–H bond, does not modify the structure of the molecule, but rather improves its properties. We note that the C–C–C bond angles of undoped and doped molecules increase slightly in a similar fashion, ranging from 117° to 125°. Thus, the C–C–C bond angle varies from 118° to 122°, from 118° to 123° and from 117° to 124° respectively for C_40_H_16_, C_40_F_16_ and C_40_H_10_F_6_ molecules.

### 3.2 Hole and electron reorganization energies

The development of new device technologies based on solar cells requires linking the charge transport properties to the molecular structure of the conjugate material from which they are derived. Thus, the reorganization energy of a molecular system in electrochemistry, solid state physics, and biology allows us to understand the relationship between the charge transfer properties and the structural properties of a molecule. The reorganization energy of electrons and holes is a key parameter in determining the mobility of charge carriers in the donor and acceptor molecule, respectively. The molecular reorganization energies λ_h_ and λ_e_ of the holes and electrons, respectively, are reported in Table 1. The values of holes reorganization energy (λ_h_) increase from 0.044 eV (for the C_40_H_10_F_6_ molecule) to 0.089 eV (for the C_40_F_16_ molecule) at the B3LYP/cc-pVDZ level, while that of the electrons (λ_e_) increases from 0.027 eV to 0.074 eV at the same level. In the field of nanoelectronics description, these increases suggest that the relaxation energy of the intramolecular geometry is smaller for some molecules with large spatial delocalization in the HOMO orbital. Thus, several groups of researchers have shown that this observation is true for π-planar systems such as PAHs as reported in the literature [38,39]. In Table 1, we observed that the value of the holes reorganization energy for the C_40_H_16_ molecule obtained with the B3LYP/cc-pVDZ method is similar to that obtained by Malloci et al. [40] and by Sancho et al. [41]. We also observed that, regardless of the calculation method used, the reorganization energies λ_h_ and λ_e_ increase significantly with the total substitution of hydrogen atoms by fluorine (F) atoms in the C_40_H_16_ molecule. On the other hand, these energies decrease when we go from the C_40_F_16_ molecule to the C_40_H_10_F_6_ molecule, which means that the substitution of some hydrogen atoms by fluorine (F) atoms decreases the reorganization energies of the electrons no matter what level of calculation used. These observations may be due to the fact that the addition of an electron-donating atom or moiety increases the bond angles and bond lengths between adjacent rings which are generally sensitive to the change in the number of electrons in π-planar systems. We also observe that the reorganization energy of holes λ_h_ is lower than that of electrons at all levels of theory. The increase in electrons and holes reorganization energies for the C_40_F_16_ molecule is due to the fact that fluorine is more electronegative than hydrogen. Table 1 shows that the C_40_H_16_ molecule has the smallest hole reorganization energy at all levels of theory compared to the doping molecule (C_40_F_16_ and C_40_H_10_F_6_). In the literature, it has been shown that the molecular reorganization energy critically affects the charge transfer process, this means that a low reorganization energy leads to a high transfer rate (high charge mobility) [42]. Thus, we can say that C_40_H_16_ is a good material for charge transfer and therefore, has a very high charge mobility. These results are in agreement with those of Bredas et al. [38] who showed that fluorine substitution in PAHs compounds increases both λ_h_ and λ_e_ reorganization energies, resulting in a degradation of transport properties of the molecules.

**Table 1:** Reorganization energy of holes (λ_h_) and electrons (λ_e_) in eV for C_40_H_16_, C_40_F_16_, and C_40_H_10_F_6_ for the RHF, B3LYP, wB97XD, and BPBE methods.

	C_40_H_16_				C_40_F_16_				C_40_H_10_F_6_			
	RHF	B3LYP	wB97XD	BPBE	RHF	B3LYP	wB97XD	BPBE	RHF	B3LYP	wB97XD	BPBE
$\lambda_{1}$	0.283	0.037	0.079	0.021	0.357	0.081	0.128	0.058	0.307	0.061	0.102	0.040
$\lambda_{2}$	0.291	0.036	0.079	0.021	0.367	0.081	0.127	0.058	0.314	0.056	0.102	0.040
$\lambda_{h/eV}$	0.575	0.073	0.091	0.042	0.723	0.162	0.256	0.117	0.622	0.117	0.204	0.080
$\lambda_{1}^{\prime }$	0.293	0.046	0.028	0.028	0.386	0.083	0.137	0.061	0.322	0.063	0.109	0.041
$\lambda_{2}^{\prime }$	0.301	0.046	0.091	0.029	0.403	0.083	0.137	0.061	0.338	0.567	0.110	0.041
$\lambda_{e/eV}$	0.594	0.092	0.182	0.057	0.789	0.166	0.275	0.122	0.660	0.119	0.219	0.082

### 3.3 Optical properties: UV–vis analysis

When the fluorine atom is substituted by the hydrogen atom on the circumanthracene molecule (C_40_H_16_), it has some influence on its morphological and molecular structure. The aim of this part of our study is therefore to observe the effect of fluorine on the electronic transitions of the circumanthracene molecule. To find this effect, the electronic spectra of the circumanthracene molecule is calculated at the same theory levels. In Table 2, we list the values of absorption wavelengths (*λ*_*max*_), oscillation strengths (*f*), energies of excitations, and some major contributions using the ab-initio and time-dependent DFT methods (TD-RHF, TD-B3LYP, TD-wB97XD, and TD-BPBE) with the cc-pVDZ basis set and RHF, B3LYP, wB97XD, and BPBE geometries. We performed a calculation of the first twenty excited states and then listed the transitions with an oscillation strength greater than 0.01. From Table 2, the following results can be found.

➢ The nature of the transition is different from the circumanthracene molecule (C_40_H_16_) and the doping molecules (C_40_F_16_ and C_40_H_10_F_6_). For the C_40_H_16_ molecule, the main contribution is $\pi \to \pi^{*}$. For the isomers of this molecule, the maximum absorption is due to the charge transfer from the fluorine atom to the circumanthracene molecule. Thus, the other electronic transitions can be attributed to metal ligand charge transfer (MLCT) or ligand metal charge transfer (LMCT). However, we find that MLCT transitions are much more numerous than LMCT transitions. Therefore, when the fluorine atom on the circumanthracene molecule is substituted, the nature of the transition obviously changes;
➢ The possible transitions (*f* > 0.01) of these isomers are more numerous than that of the individual molecule, especially for the C_40_H_10_F_6_ molecule using any functional group. This also proves that a strong donor-acceptor interaction leads to more abundant transitions.

**Table 2:** Theoretical electronic absorption spectra of C_40_H_16_, C_40_F_16_, and C_40_H_10_F_6_ molecules (absorption wavelength λ (nm), excitation energy E (eV), oscillator strength (*f*), and major contribution obtained using the time-dependent methods (TD-RHF, TD-B3LYP, TD-wB97XD, and TD-BPBE) with the cc-pVDZ basis set.

Molecules	Methods	*E (eV)*	*λ*_*max*_ (nm)	*f*			Major contributions
**C**_**40**_**H**_**16**_	**TD-RHF**	**2.810**	**441.20**	**0.3608**			**H- > L (85%), H-1- > L+1 (7%)**
		3.494	354.83	0.0212			H-1- > L (47%), H- > L+1 (43%)
		4.767	260.09	3.3847			H-1- > L (46%), H- > L+1 (49%)
		5.217	237.66	0.7071			H-1- > L+1 (54%), H- > L (11%)
		5.663	218.96	0.7521			H-2- > L+3 (28%), H-3- > L+2 (11%)
		5.935	208.91	0.1593			H-8- > L (16%), H-5- > L+1 (16%)
		6.179	200.67	0.2103			H-3- > L+3 (14%), H-2- > L+2 (22%)
	**TD-B3LYP**				**B3LYP/6**–**31+G(d)** [14]		
					***E (eV)***	***f***	
		**2.027**	**611.72**	**0.1784**	**3.25**	**1.324**	**H- > L (98%)**
		3.352	369.88	1.2542	**4.15**	**0.404**	H-1- > L (43%), H - > L+2 (48%)
		4.146	299.03	0.3783	**2.03**	**0.186**	H-2- > L+1 (43%), H-1- > L+2 (43%)
		**2.027**	**611.72**	**0.1784**	**4.46**	**0.095**	H-3- > L+1 (32%), H-2- > L+3 (65%)
					**3.20**	**1.159**	
					**4.25**	**0.691**	
					**3.95**	**0.391**	
					**1.94**	**0.191**	
	**TD-wB97XD**	**2.449**	**506.19**	**0.2803**			**H- > L (96%)**
		4.008	309.30	2.1787			H-1- > L (46%), H- > L+1 (49%)
		4.145	299.09	0.0266			H-4- > L (22%), H-2- > L+2 (26%)
		4.930	251.49	0.3455			H-2- > L+3 (30%), H-1- > L+5 (14%)
		4.678	265.04	0.3626			H-1- > L+1 (79%), H-6- > L+6 (3%)
		5.083	243.93	0.2393			H-8- > L (33%), H-4- > L+1 (15%)
	**TD-BPBE**	**1.762**	**703.84**	**0.1209**			**H- > L (97%), H-1- > L+1 (3%)**
		2.912	425.83	0.7603			H-2- > L (39%), H- > L+2 (44%)
		3.318	373.72	0.0197			H-2- > L+2 (22%), H-1- > L+1 (67%)
		3.523	351.96	0.0155			H-3- > L+1 (41%), H-1- > L+3 (58%)
		3.575	346.83	0.2272			H-2- > L+2 (65%), H-1- > L+1 (19%)
		3.688	336.13	0.0641			H-8- > L (83%), H- > L+8 (12%)
**C**_**40**_**F**_**16**_	**TD-RHF**	**2.873**	**431.51**	**0.3560**			**H- > L (84%), H-1- > L+1 (7%)**
		3.411	363.45	0.1449			H-1- > L (24%), H- > L+1 (67%)
		4.834	256.49	3.3222			H-1- > L (66%), H- > L+1 (28%)
		5.633	220.11	0.4303			H-3- > L+2 (14%), H-2- > L+3 (30%)
		6.032	205.54	0.2840			H-7- > L (16%), H-5- > L+1 (22%)
		6.179	200.67	0.1847			H-2- > L+2 (21%), H- > L+5 (21%)
		5.180	239.32	0.7758			H-1- > L+1 (52%), H- > L+5 (14%)
	**TD-B3LYP**	**1.929**	**642.64**	**0.1762**			**H- > L (98%)**
		2.427	510.87	0.0474			H-2- > L (33%), H- > L+1 (66%)
		3.186	389.10	1.0768			H-2- > L (62%), H- > L+1 (30%)
		3.669	337.85	0.0158			H-2- > L+1 (52%), H-1- > L+2 (28%)
		3.916	316.58	0.3510			H-2- > L+1 (37%), H-1- > L+2 (47%)
		4.038	307.01	0.0106			H-7- > L (91%), H-3- > L+2 (4%)
	**TD-wB97XD**	**2.367**	**523.66**	**0.2850**			**H- > L (96%)**
		2.806	441.84	0.0931			H-2- > L (27%), H- > L+1 (67%)
		3.919	316.32	2.0654			H-2- > L (66%), H - > L+1 (29%)
		4.475	277.04	0.3592			H-2- > L+1 (78%), H- > L+5 (9%)
		4.688	264.43	0.3208			H-7- > L (16%), H-3- > L+2 (15%)
	**TD-BPBE**	**1.628**	**761.50**	**0.1128**			**H- > L (97%)**
		2.081	595.79	0.0207			H-2- > L (40%), H- > L+1 (59%)
		2.634	470.61	0.5617			H-2- > L (50%), H - > L+1 (31%)
		3.253	381.09	0.2123			H-2- > L+1 (59%), H-1- > L+2 (25%)
		3.354	369.70	0.0717			H-6- > L (91%), H- > L+8 (4%)
**C**_**40**_**H**_**10**_**F**_**6**_	**TD-RHF**	**2.835**	**437.30**	**0.3488**			**H- > L (84%), H-1- > L+1 (7%)**
		3.468	357.53	0.1027			**H-1- > L (29%), H- > L+1 (62%)**
		4.761	260.44	1.2247			H-2- > L+1 (23%), H-1- > L (25%)
		4.853	255.50	1.9453			H-1- > L (37%), H- > L+1 (18%)
		5.237	236.74	0.609			H-4- > L (14%), H-1- > L+1 (43%)
		5.332	232.54	0.1071			H-2- > L (14%), H-1- > L+3 (17%)
		5.597	221.52	0.7932			H-2- > L+3 (39%), H- > L+8 (22%)
		5.802	213.69	0.0772			H-4- > L (25%), H-2- > L+4 (26%)
		5.977	207.43	0.1534			H-4- > L+1 (26%), H-2- > L+6 (14%)
		6.221	199.30	0.0241			H-2- > L+2 (13%), H- > L+5 (24%)
		6.262	197.967	0.0187			H-5- > L+1 (17%), H-3- > L+1 (17%)
	**TD-B3LYP**	**1.965**	**630.84**	**0.1708**			**H- > L (98%)**
		2.544	487.36	0.0453			H-2- > L (34%), H- > L+1 (65%)
		3.319	373.52	0.9446			H-2- > L (51%), H- > L+1 (26%)
		3.359	369.05	0.1495			H-3- > L (77%), H-2- > L (7%)
		3.524	351.85	0.0326			H-5- > L (16%), H-1- > L+1 (68%)
		3.674	337.41	0.0195			H-1- > L+2 (25%), H- > L+5 (67%)
		3.987	310.97	0.1998			H-3- > L+2 (12%), H-1- > L+3 (80%)
		4.0218	308.28	0.0353			H-6- > L (20%), H- > L+7 (48%)
		4.096	302.65	0.2728			H-2- > L+1 (47%), H-1- > L+2 (22%)
	**TD-wB97XD**	**2.405**	**515.52**	**0.2768**			**H- > L (96%)**
		2.925	423.92	0.0681			H-2- > L (31%), H - > L+1 (63%)
		3.969	312.33	1.3236			H-2- > L (42%), H- > L+1 (22%)
		4.074	304.34	0.6146			H-2- > L (17%), H-1- > L+1 (28%)
		4.115	301.29	0.0314			H-4- > L (40%), H-1- > L+2 (23%)
		4.162	297.91	0.0737			H-3- > L (68%), H-2- > L+2 (7%)
		4.686	264.55	0.3282			H-2- > L+1 (71%), H-5- > L+6 (2%)
		4.775	259.66	0.0299			H- > L+7 (50%) H-8- > L (7%)
		4.7856	259.08	0.4718			H-1- > L+3 (55%), H- > L+8 (18%)
		4.971	249.39	0.0126			H-8- > L (71%), H- > L+7 (11%)
		5.0031	247.8	0.0188			H-5- > L (16%), H-4- > L+1 (17%)
		5.011	247.44	0.0241			H-4- > L+1 (12%), H-1- > L+1 (25%)
		5.138	241.29	0.0689			H-1- > L+2 (33%), H-1- > L+4 (24%)
	**TD-BPBE**	**1.663**	**745.33**	**0.1074**			**H - > L (95%), H-1- > L+2 (2%)**
		2.228	556.47	0.0376			H-2- > L (34%), H - > L+1 (66%)
		2.828	438.27	0.0310			H-3- > L (72%), H - > L+3 (12%)
		2.853	434.56	0.5534			H-2- > L (50%), H- > L+1 (23%)
		2.988	414.87	0.0295			H-1- > L+1 (82%), H-2- > L+2 (4%)
		3.149	393.72	0.0234			H-1- > L+2 (37%), H- > L+5 (46%)
		3.194	388.14	0.0302			H-5- > L (55%), H - > L+6 (40%)
		3.216	385.50	0.0310			H-1- > L+2 (45%), H- > L+5 (41%)
		3.3298	372.34	0.1470			H-3- > L+2 (17%), H-1- > L+3 (62%)
		3.366	368.27	0.0466			H-5- > L (14%), H-2- > L+2 (51%)
		3.4725	357.04	0.0440			H-7- > L (89%), H - > L+8 (5%)
		3.5339	350.84	0.1287			H-2- > L+1 (61%), H-3- > L+1 (5%)
		3.5527	348.99	0.0142			H-3- > L+1 (41%), H-2- > L+3 (48%)

This study shows that the maximum wavelength (*λ*_max_) is strongly related to the lowest energy gap of the frontier molecular orbital (FMO). This study shows that the maximum wavelengths and oscillation strengths of the compounds evolve in a similar way for the TD-DFT methods. We also find from our study that the maximum wavelengths are inversely proportional to the excitation energies of each molecule for each of the time dependent methods. Thus, in our study, the maximum wavelengths all correspond to the minimum excitation energies. We know that the band gap (Eg) of a molecule decreases when it is doped with a more electronegative atom, it then becomes possible for an electron to make a transition from the ground state to the excited state, so it needs less excitation energy E and absorbs a longer wavelength than the undoped molecule. This proves that doped molecules (C_40_F_16_ and C_40_H_10_F_6_) will readily undergo absorption to an excited state compared to the undoped molecule. The transition energy E of the C_40_H_16_ molecule decreases from the C_40_F_16_ molecule to the C_40_H_10_F_6_ molecule for the TD-DFT methods, and decreases from 1.965 eV (C_40_H_10_F_6_ molecule) to 1.929 eV (C_40_F_16_ molecule) for the TD-B3LYP method. This is also observed for the TD-wB97XD and TD-BPBE methods. From these three time-dependent DFT methods, it can be seen that when all fluorine (F) atoms replaced by hydrogen (H) atoms, the maximum wavelength increases while the oscillation strengths decrease slightly. It is also found that the wavelengths increase when moving from the C_40_H_16_ molecule to the C_40_F_16_ molecule, and then decrease slightly for the C_40_H_10_F_6_ molecule using TD-DFT methods with the cc-pVDZ basis set. It is observed that the wavelength is maximum on the C_40_F_16_ molecule and minimum on the C_40_H_16_ molecule, using TD-DFT methods.

**Fig. 3:**
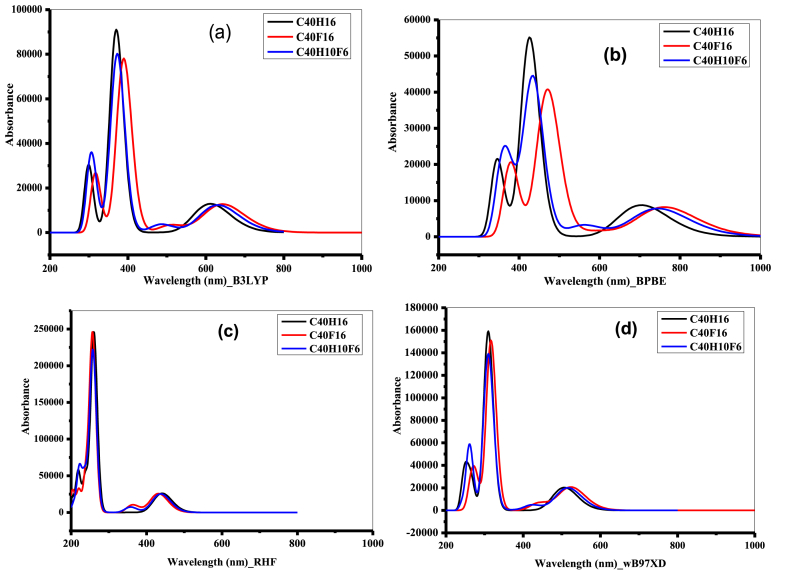
UV–vis absorption spectra for the C_40_H_16_, C_40_F_16_ and C_40_H_10_F_6_ molecules obtained with TD-B3LYP (a), TD-BPBE (b), TD-RHF (c), and TD-wB97XD (d) methods by employing cc-pVDZ basis sets.

We can conclude that since fluorine is a much more electronegative halogen than hydrogen, the number of electrons would have a significant effect on the wavelength of the molecules. This finding is inversely proportional to the oscillation strengths of these same compounds using TD-DFT methods. We also find that the maximum wavelengths are inversely proportional to the oscillation strengths. Thus, as the wavelengths increase, the oscillation forces decrease. The experimental absorption wavelength maxima of 609 nm, 582 nm, 540 nm, 502 nm, 470 nm, 380 nm and 364 nm of the circumanthracene molecule, in 1, 2, 4-tri-chlorobenzene obtained in the work of Richard et al. [43] Clar et al. [44] agree well with the values calculated theoretically for the compound C_40_H_16_ in the gas phase. Roberto et al. [19] carried out a study on the optical properties of circumanthracene and circumanthracene-F (C_40_F_16_) using the TD-DFT/B3LYP method with the 6–31+ G* basis set. By comparing the excitation energies and oscillator strengths obtained for these two basis sets, we can see that both values vary slightly, which proves that the two basis sets (6-31+G* and cc-pVDZ) do not have a major effect on the optical properties. Thus, we find in this study that the absorption with maximum oscillator strength, when moving from the reference molecule to the fluorine (F)-substituted molecules, is due to the charge transfer from the hydrogen atoms to the fluorine atoms in the C_40_H_16_ molecule; and that the calculated wavelengths of these compounds are in the ultraviolet (UV) and visible range. Fig. 3 shows the UV–Vis absorption spectra of the wavelengths of each molecule according to the methods used. In Fig. 3, it can be seen that the absorbance of the undoped molecule is higher compared to the doped molecules using the B3LYP (Fig. 3a) and BPBE (Fig. 3b) functionals. The maximum absorbances for the BPBE functional all match in the visible range. On the other hand, for the B3LYP (Fig. 3a), RHF (Fig. 3c), and wB97XD (Fig. 3d) functionals, the maximum absorbances are all in the ultraviolet (UV) range. We also note that doping significantly decreases the transmittances of the molecules. This peak shift observed with the TD-BPBE and TD-B3LYP methods is due to the electronic correlation effects taken into account by the B3LYP and BPBE functionals.

### 3.4 Thermodynamics properties

**Fig. 4:**
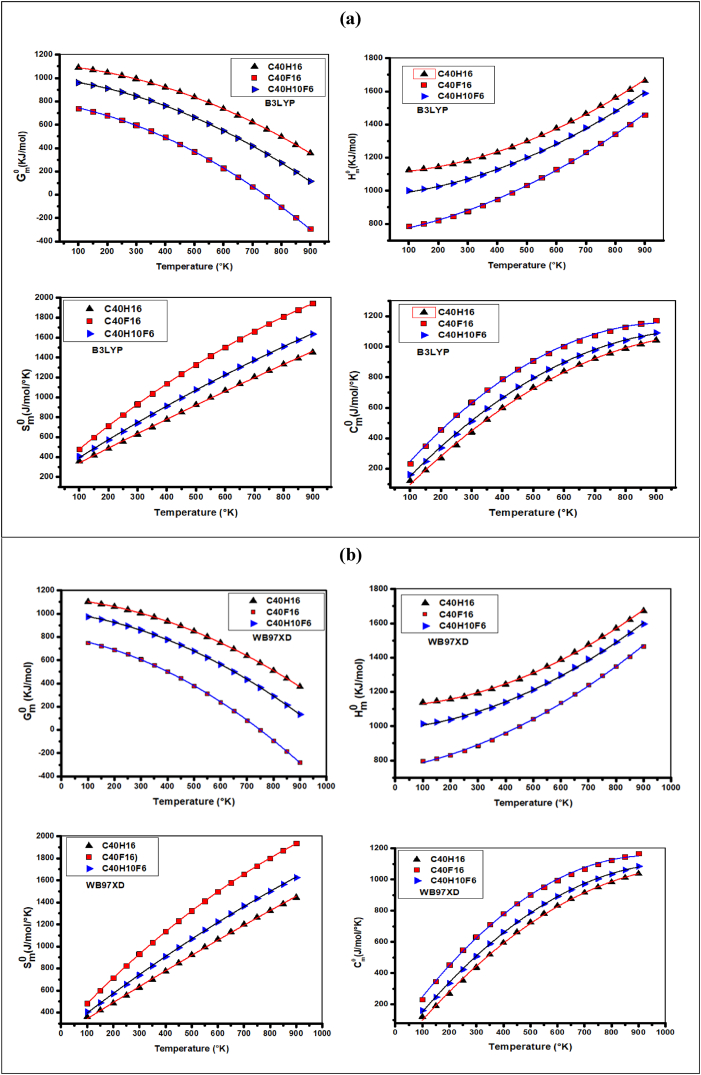

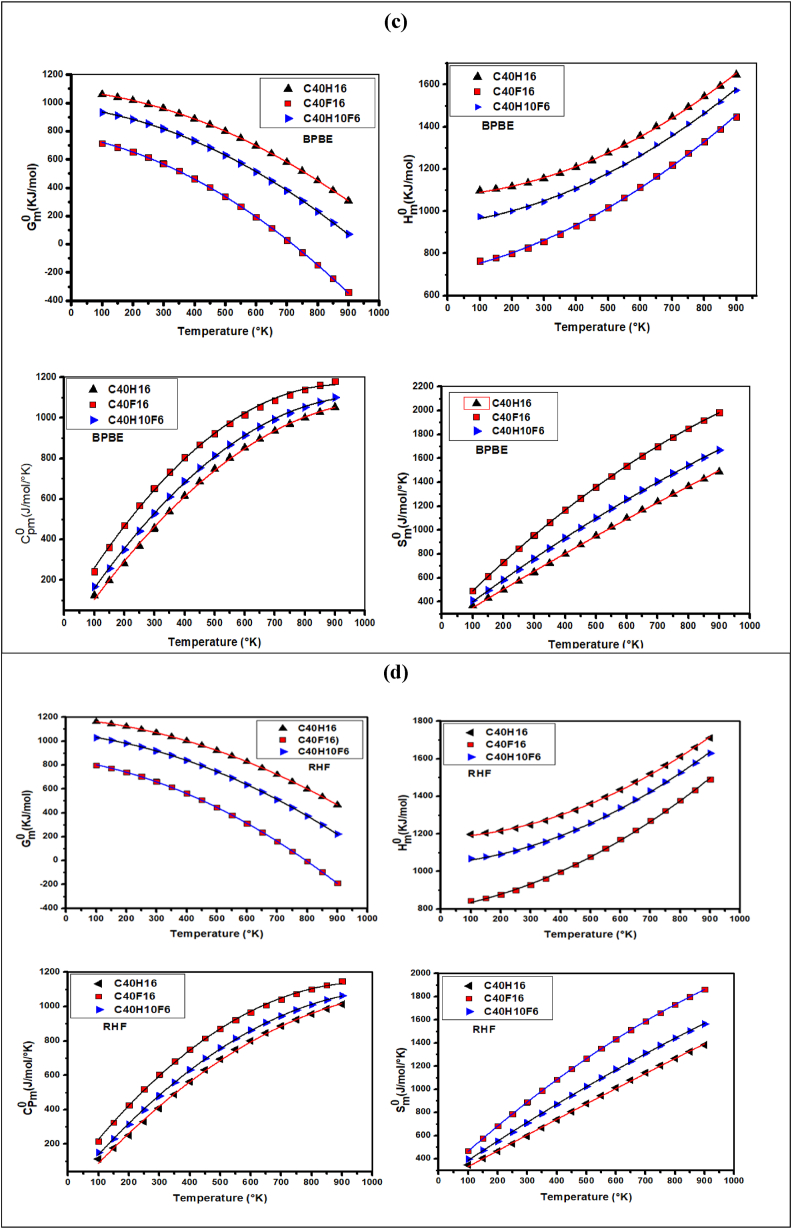
Correlation graphs of thermodynamic properties (entropy, enthalpy, Gibbs energy, and heat capacity) at different temperature and constant pressure of the molecules C_40_H_16_, C_40_F_16_, and C_40_H_10_F_6_ according to different methods: (a) B3LYP, (b) wB97XD, (c) BPBE, (d) RHF.

The values of some thermodynamic properties of the C_40_H_16_, C_40_F_16_, and C_40_H_10_F_6_ molecules, using the RHF, B3LYP, wB97XD, and BPBE methods at a temperature of 298. 15K and the pressure of 1 atm are listed in Table 3. Among these thermodynamic properties, some standard thermodynamic functions (Gibbs free energy ${(G}_{m}^{0})$, enthalpy $\left(H_{m}^{0}\right)$, entropy ${(S}_{m}^{0})$, and constant pressure heat capacity ${(C}_{p,m}^{0})$) of these molecules have been calculated from vibrational analysis and statistical thermodynamics at the previous methods, for temperatures ranging from 100K to 900K. These thermodynamic properties can be presented in equation form and the correlation plots between the thermodynamic properties $G_{m}^{0}$, $H_{m}^{0}$, $C_{p,m}^{0}$, and $S_{m}^{0}$ and temperatures are shown in Fig. 4. It appears from this study that the Gibbs free energy $\left(G_{m}^{0}\right)$ and enthalpy ${(H}_{m}^{0})$ decrease significantly from the reference molecule (C_40_H_16_) through the C_40_F_16_ molecule to the C_40_H_10_F_6_ molecule for all methods used (B3LYP (Fig. 4a), wB97XD (Fig. 4b), BPBE (Fig. 4c), and RHF (Fig. 4d)) with temperatures ranging from 100K to 900K. It is found that for temperatures above 750K, the Gibbs free energy values are negative for the C_40_F_16_ molecule. It is also found that the heat capacity at constant pressure $\left(C_{p,m}^{0}\right)$ and entropy $\left(S_{m}^{0}\right)$ increase significantly when moving from the C_40_H_16_ molecule through the C_40_H_10_F_6_ molecule for all methods used (B3LYP (Fig. 4a), wB97XD (Fig. 4b), BPBE (Fig. 4c), and RHF (Fig. 4d)) in this study. The maximum values of ${(C}_{p,m}^{0})$ and $\left(S_{m}^{0}\right)$ are only obtained on the C_40_F_16_ molecule, and minimum on the C_40_H_16_ molecule. The values of $G_{m}^{0}$ and $H_{m}^{0}$ are maximum on the C_40_H_16_ molecule, and minimum on the C_40_F_16_ molecule. The values of $G_{m}^{0}$ and $H_{m}^{0}$ are maximum on the C_40_H_16_ molecule and minimum for the C_40_F_16_ molecule. Therefore, the carbon fluorine (C–F) covalent bonding of C_40_F_16_ and C_40_H_10_F_6_ compounds significantly decreases the standard thermodynamic properties ($G_{m}^{0}$ and $H_{m}^{0}$). On the other hand, this same carbon-fluorine (C–F) bond significantly increases the values of $C_{p,m}^{0}$ and $S_{m}^{0}$. The quadratic correlation equations of the molecular structures modeled with the B3LYP, wB97XD, BPBE, and RHF computational levels are given in Table 4.

**Table 3:** Electronic energy (EE), Zero point vibration energy (ZPVE), Thermal energy (E_T_), Thermal correction to Energy (U), Thermal correction to Enthalpy (H_Th_), Thermal correction to Gibbs Free Energy (G_Th_), Sum of electronic and zero-point Energies (E1), Sum of electronic and thermal Energies (E2), Sum of electronic and thermal Enthalpies (H), Sum of electronic and thermal Gibbs Free Energies (G), Heat capacity at constant pression (C_P_), Heat capacity at constant volume (C_V_), Translational energy $\left(E_{Trans}\right)$, Rotational energy $\left(E_{Rot}\right)$, Vibrational energy $\left(E_{Vib}\right)$ of the molecules C_40_H_16_, C_40_F_16_, and C_40_H_10_F_6_ using RHF, B3LYP, wB97XD and BPBE methods by employing the cc-pVDZ basis set. All energies whose units are not mentioned are in Hartree.

	C_40_H_16_				C_40_F_16_				C_40_H_10_F_6_			
Parameters	RHF	B3LYP	wB97 XD	BPBE	RHF	B3LYP	wB97 XD	BPBE	RHF	B3LYP	wB97 XD	BPBE
EE (10^1^)	−152.437	−153.420	−153.368	−153.347	−310.594	−312.195	−312.025	−312.738	−211.749	−212.964	−212.897	−212.875
ZPVE (kcal/Mol)	284.635	267.225	270.311	260.617	198.985	184.976	187.662	179.652	253.227	236.942	240.173	230.751
E_T_ (kcal/mol)	297.602	281.178	284.209	275.110	221.029	208.548	211.090	203.991	269.454	254.382	257.463	248.804
U (10^−3^)	474.259	448.086	452.916	438.416	352.233	332.342	336.393	325.081	429.403	405.384	410.294	396.495
H_Th_ (10^−3^)	475.203	449.030	453.860	439.360	353.177	333.286	337.337	326.025	430.347	406.328	411.238	397.439
G_Th_ (10^−3^)	407.664	378.124	382.955	366.223	252.554	227.771	231.942	217.688	350.108	322.421	327.676	311.665
E1 (10^1^)	−152.392	−153.776	−153.257	−153.055	−310.562	−312.166	−312.073	−312.045	−211.709	−212.926	−212.858	−212.835
E2 (10^1^)	−152.390	−153.375	−153.323	−153.332	−310.558	−312.162	−312.068	−312.041	−211.706	−212.923	−212.856	−212.833
H (10^1^)	−152.390	−153.375	−153.323	−153.303	−310.558	−312.162	−312.068	−312.041	−211.706	−212.923	−212.856	−212.833
G	−152.397	−153.382	−153.330	−153.310	−310.568	−312.172	−312.079	−312.052	−211.715	−212.932	−212.864	−212.842
C_P_(Cal/mol/°K)	97.374	104.602	103.801	108.182	143.671	151.872	150.873	155.805	113.97	121.76	120.60	125.56
C_V_(Cal/mol/°K)	95.372	102.611	101.813	106.197	141.681	149.881	148.883	153.817	112.038	119.837	118.675	123.631
S(Cal/mol/°K)	142.149	149.234	149.234	153.929	211.778	222.076	221.823	228.013	168.877	176.597	175.871	180.527
Rotational constant	0.186	0.185	0.185	0.183	0.0712	0.0701	0.0705	0.069	0.096	0.095	0.095	0.094
kcal/mol	0.078	0.077	0.077	0.076	0.036	0.035	0.036	0.035	0.067	0.066	0.066	0.065
	0.055	0.054	0.055	0.054	0.024	0.024	0.024	0.023	0.0395	0.039	0.039	0.0386
$E_{Trans}$	0.889	0.889	0.889	0.889	0.889	0.889	0.889	0.889	0.889	0.889	0.889	0.889
$E_{Rot}$	0.889	0.889	0.889	0.889	0.889	0.889	0.889	0.889	0.889	0.889	0.889	0.889
$E_{Vib}$	295.824	279.401	282.432	273.333	219.252	206.770	209.312	202.214	267.677	252.605	255.686	247.027

**Table 4:** Quadratic correlation equations of the molecular structure at different temperature.

Molecules	Methods	Thermodynamic properties
C_40_H_16_	RHF	$G_{m}^{0}=-67.157\times 10^{-5}T^{2}-20.053\times 10^{-2}T+1189.615\left(R^{2}=0.99999\right)$
		$H_{m}^{0}=58.963\times 10^{-5}T^{2}+6.787\times 10^{-2}T+1178.750\left(R^{2}=0.99946\right)$
		$C_{p,m}^{0}=-83.285\times 10^{-5}T^{2}+199.412\times 10^{-2}T-102.933\left(R^{2}=0.99888\right)$
		$S_{m}^{0}=-7.331\times 10^{-5}T^{2}+139.953\times 10^{-2}T+192.606\left(R^{2}=0.99946\right)$
	B3LYP	$G_{m}^{0}=-70.941\times 10^{-5}T^{2}-20.839\times 10^{-2}T+1117.514\left(R^{2}=0.99999\right)$
		$H_{m}^{0}=59.835\times 10^{-5}T^{2}+9.271\times 10^{-2}T+1102.511\left(R^{2}=0.99936\right)$
		$C_{p,m}^{0}=-96.157\times 10^{-5}T^{2}+21.438\times 10^{-1}T-107.385\left(R^{2}=0.99907\right)$
		$S_{m}^{0}=-12.617\times 10^{-5}T^{2}+152.694\times 10^{-2}T+189.699\left(R^{2}=0.99945\right)$
	wB97XD	$G_{m}^{0}=-70.403\times 10^{-5}T^{2}-21.138\times 10^{-2}T+1130.603\left(R^{2}=0.99999\right)$
		$H_{m}^{0}=59.475\times 10^{-5}T^{2}+9.126\times 10^{-2}T+1121.824\left(R^{2}=0.99938\right)$
		$C_{p,m}^{0}=-93.942\times 10^{-5}T^{2}+211.443\times 10^{-2}T-104.051\left(R^{2}=0.99910\right)$
		$S_{m}^{0}=-33.496\times 10^{-5}T^{2}+187.912\times 10^{-2}T+211.048\left(R^{2}=0.99984\right)$
	BPBE	$G_{m}^{0}=-72.617\times 10^{-5}T^{2}-21.827\times 10^{-2}T+1090.548\left(R^{2}=0.99999\right)$
		$H_{m}^{0}=59.848\times 10^{-5}T^{2}+10.695\times 10^{-2}T+1073.130\left(R^{2}=0.99932\right)$
		$C_{p,m}^{0}=-1.023\times 10^{-3}T^{2}+220.025\times 10^{-2}T-105.884\left(R^{2}=0.99921\right)$
		$S_{m}^{0}=-15.991\times 10^{-5}T^{2}+159.415\times 10^{-2}T+192.342\left(R^{2}=0.99947\right)$
C_40_F_16_	RHF	$G_{m}^{0}=-87.426\times 10^{-5}T^{2}-36.823\times 10^{-2}T+847.927\left(R^{2}=0.99993\right)$
		$H_{m}^{0}=55.954\times 10^{-5}T^{2}+26.939\times 10^{-2}T+802.257\left(R^{2}=0.99943\right)$
		$C_{p,m}^{0}=-1.192\times 10^{-3}T^{2}+232.014\times 10^{-2}T+8.044\left(R^{2}=0.99939\right)$
		$S_{m}^{0}=-63.283\times 10^{-5}T^{2}+237.948\times 10^{-2}T+233.0198\left(R^{2}=1\right)$
	B3LYP	$G_{m}^{0}=-91.368\times 10^{-5}T^{2}-38.771\times 10^{-2}T+791.759\left(R^{2}=0.99991\right)$
		$H_{m}^{0}=55.880\times 10^{-5}T^{2}+30.411\times 10^{-2}T+740.179\left(R^{2}=0.99939\right)$
		$C_{p,m}^{0}=-1.281\times 10^{-3}T^{2}+24.181\times 10^{-1}T+20.523\left(R^{2}=0.99991\right)$
		$S_{m}^{0}=-72.364\times 10^{-5}T^{2}+255.078\times 10^{-2}T+232.307\left(R^{2}=0.99983\right)$
	wB97XD	$G_{m}^{0}=-90.734\times 10^{-5}T^{2}-39.032\times 10^{-2}T+802.953\left(R^{2}=0.99991\right)$
		$H_{m}^{0}=55.625\times 10^{-5}T^{2}+30.091\times 10^{-2}T+751.913\left(R^{2}=0.99994\right)$
		$C_{p,m}^{0}=-1.27\times 10^{-3}T^{2}+239.511\times 10^{-2}T+21.102\left(R^{2}=0.99905\right)$
		$S_{m}^{0}=-71.669\times 10^{-5}T^{2}+253.135\times 10^{-2}T+236.352\left(R^{2}=0.99999\right)$
	BPBE	$G_{m}^{0}=-93.095\times 10^{-5}T^{2}-40.216\times 10^{-2}T+770.921\left(R^{2}=0.99991\right)$
		$H_{m}^{0}=55.525\times 10^{-5}T^{2}+32.207\times 10^{-2}T+716.253\left(R^{2}=0.99937\right)$
		$C_{p,m}^{0}=-1.332\times 10^{-3}T^{2}+245.498\times 10^{-2}T+28.387\left(R^{2}=0.99882\right)$
		$S_{m}^{0}=-77.156\times 10^{-5}T^{2}+263.421\times 10^{-2}T+235.999\left(R^{2}=0.99999\right)$
C_40_H_10_F_6_	RHF	$G_{m}^{0}=-74.546\times 10^{-5}T^{2}-26.735\times 10^{-2}T+1064.111\left(R^{2}=0.99998\right)$
		$H_{m}^{0}=58.035\times 10^{-5}T^{2}+13.987\times 10^{-2}T+1040.812\left(R^{2}=0.99945\right)$
		$C_{p,m}^{0}=-96.267\times 10^{-5}T^{2}+211.565\times 10^{-2}T-63.612\left(R^{2}=0.99968\right)$
		$S_{m}^{0}=-27.295\times 10^{-5}T^{2}+175.292\times 10^{-2}T+214.017\left(R^{2}=0.99982\right)$
	B3LYP	$G_{m}^{0}=-78.446\times 10^{-5}T^{2}-27.701\times 10^{-2}T+997.463\left(R^{2}=0.99997\right)$
		$H_{m}^{0}=58.525\times 10^{-5}T^{2}+16.925\times 10^{-2}T+969.158\left(R^{2}=0.99937\right)$
		$C_{p,m}^{0}=-1.081\times 10^{-3}T^{2}+22.497\times 10^{-1}T-61.873\left(R^{2}=0.99972\right)$
		$S_{m}^{0}=-34.172\times 10^{-5}T^{2}+189.924\times 10^{-2}T+208.821\left(R^{2}=0.99999\right)$
	wB97XD	$G_{m}^{0}=-77.773\times 10^{-5}T^{2}-27.770\times 10^{-2}T+1010.884\left(R^{2}=0.99997\right)$
		$H_{m}^{0}=58.252\times 10^{-5}T^{2}+16.573\times 10^{-2}T+983.227\left(R^{2}=0.99939\right)$
		$C_{p,m}^{0}=-1.061\times 10^{-3}T^{2}+222.098\times 10^{-2}T-60.337\left(R^{2}=0.99974\right)$
		$S_{m}^{0}=-12.504\times 10^{-5}T^{2}+151.629\times 10^{-2}T+192.525\left(R^{2}=0.99947\right)$
	BPBE	$G_{m}^{0}=-80.189\times 10^{-5}T^{2}-28.308\times 10^{-2}T+972.388\left(R^{2}=0.99996\right)$
		$H_{m}^{0}=58.407\times 10^{-5}T^{2}+18.512\times 10^{-2}T+941.447\left(R^{2}=0.99934\right)$
		$C_{p,m}^{0}=-1.143\times 10^{-3}T^{2}+230.232\times 10^{-2}T-58.534\left(R^{2}=0.99972\right)$
		$S_{m}^{0}=-38.074\times 10^{-5}T^{2}+197.302\times 10^{-2}T+206.614\left(R^{2}=0.99985\right)$

From this thermodynamic property table, it can be seen that the values of ZPVE, EE, E_T_, U, H_Th_, G_Th_, E1, E2, H, G decrease when the virgin C_40_H_16_ molecule is doped with any functional. This decrease varies in the following order, C_40_F_16_ < C_40_H_10_F_6_ < C_40_H_16_. The values of G and H being all negative, confirm that our molecules are thermodynamically stable. The lowest values of G and H are respectively −312.172 Hartree and −312.162 Hartree and are given by the C_40_F_16_ molecule, with the wB97XD method and the highest values are respectively −153.330 Hartree and −153.323 Hartree and are given by the undoped C_40_H_16_ molecule These results show that the C_40_F_16_ molecule is more stable than the others and that the B3LYP method could be better used to study the thermodynamic properties of our compounds. It can also be seen from this table that, the values of C_P_, C_V_ and S increase considerably when the pristine molecule is spiked using the methods employed in this paper. This increase varies in the following order, C_40_H_16_ < C_40_H_10_F_6_ < C_40_F_16_. The highest values of, C_P_, C_V_ and S are given by the C_40_F_16_ molecule, with the BPBE method and then B3LYP. Similarly, the lowest values of C_P_, C_V_ and S are given by the undoped C_40_H_16_ molecule, with the RHF method and then wB97XD. It can be concluded that these high values of C_P_, C_V_ and S also confirm that the C_40_F_16_ molecule is the most stable among our molecules.

Thus, the values of the standard thermodynamic properties of these modeled molecular structures can be predicted at any other temperature on the basis of these equations. It can be seen from these curves that the constant pressure heat capacity ${(C}_{p,m}^{0})$, entropy ${(S}_{m}^{0})$, and enthalpy ${(H}_{m}^{0})$ increase significantly for all modeled compounds, while the Gibbs free energy $\left(G_{m}^{0}\right)$ decreases significantly. Furthermore, the Gibbs energy G increase, while the enthalpy H decrease, for temperatures ranging from 100K to 900K when going from the C_40_H_16_ molecule to the C_40_H_10_F_6_ molecule, and finally, to the C_40_F_16_ molecule. The G and H values of the studied compounds are all negative for all considered temperatures. Therefore, we can say that the synthesis reactions of these compounds are spontaneous and thermodynamically favorable for all temperatures.

### 3.5 Optoelectronic and nonlinear optical properties (NLO)

Understanding the properties of non-linear optics (NLO) is very important for the design of new materials for optical signal processing, communication technologies, electro-optical modulation in data storage. Polarizability provides information about the distribution of electrons in the molecule and plays a fundamental role in determining the structural and thermodynamic properties of a molecular system. First hyperpolarizability $(\beta_{mol}\text{,}$ non-linear response), second hyperpolarizability (γ, non-linear response), polarizability (α, linear response), and dipole moment (μ) are important parameters for evaluating the non-linear optical response of molecular materials [[45], [46], [47]]. The phenomenon of nonlinear optics has great potential in the field of optical telecommunications and optical information storage. The theoretical determination of first hyperpolarizability and dipole moment are very useful in understanding the relationship between molecular structure and nonlinear optical properties [48]. The space group for the undoped molecule is D_2h_, while for the doped molecules are C_2V_. Table 5 shows the linear optical, nonlinear optical and optoelectronic properties calculated. From Table 5 it can be seen that the linear polarizability (α) and anisotropy (Δα) increase with the number of carbon-fluorine (C–F) bonds in C_40_F_16_, and C_40_H_10_F_6_ molecules using the DFT/cc-pVDZ methods, but decrease when we use the RHF/cc-pVDZ method. It also appears that the polarizability (α) and anisotropy (Δα) of these molecules increase for the DFT methods in the order C_40_F_16_ > C_40_H_10_F_6_ > C_40_H_16_, and decrease for the RHF method in the order: C_40_F_16_ < C_40_H_10_F_6_ < C_40_H_16_. In general, a large linear polarization requires obtaining a large nonlinear hyperpolarizability [49,50]. Similarly, a smaller HOMO-LUMO energy gap (E_gap_) leads to a higher NLO response. Thus, decreasing the gap energy (E_gap_) of doped molecules leads to an increase in their electrical conductivity, which helps to understand why these materials are proposed as very good semiconductors. Organic molecules with high nonlinear optical properties are at the forefront of current research due to their use as optical modulation and memory materials for emerging technologies. The large values of the first hyperpolarizability and linear polarizability of the studied compounds are obtained on the BPBE and B3LYP methods. The second hyperpolarizability (γ) for all studied molecules is maximal on the B3LYP/cc-pVDZ method and then on the BPBE/cc-PVDZ method. For a given molecular system, when the values of the first hyperpolarizability (β_mol_) and dipole moment (μ) are higher than those of urea (μ = 3.8851D and β_mol_ = 372.8 × 10^−33^ esu), the said molecule has very good nonlinear optical properties [48,[51], [52], [53]]. The calculated values of β_mol_ and μ, for the molecules C_40_F_16_ and C_40_H_10_F_6_, are much higher than those of urea, regardless of the method used. The difference is estimated to be 783.43%, 1327.88%, 1177.29% and 1677.27% for the first hyperpolarizability and 74.93%, 24.77%, 44.67% and 7.22% for the dipole moment using RHF, B3LYP, wB97XD and BPBE functionals respectively for the C_40_H_10_F_6_ molecule. The same difference is estimated to be 419.53%, 475.99%, 412.26% and 231.39% for the first hyperpolarizability using RHF, B3LYP, wB97XD and BPBE functionals for the C_40_F_16_ molecule respectively. This result suggests that the studied systems with high hyperpolarizability open up another field of application of doped molecules (C_40_F_16_ and C_40_H_10_F_6_) in nonlinear optics, especially in telecommunications, information storage, optical communication and signal processing.

**Table 5:** The electric dipole moment (μ), average polarizability (α), anisotropy ($\Delta_{\alpha }$), first hyperpolarizability ($\beta_{mol}$), second hyperpolarizability (γ), Molar refractivity (MR), Volume (V), average Electric field (E), Polarization density (P), Electric susceptibility ($\chi_{e}$), relative dielectric constant ($\varepsilon_{r}$), dielectric constant (ε), refractive index (n), magnitude of the displacement vector (D), phase velocity (V_PH_), and magnetic field (B) of the molecules C_40_H_16_, C_40_F_16_ and C_40_H_10_F_6_ using RHF, B3LYP, wB97XD and BPBE methods by employing the cc-pVDZ basis set.

	C_40_H_16_				C_40_F_16_				C_40_H_10_F_6_			
Parameters	RHF	B3LYP	wB97XD	BPBE	RHF	B3LYP	wB97XD	BPBE	RHF	B3LYP	wB97XD	BPBE
μ (C.m) × 10^−33^	40.325	271.29	95.253	119.841	151.662	847.3712	847.3643	288.512	22652.134	16156.784	18733.526	13884.194
α (esu) × 10^−24^	72.035	81.415	76.872	84.938	71.085	83.887	78.719	89.081	71.117	81.569	76.847	85.618
Δα (esu) × 10^−24^	85.123	101.013	93.084	106.741	83.322	103.891	94.965	112.051	83.841	100.525	92.434	106.612
$\beta_{mol}$ (esu) × 10^−33^	32.824	122.872	112.849	142.948	1936.812	2147.321	1909.734	2686.72	3293.432	5323.143	4751.536	6621.945
γ (esu) × 10^−37^	−40.012	−40.693	−39.745	−40.491	−81.387	−79.012	−78.324	−77.965	−50.977	−50.984	−50.113	−50.661
MR × 10^−15^	20.211	22.843	21.568	23.831	19.944	23.536	22.086	24.994	19.954	22.886	21.561	24.022
V (m^3^) × 10^−30^	220.351	242.971	229.221	245.312	246.491	262.142	222.551	260.921	233.743	237.182	258.472	214.921
E (Vm^−1^) × 10^6^	11.138	29.513	12.682	50.172	21.918	39.079	32.911	59.675	145.754	219.109	178.051	286.294
P (C.m^−2^) × 10^−5^	18.301	46.602	41.563	48.856	1 6.153	38.325	11.058	32.076	681.345	969.132	645.984	724.723
$\chi_{e}$	4.108	4.210	4.214	4.351	3.624	4.021	4.445	4.289	3.823	4.321	3.736	5.005
$\varepsilon_{r}$	5.108	5.210	5.214	5.351	4.624	5.021	5.445	5.289	4.823	5.321	4.736	6.005
ε × 10^−11^	4.522	4.613	4.617	4.738	4.094	4.445	4.821	4.684	4.270	4.712	4.193	5.317
n	2.261	2.282	2.283	2.313	2.150	2.241	2.333	2.301	2.196	2.307	2.176	2.451
D (C.m^−2^) × 10^−5^	50.752	136.703	58.425	238.029	89.732	173.706	158.635	279.517	622.369	1032.441	746.567	1522.225
V_PH_ (ms^−1^) × 10^6^	132.742	131.428	131.384	129.694	139.518	133.885	128.570	130.436	136.604	130.050	137.857	122.418
B (Vm^−2^s)	0.0379	2.279	0.0847	0.0978	0.013	0.678	0.752	0.022	2.096	1.3691	1.589	1.191

We observed that the trends in the gap energy (E_gap_) evolve in opposite directions with respect to the NLO properties of these molecules. This means that, for a small electronic gap, large values of the first hyperpolarizability (β_mol_) and second hyperpolarizability (γ) are obtained. This shows that doping further decreases the gap energy (E_gap_), but on the other hand, it increases the NLO properties. Thus, we can conclude that the increase of the NLO properties leads to the increase of the reactivity of the studied molecules. In Table 5, we see that the values of the average electric field (E) and the magnetic displacement (D) evolve as a function of the methods for all the molecules studied in the following order: RHF < wB97XD < B3LYP < BPBE. The average electric field E and the magnetic displacement D are larger for the BPBE/cc-pVDZ method and smaller for the RHF/cc-pVDZ method. From this study, it can be seen that as E and D increase, the HOMO-LUMO energy gap decreases; this facilitates the transport of charge (electrons) from the HOMO orbital to the LUMO orbital and consequently, improves the NLO properties of the materials.

### 3.6 Electronic properties

The energy gap and electrical conductivity are important parameters in the study of electronic properties of organic materials. The energy gap is also a key parameter that provides information about the reactivity and stability of molecules or a molecular system. Molecules with a very large E_gap_ are hard and have a very good chemical stability, while molecules with a low energy gap are chemically more polarizable, more reactive, and flexible have low chemical stability [54]. Table 6 summarized the values of the electronic properties of C_40_H_16_, C_40_F_16_, and C_40_H_10_F_6_ molecules obtained using the RHF, B3LYP, wB97XD, and BPBE functionals. These calculations are evaluated using the cc-pVDZ basis. These values of electronic properties of circumanthracene molecule are obtained from the mathematical equation found in literature [16,55,56]. The results show that the gap energy decreases when doped with fluorine atoms for each functional (B3LYP, BPBE, and wB97XD) used and remains below 4 eV. This leads to the conclusion that the studied molecules are soft, polarizable, more reactive and have low chemical stability. We also noticed that the C_40_F_16_ molecule has a smaller energy gap than the other molecules studied. This molecule is therefore more reactive than the others. It also appears from this study that for all the molecules studied, the energy gap is smaller when the BPBE functional is used followed by that obtained when the B3LYP functional used and then that of wB97XD functional. Thus, we can say that regardless of the molecule studied, the energy gap decrease is in the following order, RHF < wB97XD < B3LYP < BPBE. The gap energy (E_gap_) of C_40_H_16_ and C_40_F_16_ molecules have been calculated by Roberto et al. [19], Paola et al. [57] using the B3LYP/6-31+G* method. They reported in their different studies that the energy gap is 2.14 eV and 2.03 eV respectively for C_40_H_16_ and C_40_F_16_ molecules. These values were found to be almost identical to those in this study with the B3LYP/cc-pVDZ method. The difference is between 0.005 and 0.01 eV, this shows that the basis set used has little impact on the energy gap. These small values of the calculated energy gaps suggest that our modeled systems have high electrical conductivity. These results further suggest that our modeled materials are very good semiconductors and can be used in the design of organic solar cells, OLEDs, and many other electronic devices. The calculated values of the adiabatic ionization energy (IE_A_), vertical ionization energy (IE_V_), adiabatic electron affinity (EA_A_), vertical electron affinity (EA_V_), quasiparticle corrected energy band gap ($Q_{gap}^{1}$), transition excitation energy (E_opt_), and exciton binding energy (E_bind_) of the C_40_H_16_ molecule using the B3LYP/6-31+G* method are respectively; 5. 90, 5.94, 1.68, 1.63, 4.31, 2.03 and 2.28 eV [19,57] while those obtained in this study for the same molecule using the B3LYP/cc-pVDZ method are 5.847, 5.884, 1.583, 1.536, 4.347, 2.027 and 2.320 eV respectively. The calculated values of the adiabatic ionization energy (IE_A_), vertical ionization energy (IE_V_), adiabatic electron affinity (EA_A_), vertical electron affinity (EA_V_), quasiparticle corrected energy band gap ($Q_{gap}^{1}$), transition excitation energy (E_opt_) and exciton binding energy (E_bind_) of the C_40_F_16_ molecule, using the B3LYP/6-31+G* method, are respectively: 6. 98, 7.06, 2.85, 2.93, 4.21, 1.94 and 2.27 eV [19] while those obtained in this study for the same molecule, using the B3LYP/cc-pVDZ method, are respectively: 6.649, 6.730, 2.569, 2.448, 4.079, 1.929, and 2.313 eV. Comparing the results obtained by the two methods, we observe a difference of the order of 0.053, 0.056, 0.097, 0.094, 0.037, 0.003, and 0.04 eV respectively for IE_A,_ IE_V,_ EA_A,_ EA_V_, $Q_{gap}^{1}$, E_opt_ and E_bind_. It can be seen from this study that our theoretical results are in good agreement with those obtained in the literature [19,57] using the B3LYP method. Thus, regardless of the base and functional used, the electronic properties vary slightly. In Table 6, we see that the large values of adiabatic ionization energy (IE_A_), vertical ionization energy (IE_V_), adiabatic electron affinity (EA_A_), vertical electron affinity (EA_V_), quasiparticle corrected energy band gap ($Q_{gap}^{1}$), transition excitation energy (E_opt_), and exciton binding energy (E_bind_) are obtained for the C_40_F_16_ molecule for all methods. This shows that an increase in IE_A,_ IE_V,_ EA_A,_ EA_V_, $Q_{gap}^{1}$, E_opt_, E_bind_ and a decrease in gap energy occur as the number of fluorine atoms used in doping increases. The substitution of halogen atoms (F, Cl) in polyaromatic hydrocarbons (PAHs) can be interpreted in terms of modification of chemical bonds and electron distribution in the molecule. In fact, when C–H bonds are replaced with C–F bonds, one can expect a more polarizable, more reactive, and less stable molecule than the undoped molecule, which correlates with the reduction of the HOMO-LUMO gap that occurs in some halogen-substituted aromatic compounds.

**Table 6:** Electronic properties for the C_40_H_16_, C_40_F_16_, and C_40_H_10_F_6_ molecules using RHF, B3LYP, wB97XD, and BPBE methods by employing the cc-pVDZ basis set. All data are given in eV.

	C_40_H_16_				C_40_F_16_				C_40_H_10_F_6_			
Parameters	RHF	B3LYP	wB97XD	BPBE	RHF	B3LYP	wB97XD	BPBE	RHF	B3LYP	wB97XD	BPBE
E_LUMO_	0.315	−2.643	−1.295	−3.125	−0.924	−3.601	−2.271	−3.896	−0.110	−3.012	−1.655	−3.436
E_HOMO_	−4.891	−4.778	−5.255	−4.394	−6.202	−5.621	−6.094	−5.041	−5.352	−5.093	−5.561	−4.636
E_gap_	**5.207**	**2.135**	**3.959**	**1.269**	**5.277**	**2.020**	**3.822**	**1.144**	**5.242**	**2.081**	**3.906**	**1.199**
${IE}_{A}$	4.378	5.847	6.001	5.838	5.804	6.649	6.806	635	4.973	6.143	6.299	6.057
${IE}_{V}$	4.671	5.884	6.079	5.860	5.181	6.730	6.943	6.494	5.281	6.206	6.409	6.097
${EA}_{A}$	0.036	1.583	1.555	1.692	2.477	2.569	2.554	2.495	1.562	1.961	1.931	2.004
${EA}_{V}$	0.701	1.536	1.463	1.663	2.071	2.488	2.426	2.433	1.241	1.899	1.829	1.963
$Q_{gap}^{1}$	3.970	4.347	4.616	4.197	3.111	4.242	4.517	4.061	4.040	4.306	4.579	4.134
$E_{gap}^{KS}$	3.393	4.264	4.445	4.146	3.327	4.079	4.251	3.940	3.410	4.182	4.368	4.053
$E_{opt}$	2.810	2.027	2.449	1.762	2.873	1.929	2.367	1.628	2.835	1.965	2.405	1.664
$E_{bind}$	1.160	2.320	2.167	2.435	0.238	2.313	2.150	2.433	1.205	2.341	2.174	2.701

### 3.7 Global chemical reactivity descriptors

Global reactivity descriptor parameters of C_40_H_16_, C_40_F_16_, and C_40_H_10_F_6_ molecules are evaluated using RHF, B3LYP, wB97XD, and BPBE with the cc-pVDZ basis set. The results of these parameters are given in Table 7 and allow us to determine the stability and reactivity of the studied molecular systems. The mathematical equations that allowed the calculation of these parameters are given in the literature [55,58]. From these results, it can be seen that regardless of the functional used that the nucleophilicity decreases from the C_40_H_16_ molecule to the C_40_F_16_ molecule; indicating that the C_40_H_16_ molecule is more nucleophilic compared to the other molecules. Therefore, C_40_H_16_ is the most reactive compound, followed by C_40_H_10_F_6_ and C_40_F_16_ is the least reactive compound. The reactivity of a molecule increases as the chemical potential (μ) decreases. Table 7 shows that the reactivity of the molecules increases as we move from the uncorrelated to the correlated level in the following order: RHF < B3LYP < BPBE < wB97XD. It is observed that the C_40_F_16_ molecule is more reactive than the C_40_H_10_F_6_ and C_40_H_16_ molecules because it has the lowest chemical potential values. This is because C_40_F_16_ has more C–F chemical bonds than C_40_H_10_F_6_. Chemical hardness is a parameter that characterizes the chemical stability of a molecule. A system with high chemical hardness is considered very stable. Thus, molecules with low chemical hardness are more reactive, polarizable, and less stable. The hardness is also a way to measure the stability of a chemical system in terms of its deformation. Thus, based on the B3LYP/cc-pVDZ, BPBE/cc-pVDZ, and wB97XD/cc-pVDZ functional, it is observed that the C_40_H_16_ molecule is more stable because it has the greatest chemical hardness, while the C_40_F_16_ and C_40_H_10_F_6_ molecules are more reactive and less stable, and tend to change under the effect of an external deformation. In terms of softness (σ), it follows the opposite trend of chemical hardness. The C_40_F_16_ molecule is the softest material using DFT methods, with softness values of 0.989, 0.415, and 1.747 eV for the B3LYP, wB97XD, and BPBE, functionals respectively. The undoped C_40_H_16_ molecule is the least soft as the softness values are 0.937, 0.403, and 1.576 eV, respectively, for B3LYP, wB97XD and BPBE functionals. Regarding the electronegativity, we found that the C_40_F_16_ molecule has the largest values of electronegativity regardless of the method used. Therefore, this molecule is a good electron acceptor. The maximum charge transfer (ΔN_max_) of a molecular system represents its maximum ability to acquire additional charge around in its environment. The maximum charge transfer (ΔN_max_) of the molecules increases in the following order: RHF ˂ wB97XD ˂ B3LYP ˂ BPBE. After doping with fluorine, which is a nucleophile, it is observed that the new systems show very good ability to acquire additional electronic charge, making them very reactive. The large values of the maximum charge transfer are obtained with the C_40_F_16_ molecule and then followed by C_40_H_10_F_6_.

**Table 7:** Global chemical reactivity descriptor: ionization potential (IP), electron affinity (EA), electronegativity (χ), chemical potential (μ), chemical hardness (η), chemical softness (σ), electrophilicity index (ω), nucleophilicity index (ν), maximum charge transfert (${\Delta N}_{\max}$), nucleofuge (${\Delta Ε}_{n}$), and electrofuge (${\Delta Ε}_{e}$) of the molecules C_40_H_16_, C_40_F_16_ and C_40_H_10_F_6_ using RHF, B3LYP, wB97XD, and BPBE methods by employing the cc-pVDZ basis set.

	C_40_H_16_				C_40_F_16_				C_40_H_10_F_6_			
Parameters	RHF	B3LYP	w97XD	BPBE	RHF	B3LYP	wB97XD	BPBE	RHF	B3LYP	wB97XD	BPBE
IP (ev)	5.891	4.778	5.255	4.394	7.202	5.621	6.094	5.041	6.352	5.093	5.561	4.635
EA (ev)	−0.315	2.643	1.295	3.125	0.924	3.601	2.271	3.896	0.110	3.012	1.655	3.436
χ (ev)	2.788	3.710	3.775	3.759	4.063	4.611	4.682	4.468	3.231	4.052	4.108	4.036
μ (ev)	−2.788	−3.710	−3.775	−3.759	−4.063	−4.611	−4.682	−4.468	−3.231	−4.052	−4.108	−4.036
η (ev)	3.103	1.068	2.479	0.634	3.138	1.012	2.411	0.572	3.121	1.040	2.453	0.599
σ (ev)^−1^	0.322	0.937	0.403	1.576	0.319	0.989	0.415	1.747	0.320	0.961	0.408	1.667
ω (ev)	1.253	6.447	2.874	11.140	2.629	10.522	4.547	17.442	1.672	7.892	3.440	13.580
ν (ev)^−1^	0.798	0.155	0.348	0.789	0.380	0.095	0.220	0.573	0.598	0.127	0.291	0.674
${\Delta N}_{\max}$	0.898	3.475	1.522	5.926	1.294	4.564	1.942	7.807	1.035	3.895	1.675	6.729
${\Delta Ε}_{n}$ (ev)	0.016	3.271	0.3384	7.697	0.136	6.417	1.069	13.260	0.002	4.359	0.558	9.844
${\Delta Ε}_{e}$ (ev)	5.592	10.692	7.889	15.217	8.262	15.638	10. 435	22.197	6.464	12.465	8.775	17.916

### 3.8 Vibrational analysis

Vibrational frequency analysis of a molecular system is a widely used method in materials science and organic chemistry to identify different functional groups in organic compounds. This method provides information on the molecular stability as well as on the motion of an atom or a group of atoms in a given system and for a specific frequency. The circumanthracene molecule is composed of 56 atoms, so it has 162 normal vibrational modes according to the nonlinear vibrational degree of freedom (3N-6) [59] for a nonlinear molecule, it belongs to the C_2V_ point group. The 162 modes of vibration consist of 55 stretching modes, 54 bending modes, and 53 twisting modes. The 162 fundamental vibrational modes of the molecule can be divided into 109 in-plane vibrations of the A′ species and 53 out-of-plane vibrations of the A″ species; thus, in terms of the irreducible representation, the vibrations are $T_{vib}=109A^{\prime }+53A^{\prime \prime }$. In general, the calculated theoretical harmonic wavenumbers are higher than the corresponding experimental values, due to deficiencies in the chosen basis sets and electronic correlation effects. In addition, the calculated wavenumbers are adjusted to be identical to those observed experimentally by applying a uniform scaling factor [60]. The harmonic vibrational frequencies (unscaled and scaled) calculated from RHF, B3LYP, wB97XD, and BPBE functionals, their probable assignments and their potential energy distributions (PEDs) with some experimental frequencies observed in the C_40_H_16_ identical aromatic compounds are presented in Table S4 (supplementary material). The vibrational analyses interpreted with the PED method are analyzed using the B3LYP/cc-pVDZ method.

Aromatic compounds generally exhibit very low wavenumbers in the [3100-3000] cm^−1^ region, this is due to the aromatic C–H stretching vibration [[61], [62], [63], [64]]. These bands are rarely very useful because they overlap with each other, allowing for higher absorption in this region. In this study, the calculated symmetric and antisymmetric scaled vibrations attributed to C–H stretching are in the ranges 3080, 3078, 3063, 3061, 3059, 3057, and 3058 cm^−1^ with the PED contributions of 100%, 88%, 79%, 73%, 74%, 70%, and 85%, respectively. The in-plane and out-of-plane C–H bending modes of the benzene ring are in the [1500-1000] cm^−1^ and [1000-700] cm^−1^ ranges, respectively [62,65]. In the case of C_40_H_16_ molecule, the peaks 1454, 1415, 1333, 1258, 1267, 1223, 1212, 1182, 1155, 1142, and 1039 cm^−1^ are attributed to in-plane C–H bending vibrations. The calculated wave numbers of the molecule at 989, 986, 964, and 971 cm^−1^ are attributed to out-of-plane stretching vibrations. These vibrations show good agreement with the values of aromatic compounds found experimentally in literature [64].

**Fig. 5:**
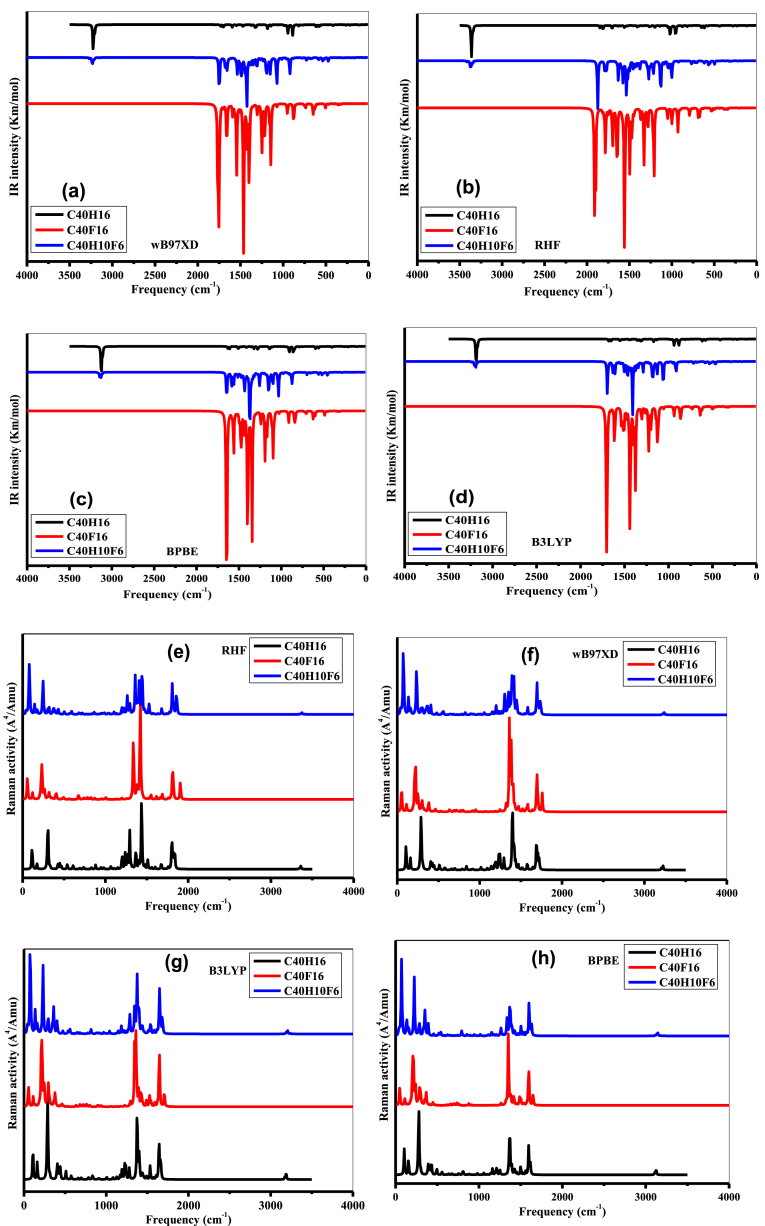
Raman Spectrum (a to d) and IR Spectrum (e to h) for the C_40_H_16_, C_40_F_16_, and C_40_H_10_F_6_ molecules obtained with B3LYP, BPBE, wB97XD, and RHF by employing cc-pVDZ basis set.

The C–C stretching vibrations of the benzene ring occur in the region [1625-1430] cm^−1^ [62,66]. In the present work, the C–C stretching vibrations are assigned to 1540, 1493, 1452, 1398 and 1370 cm^−1^. These theoretically calculated values are in good agreement with the experimental values [67,68] with contributions of 16%, 28%, 14%, and 17%, respectively. Moreover, some values found experimentally at 1527, 1493, 1463,1430, 1415 Cm^-1^ in FT-IR and at 1562, 1497, 1462, 1459 and 1390 cm^−1^ in FT-Raman are close to the theoretical values of our compound. The C–C–C planar wave number of an aromatic compound is reported in the literature in the range [999-665] cm^−1^ [62,69]. For the C_40_H_16_ molecule, the C–C–C planar ring vibrations are predicted at 986, 967, 815, 777, 573 cm^−1^ for the B3LYP/cc-pVDZ level. However, these vibrations are observed experimentally at 949, 826 and 594 cm^−1^ in FT-IR and 859, 870 cm^−1^ in FT-Raman. The vibrational frequency analysis is thus performed to verify the stability of the optimized structures. Fig. 5 shows the IR (Fig. 5a to 5 d) and Raman (Fig. 5e to 5 h) spectra of C_40_H_16_, C_40_F_16_, and C_40_H_10_F_6_ molecules. These spectra show that no imaginary frequency is observed (no negative frequency value). This means that a local minimum was reached at the end of the optimization. From these curves, it appears that in the presence of fluorine atoms, the stretching vibrations show weak peaks but with slightly different frequencies than in the absence of fluorine atoms. This is due to the fact that fluorine atoms are very heavy and very electronegative than hydrogen atoms and therefore prevent symmetric and antisymmetric stretching between the C–F bonds. The C–F stretching vibration is experimentally observed in the range [1045, 1174] cm^−1^ [70]. In the study of the C_40_F_16_, and C_40_H_10_F_6_ molecule, we obtained some stretching vibrations at 1049, 1119, 1135, 1145 and 1219 cm^−1^ in FT-IR. In this study, we found that the C–F vibration can influence the C–H and C–C vibrations in a molecule. This influence depends on bond strength, bond length and the nature of adjacent functional groups. If the C–F vibration is intense, this can alter the vibrational characteristics of the C–H and C–C atoms, leading to changes in the observed vibrational frequencies. It should be noted that this influence can vary according to the specific nature of the substituent groups and their spatial arrangement.

### 3.9 NBO analysis

NBO analysis has been carried out for doped molecules (C_40_F_16_, and C_40_H_10_F_6_) and the undoped molecule (C_40_H_16_) at the DFT/B3LYP/cc-PVDZ level, to explain some of the intramolecular and intermolecular interactions, charge transfer and conjugate interactions that lead to the decrease in the stabilization energy of a molecule. Natural bond orbital analysis is an effective method for studying intra- and intermolecular bonding, interactions between bonds, and also provides a practical basis for studying charge transfer or conjugate interaction in molecular systems [22]. The second-order Fock matrix has been performed to estimate (evaluate) donor-acceptor interactions in NBO studies [68]. For each donor (i), and acceptor (j), the stabilization energy E (2) associated with delocalization i→ j is evaluated by the mathematical formula given by Equation (10).

(10) $$E\left(2\right)=\Delta E_{ij}=q_{i}\frac{F{\left(i,j\right)}^{2}}{\varepsilon_{j}-\varepsilon_{i}}$$

where $q_{i}$ represents the occupancy state of the donor orbital, $\varepsilon_{i}$ and $\varepsilon_{j}$ represent the orbital energy values of the acceptor and donor respectively, and F (i, j) is the element of the Fock matrix. In this study, the NBO analysis of donor-acceptor interactions and the stabilization energies of our compounds are presented in Tables S5, S6, and S7 (supplementary material).

Table S5 presents the analysis of C_40_H_16_ donor-acceptor interactions. In this table, the interactions C_8_–C_9_ → C_4_–C_5_ and C_7_–C_10_ → C_1_–C_6_ have the highest stabilization energy value E (2) which is 22.68 kcal/mol in the π→π* transition. The intramolecular hyperconjugative interaction of σ (C_18_–H_42_) distributes to σ*(C_7_–C_19_), (C_38_–C_39_) leading to a stabilization energy 5.23 kcal/mol. Furthermore, the second largest contribution to the stabilization energy was obtained for the interaction π C_7_–C_10_) → LP1 (C_19_) with an energy 50.87 kcal/mol. On the other hand, the resonance interactions LP1 (C_19_) → π* C_7_–C_10_, LP1 (C_19_) → π* C_17_–C_18_ and LP1 (C_19_) → π* C_20_–C_22_ contribute most to the stabilization energy with an energy of 65.55 kcal/mol, 78.32 kcal/mol and 48.27 kcal/mol respectively.

The calculated E (2) values of the molecule doped with 100% fluorine (C_40_F_16_) are presented in Table S6. In this table, C_4_–C_5_ presents the highest E (2) value, which is 50.82 kcal/mol in the π → LP1 (C_36_) transition. We also find that the lone pairs in the fluorine atom participate in the stabilization of the molecule through LP3 (F_47_) to σ* (C_39_–C_40_), LP3 (F_50_) to π* (C_32_–C_33_) and LP3 (F_54_) to π* (C_26_–C_27_) interactions with the high value of E (2) which is 21.10 kcal/mol. On the other hand, the LP2 (F_55_) → σ* (C_15_–C_17_) and LP2 (F_47_) → σ* (C_38_–C_39_) resonance interactions also have a significant contribution to the stabilization energy 7.44 kcal/mol. The highest E (2) value 74.59 kcal/mol is shown in the LP1 (C36) to π* (C34–C35), (C37–C38) transition. The π π (C_4_–C_5_) → π*(C_8_–C_9_) interaction also has a remarkable contribution to the stabilization energy of 22.68 kcal/mol. For the transitions C_37_–C_38_ (σ) to C_8_–C_38_ (σ*); C_18_–C_19_ (σ) to C_7_–C_19_ (σ*) and C_15_–C_16_ (σ) to C_11_–C_16_ (σ*), the highest stabilization energy is 4.26 kcal/mol.

The NBO study of C_40_H_10_F_6_ was also carried out and the results of the second-order perturbation energy analysis are presented in Table S7. The C_9_–C_10_→C_8_–C_38_ interaction shows a high stabilization energy E (2) which is 23.57 kcal/mol in the π to π* transition. The π to π* and σ to σ* transitions in doped molecules are responsible for the high values of nonlinear optical properties (NLO) and stabilization of the molecule concerned.

## 4 Conclusion

In this work, we have presented a systematic study of circumanthracene molecule doped with fluorine atoms using ab-initio, DFT, and TD-DFT methods with the cc-pVDZ basis set. We calculated the adiabatic and vertical electronic affinities, the adiabatic and vertical ionization energies, the molecular reorganization energies, quasiparticle corrected energy, kohn-Sham gap energy from the ground state structural relaxations with the RHF, B3LYP, wB97XD and BPBE methods. We found good agreement with experimental and theoretical data. The calculated reorganization energies are relatively lower for the molecule circumanthracene than for its fluorine-doped counterparts; this suggests a better charge transfer property and technological application of the C_40_H_16_ molecule. We observed an increase in reorganization energy of the molecules C_40_F_16_, and C_40_H_10_F_6_ as a result of the chemical modification which could deteriorate the transport properties of the undoped molecule. We found very large ionization energies and electron affinities in our results after total or partial substitution of hydrogen atoms with fluorine in the circumanthracene molecule. These trends are reflected in a reduction in the energy gap upon chemical modification. Regarding the optical properties, we calculated the electronic absorption spectra using the TD-DFT method to obtain the transition excitation energy and the excitons binding energy. TD-DFT calculations show that the maximum χ_max_ values are all related to the H→L transition all around environment. We found that the excitons binding energy remains almost unchanged when doped with fluorine. The gas-phase UV–Vis spectrum was also recorded by TD-DFT, and the value were in good agreement with the experimental values. The results of our work and those reported in literature using the B3LYP/cc-pVDZ method have shown that the perfluorination effect on the circumanthracene molecule increases the hole and electron reorganization energies, the vertical and adiabatic affinities and ionization energies, the optical properties, the optoelectronic and nonlinear optical properties, the transition excitation energy and the reactivity indices. In addition, NBO confirmed that the strongest intramolecular interaction was 78.32 kcal/mol via LP1 C_1_ to π* (C_17_–C_18_). Thus, NBO analysis reveals that hyper-conjugative interactions contribute strongly to the molecular stability of our compounds. The HOMO-LUMO energy gap obtained with the B3LYP and BPBE functionals; allows us to conclude that circumanthracene and its derivatives are good semiconductor materials that can be used in optoelectronic devices, telecommunications, electronics, LEDs, photonic materials and organic field effect transistors. The first hyperpolarizability of C_40_F_16_, and C_40_H_10_F_6_ molecules were to be 13.27 and 4.76 times respectively, higher than that of urea with the B3LYP/cc-PVDZ method, implying their potential application as efficient NLO materials. The purpose of the effect of halogen (fluorine) on the studied compounds is to help us to make a good choice of molecules for the design of new optoelectronic devices and photo photovoltaic materials. The relationship between the calculated statistical thermodynamic functions and the different temperatures can provide useful information on chemical kinetic and equilibrium studies in the future for the same molecules.

## Code availability

The calculations have been carried out using Gaussian 09 and GaussView Version 5 provided by Gaussian, Inc.

## Authors contribution

L. Fomekong Tsague, G. W. Ejuh: Conceived and designed the experiments; Performed the experiments; Analyzed and interpreted the data; Contributed reagents, materials, analysis tools or data; Wrote the paper.

A. Teyou Ngoupo: Analyzed and interpreted the data; Contributed reagents, materials, analysis tools or data, Wrote the paper.

Y. Tadjouteu Assatse, R. A. Yossa Kamsi, M. T. Ottou Abe: Contributed reagents, materials, analysis tools or data, Wrote the paper.

J. M. B. Ndjaka: Analyzed and interpreted the data; Contributed reagents, materials, analysis tools or data.

## Funding statement

This research did not receive any speciﬁc grant from funding agencies in the public, commercial, or not-for-proﬁt sectors.

## Data availability statement

Data included in article/supplementary material/referenced in article.

## Additional information

No additional information is available for this paper.

## Declaration of competing interest

The authors declare that they have no known competing financial interests or personal relationships that could have appeared to influence the work reported in this paper.
