# Supplementary material for: Ab-initio and density functional theory (DFT) computational study of the effect of fluorine on the electronic, optical, thermodynamic, hole and electron transport properties of the circumanthracene molecule
Source: Heliyon. 2023 Sep 3;9(9):e19647. doi: 10.1016/j.heliyon.2023.e19647 (PMC10558915; doi:10.1016/j.heliyon.2023.e19647)
Supplement: Multimedia component 1 [file mmc1.docx]

**Ab-initio and density functional theory (DFT) computational study of the effect of fluorine on the electronic, optical, thermodynamic, hole and electron transport properties of the circumanthracene molecule.**

L. Fomekong Tsague^1^, G. W. Ejuh^2, 3^, A. Teyou Ngoupo^1^, Y. Tadjouteu Assatse^1^, R. A. Yossa Kamsi^1^, M. T. Ottou Abe^1^, and J. M. B. Ndjaka^1^.

^1^*University of Yaoundé I, Faculty of Science, Department of Physics, P.O. Box 812 Yaoundé, Cameroon.*

^2^*University of Bamenda, National Higher Polytechnic Institute, Department of Electrical and Electronic Engineering, P. O. Box 39 Bambili, Cameroon.*

^3^*University of Dschang, IUT-FV Bandjoun, Department of General and Scientific Studies, P.O. Box 134, Bandjoun, Cameroon.*

**Corresponding author:** L. Fomekong Tsague (fomekongludovic@yahoo.fr)

***Supplementary Materials***


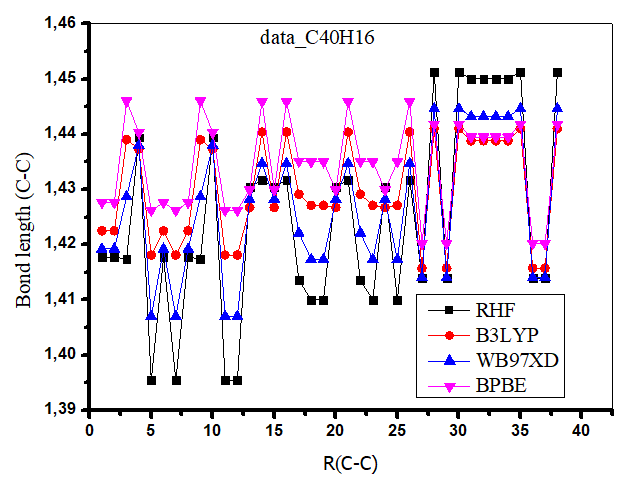

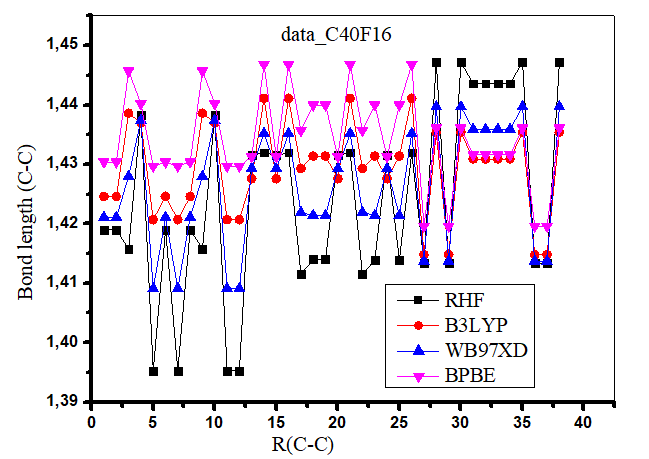

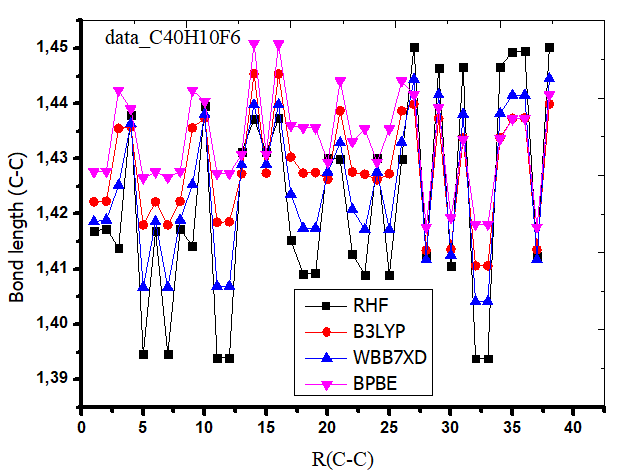

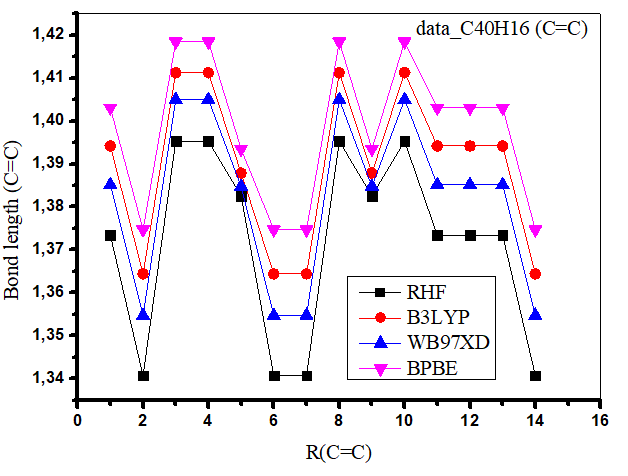

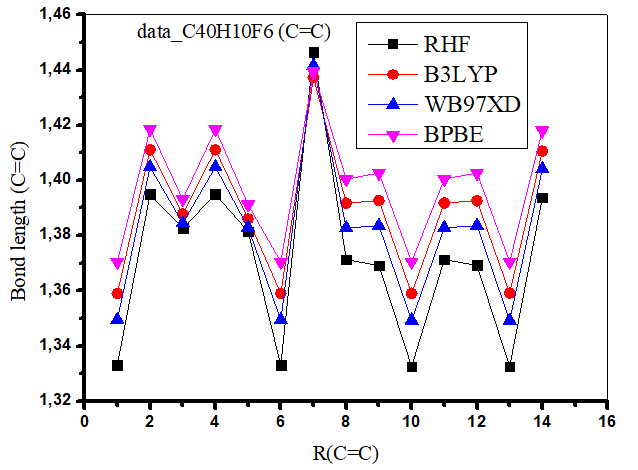

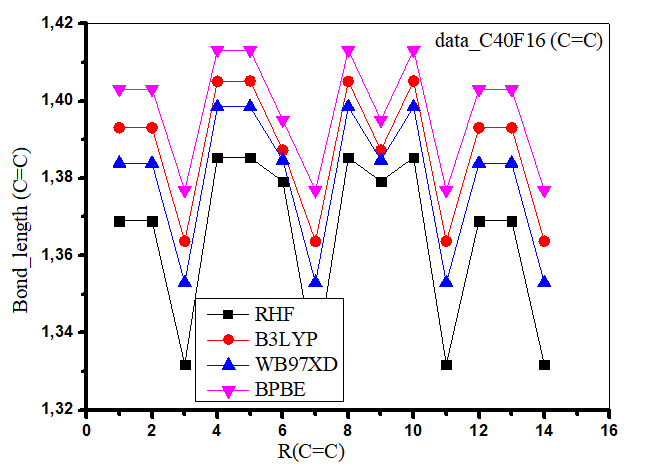


Figure S1. Graphical representation of the bond lengths and angles of the C_40_H_16_, C_40_F16, and C_40_H_10_F_6_ molecules obtained with the RHF, B3LYP, wB97XD, and BPBE methods by employing cc-pVDZ basis sets.


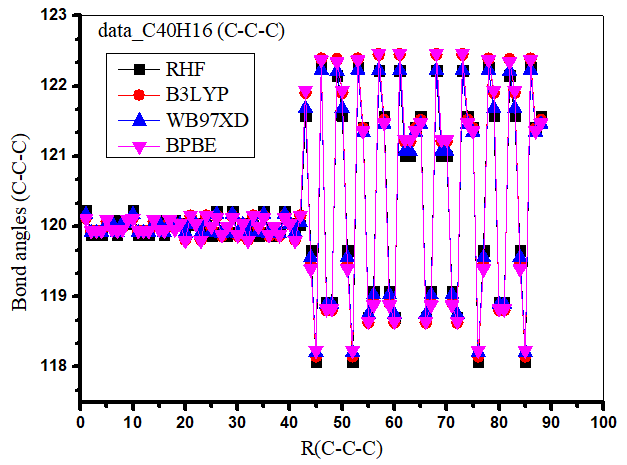

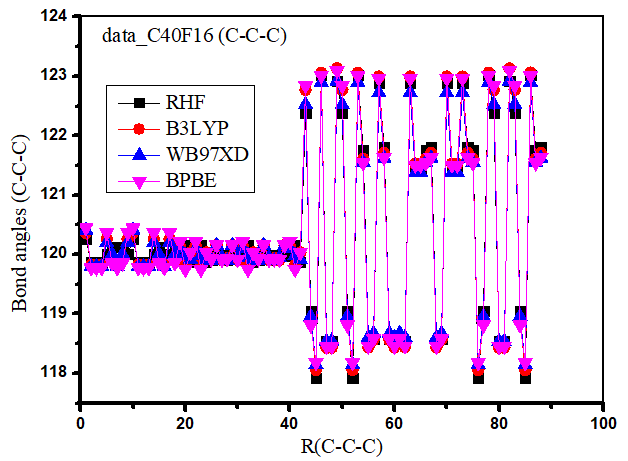

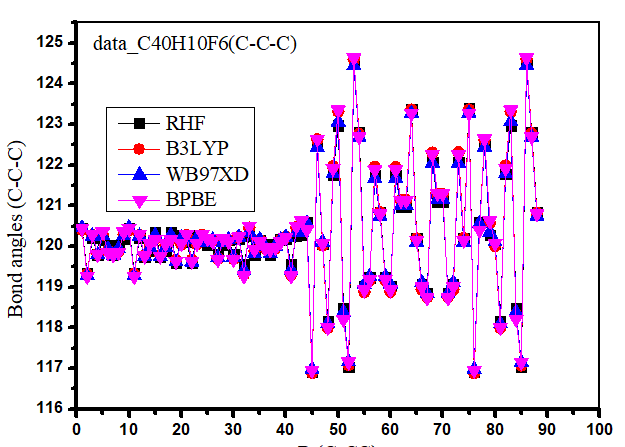

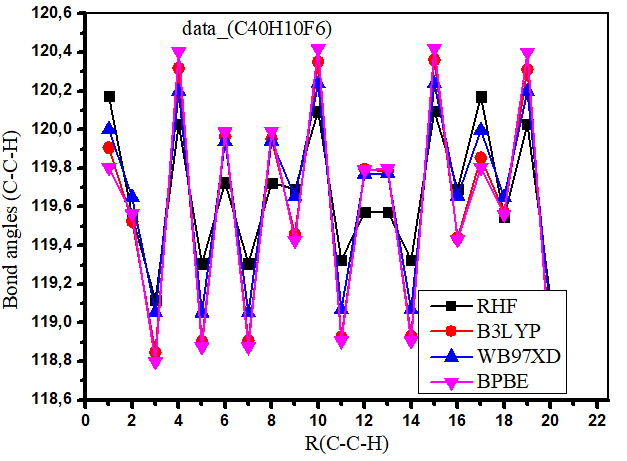

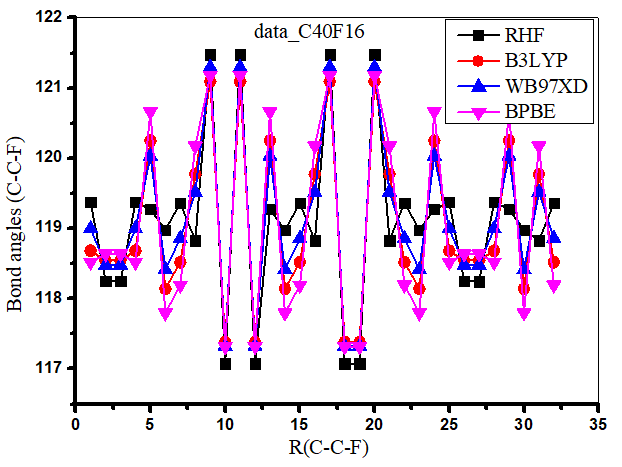

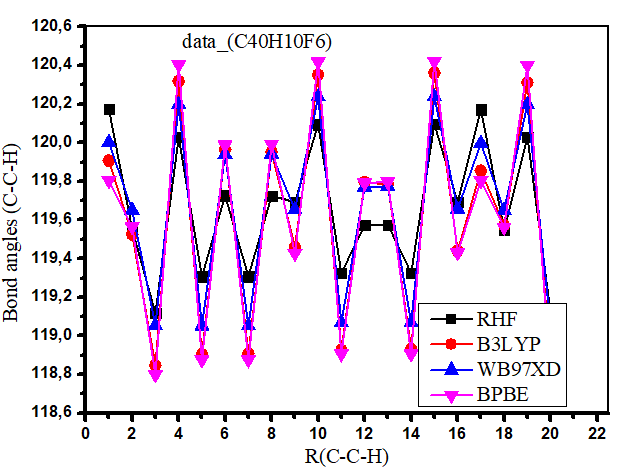


Table S1: Optimized geometrical parameters of the C_40_H_16_, C_40_F16, and C_40_H_10_F_6_ molecules obtained with the RHF, B3LYP, wB97XD, and BPBE methods by employing cc-pVDZ basis sets. Bond lengths are given Armstrong (Å).

| Bond lengths of C_40_H_16_ | | | | | Bond lengths of C_40_F16 | | | | | Bond lengths of C_40_H_10_F_6_ | | | | |
| --- | --- | --- | --- | --- | --- | --- | --- | --- | --- | --- | --- | --- | --- | --- |
|  | RHF | B3LYP | wB97  XD | BPBE |  | RHF | B3LYP | wB97  XD | BPBE |  | RHF | B3LYP | wB97  XD | BPBE |
| R(C1-C2) | 1.4177 | 1.4225 | 1.4192 | 1.4276 | R(C1-C2) | 1.419 | 1.4246 | 1.4211 | 1.4304 | R(C1-C2) | 1.4169 | 1.4222 | 1.4186 | 1.4276 |
| R(C1-C6) | 1.4177 | 1.4225 | 1.4192 | 1.4276 | R(C1-C6) | 1.419 | 1.4246 | 1.4211 | 1.4304 | R(C1-C6) | 1.4173 | 1.4223 | 1.4189 | 1.4277 |
| R(C1-C17) | 1.4174 | 1.439 | 1.4287 | 1.446 | R(C1-C17) | 1.4158 | 1.4386 | 1.428 | 1.4457 | R(C1-C17) | 1.4139 | 1.4355 | 1.4252 | 1.4424 |
| R(C2-C3) | 1.4394 | 1.4373 | 1.438 | 1.4403 | R(C2-C3) | 1.4383 | 1.437 | 1.4374 | 1.4402 | R(C2-C3) | 1.438 | 1.4358 | 1.4364 | 1.439 |
| R(C2-C11) | 1.3954 | 1.4181 | 1.407 | 1.4262 | R(C2-C11) | 1.3953 | 1.4207 | 1.4091 | 1.4297 | R(C2-C11) | 1.3946 | 1.418 | 1.4067 | 1.4265 |
| R(C3-C4) | 1.4177 | 1.4225 | 1.4192 | 1.4276 | R(C3-C4) | 1.419 | 1.4246 | 1.4211 | 1.4304 | R(C3-C4) | 1.4169 | 1.4222 | 1.4186 | 1.4276 |
| R(C3-C14) | 1.3954 | 1.4181 | 1.407 | 1.4262 | R(C3-C14) | 1.3953 | 1.4207 | 1.4091 | 1.4297 | R(C3-C14) | 1.3946 | 1.418 | 1.4067 | 1.4266 |
| R(C4-C5) | 1.4177 | 1.4225 | 1.4192 | 1.4276 | R(C4-C5) | 1.419 | 1.4246 | 1.4211 | 1.4304 | R(C4-C5) | 1.4173 | 1.4223 | 1.4189 | 1.4277 |
| R(C4-C36) | 1.4174 | 1.439 | 1.4287 | 1.446 | R(C4-C36) | 1.4158 | 1.4386 | 1.428 | 1.4457 | R(C4-C36) | 1.4141 | 1.4356 | 1.4253 | 1.4424 |
| R(C5-C6) | 1.4394 | 1.4373 | 1.438 | 1.4403 | R(C5-C6) | 1.4383 | 1.437 | 1.4374 | 1.4402 | R(C5-C6) | 1.4396 | 1.4374 | 1.4381 | 1.4404 |
| R(C5-C8) | 1.3954 | 1.4181 | 1.407 | 1.4262 | R(C5-C8) | 1.3953 | 1.4207 | 1.4091 | 1.4297 | R(C5-C8) | 1.3939 | 1.4185 | 1.4069 | 1.4273 |
| R(C6-C7) | 1.3954 | 1.4181 | 1.407 | 1.4262 | R(C6-C7) | 1.3953 | 1.4207 | 1.4091 | 1.4297 | R(C6-C7) | 1.3939 | 1.4186 | 1.4069 | 1.4273 |
| R(C7-C10) | 1.4304 | 1.4267 | 1.4282 | 1.4299 | R(C7-C10) | 1.4315 | 1.4276 | 1.4293 | 1.4313 | R(C7-C10) | 1.4312 | 1.4273 | 1.4289 | 1.4308 |
| R(C7-C19) | 1.4317 | 1.4404 | 1.4347 | 1.4459 | R(C7-C19) | 1.432 | 1.4411 | 1.4352 | 1.4468 | R(C7-C19) | 1.4372 | 1.4454 | 1.4398 | 1.4509 |
| R(C8-C9) | 1.4304 | 1.4267 | 1.4282 | 1.4299 | R(C8-C9) | 1.4315 | 1.4276 | 1.4293 | 1.4313 | R(C8-C9) | 1.4312 | 1.4274 | 1.429 | 1.4308 |
| R(C8-C38) | 1.4317 | 1.4404 | 1.4347 | 1.4459 | R(C8-C38) | 1.432 | 1.4411 | 1.4352 | 1.4468 | R(C8-C38) | 1.4373 | 1.4454 | 1.4398 | 1.4508 |
| R(C9-C10) | 1.4135 | 1.4291 | 1.4221 | 1.4351 | R(C9-C10) | 1.4115 | 1.4293 | 1.4219 | 1.4357 | R(C9-C10) | 1.4152 | 1.4303 | 1.4236 | 1.436 |
| R(C9-C23) | 1.41 | 1.4271 | 1.4173 | 1.435 | R(C9-C23) | 1.414 | 1.4314 | 1.4214 | 1.44 | R(C9-C23) | 1.4091 | 1.4274 | 1.4174 | 1.4356 |
| R(C10-C21) | 1.41 | 1.4271 | 1.4173 | 1.435 | R(C10-C21) | 1.414 | 1.4314 | 1.4214 | 1.44 | R(C10-C21) | 1.4092 | 1.4275 | 1.4174 | 1.4356 |
| R(C11-C12) | 1.4304 | 1.4267 | 1.4282 | 1.4299 | R(C11-C12) | 1.4315 | 1.4276 | 1.4293 | 1.4313 | R(C11-C12) | 1.4301 | 1.4263 | 1.4276 | 1.4294 |
| R(C11-C16) | 1.4317 | 1.4404 | 1.4347 | 1.4459 | R(C11-C16) | 1.432 | 1.4411 | 1.4352 | 1.4468 | R(C11-C16) | 1.4299 | 1.4387 | 1.4329 | 1.4441 |
| R(C12-C13) | 1.4135 | 1.4291 | 1.4221 | 1.4351 | R(C12-C13) | 1.4115 | 1.4293 | 1.4219 | 1.4357 | R(C12-C13) | 1.4127 | 1.4276 | 1.4208 | 1.4331 |
| R(C12-C28) | 1.41 | 1.4271 | 1.4173 | 1.435 | R(C12-C28) | 1.4139 | 1.4314 | 1.4214 | 1.44 | R(C12-C28) | 1.409 | 1.4272 | 1.4172 | 1.4354 |
| R(C13-C14) | 1.4304 | 1.4267 | 1.4282 | 1.4299 | R(C13-C14) | 1.4315 | 1.4276 | 1.4293 | 1.4313 | R(C13-C14) | 1.4301 | 1.4262 | 1.4276 | 1.4294 |
| R(C13-C31) | 1.41 | 1.4271 | 1.4173 | 1.435 | R(C13-C31) | 1.4139 | 1.4314 | 1.4214 | 1.44 | R(C13-C31) | 1.409 | 1.4273 | 1.4172 | 1.4354 |
| R(C14-C34) | 1.4317 | 1.4404 | 1.4347 | 1.4459 | R(C14-C34) | 1.432 | 1.4411 | 1.4352 | 1.4468 | R(C14-C34) | 1.43 | 1.4387 | 1.433 | 1.4441 |
| R(C15-C17) | 1.4139 | 1.4157 | 1.414 | 1.4202 | R(C15-C17) | 1.4134 | 1.4148 | 1.4137 | 1.4196 | R(C19-C20) | 1.4503 | 1.4399 | 1.4444 | 1.4416 |
| R(C16-C26) | 1.4513 | 1.441 | 1.4446 | 1.4418 | R(C16-C26) | 1.4472 | 1.4354 | 1.4397 | 1.4361 | R(C15-C17) | 1.4123 | 1.4134 | 1.4117 | 1.4176 |
| R(C17-C18) | 1.4139 | 1.4157 | 1.414 | 1.4202 | R(C17-C18) | 1.4134 | 1.4148 | 1.4137 | 1.4196 | R(C16-C26) | 1.4465 | 1.4373 | 1.4416 | 1.4393 |
| R(C19-C20) | 1.4513 | 1.441 | 1.4446 | 1.4418 | R(C19-C20) | 1.4472 | 1.4354 | 1.4397 | 1.4361 | R(C17-C18) | 1.4106 | 1.4136 | 1.4125 | 1.4193 |
| R(C21-C22) | 1.45 | 1.4388 | 1.4432 | 1.4395 | R(C21-C22) | 1.4436 | 1.4309 | 1.4359 | 1.4316 | R(C21-C22) | 1.4466 | 1.4338 | 1.4381 | 1.4335 |
| R(C23-C40) | 1.45 | 1.4388 | 1.4432 | 1.4395 | R(C23-C40) | 1.4436 | 1.4309 | 1.4359 | 1.4316 | R(C21-C25) | 1.3938 | 1.4106 | 1.4041 | 1.4181 |
| R(C27-C28) | 1.45 | 1.4388 | 1.4432 | 1.4395 | R(C27-C28) | 1.4436 | 1.4309 | 1.4359 | 1.4316 | R(C23-C24) | 1.4106 | 1.4135 | 1.4124 | 1.4193 |
| R(C31-C32) | 1.45 | 1.4388 | 1.4432 | 1.4395 | R(C31-C32) | 1.4436 | 1.4309 | 1.4359 | 1.4316 | R(C23-C40) | 1.4466 | 1.4338 | 1.4382 | 1.4336 |
| R(C33-C34) | 1.4513 | 1.441 | 1.4446 | 1.4418 | R(C33-C34) | 1.4472 | 1.4354 | 1.4397 | 1.4361 | R(C27-C28) | 1.4494 | 1.4373 | 1.4415 | 1.4372 |
| R(C35-C36) | 1.4139 | 1.4157 | 1.414 | 1.4202 | R(C35-C36) | 1.4134 | 1.4148 | 1.4137 | 1.4196 | R(C31-C32) | 1.4495 | 1.4374 | 1.4415 | 1.4373 |
| R(C36-C37) | 1.4139 | 1.4157 | 1.414 | 1.4202 | R(C36-C37) | 1.4134 | 1.4148 | 1.4137 | 1.4196 | R(C35-C36) | 1.4123 | 1.4135 | 1.4117 | 1.4176 |
| R(C38-C39) | 1.4513 | 1.441 | 1.4446 | 1.4418 | R(C38-C39) | 1.4472 | 1.4354 | 1.4397 | 1.4361 | R(C38-C39) | 1.4503 | 1.4399 | 1.4445 | 1.4416 |
| R(C15=C16) | 1.3734 | 1.3942 | 1.3852 | 1.4031 | R(C15=C16) | 1.369 | 1.3931 | 1.3839 | 1.4031 | R(C36=C37) | 1.3938 | 1.4106 | 1.4041 | 1.4181 |
| R(C20=C22) | 1.3407 | 1.3644 | 1.3547 | 1.3747 | R(C18=C19) | 1.369 | 1.3931 | 1.3839 | 1.4031 | R(C26=C27) | 1.3331 | 1.359 | 1.3495 | 1.3704 |
| R(C21=C25) | 1.3953 | 1.4113 | 1.405 | 1.4185 | R(C20=C22) | 1.3318 | 1.3637 | 1.353 | 1.3769 | R(C28=C29) | 1.3949 | 1.4111 | 1.4048 | 1.4186 |
| R(C23=C24) | 1.3953 | 1.4113 | 1.405 | 1.4185 | R(C21=C25) | 1.3854 | 1.4051 | 1.3985 | 1.4132 | R(C29=C30) | 1.3826 | 1.3878 | 1.3845 | 1.3931 |
| R(C24=C25) | 1.3826 | 1.3879 | 1.3848 | 1.3935 | R(C23=C24) | 1.3854 | 1.4052 | 1.3985 | 1.4132 | R(C30=C31) | 1.3949 | 1.4111 | 1.4048 | 1.4185 |
| R(C26=C27) | 1.3407 | 1.3644 | 1.3547 | 1.3747 | R(C24=C25) | 1.3792 | 1.3873 | 1.3847 | 1.3951 | R(C24=C25) | 1.3815 | 1.386 | 1.3829 | 1.3912 |
| R(C32=C33) | 1.3407 | 1.3644 | 1.3547 | 1.3747 | R(C26=C27) | 1.3318 | 1.3637 | 1.353 | 1.3769 | R(C32=C33) | 1.333 | 1.359 | 1.3494 | 1.3704 |
| R(C28=C29) | 1.3953 | 1.4113 | 1.405 | 1.4185 | R(C28=C29) | 1.3854 | 1.4051 | 1.3985 | 1.4132 | R(C33=C34) | 1.4465 | 1.4373 | 1.4416 | 1.4394 |
| R(C29=C30) | 1.3826 | 1.3879 | 1.3848 | 1.3935 | R(C29=C30) | 1.3792 | 1.3873 | 1.3847 | 1.3951 | R(C34=C35) | 1.3713 | 1.3917 | 1.3827 | 1.4004 |
| R(C30=C31) | 1.3953 | 1.4113 | 1.405 | 1.4185 | R(C30=C31) | 1.3854 | 1.4052 | 1.3985 | 1.4132 | R(C37=C38) | 1.3691 | 1.3927 | 1.3836 | 1.4027 |
| R(C18=C19) | 1.3734 | 1.3942 | 1.3852 | 1.4031 | R(C32=C33) | 1.3318 | 1.3637 | 1.353 | 1.3769 | R(C39=C40) | 1.3325 | 1.359 | 1.3492 | 1.3705 |
| R(C34=C35) | 1.3734 | 1.3942 | 1.3852 | 1.4031 | R(C34=C35) | 1.369 | 1.3931 | 1.3839 | 1.403 | R(C15=C16) | 1.3713 | 1.3918 | 1.3828 | 1.4005 |
| R(C37=C38) | 1.3734 | 1.3942 | 1.3852 | 1.4031 | R(C37=C38) | 1.369 | 1.3931 | 1.3839 | 1.403 | R(C18=C19) | 1.3692 | 1.3926 | 1.3836 | 1.4027 |
| R(C39=C40) | 1.3407 | 1.3644 | 1.3547 | 1.3747 | R(C39=C40) | 1.3318 | 1.3637 | 1.353 | 1.3769 | R(C20=C22) | 1.3325 | 1.3591 | 1.3492 | 1.3705 |
| R(C15-H41) | 1.0821 | 1.0933 | 1.0928 | 1.1002 | R(C30-F41) | 1.3137 | 1.3375 | 1.3289 | 1.345 | R(C15-H45) | 1.0772 | 1.0882 | 1.0882 | 1.0951 |
| R(C18-H42) | 1.0821 | 1.0933 | 1.0928 | 1.1002 | R(C29-F42) | 1.3137 | 1.3375 | 1.3289 | 1.345 | R(C22-H49) | 1.0801 | 1.091 | 1.0903 | 1.0977 |
| R(C20-H43) | 1.0818 | 1.0928 | 1.092 | 1.0996 | R(C27-F43) | 1.3163 | 1.3387 | 1.3305 | 1.3459 | R(C24-H52) | 1.0815 | 1.0923 | 1.0916 | 1.0991 |
| R(C22-H44) | 1.0821 | 1.093 | 1.0922 | 1.0998 | R(C26-F44) | 1.3148 | 1.3378 | 1.3297 | 1.3453 | R(C25-H46) | 1.0815 | 1.0923 | 1.0916 | 1.0991 |
| R(C24-H45) | 1.082 | 1.0929 | 1.0921 | 1.0997 | R(C15-F45) | 1.3131 | 1.3386 | 1.3294 | 1.3463 | R(C27-H44) | 1.0802 | 1.091 | 1.0904 | 1.0978 |
| R(C25-H46) | 1.082 | 1.0929 | 1.0921 | 1.0997 | R(C18-F46) | 1.3131 | 1.3386 | 1.3294 | 1.3463 | R(C32-H48) | 1.0802 | 1.091 | 1.0904 | 1.0978 |
| R(C26-H47) | 1.0818 | 1.0928 | 1.092 | 1.0996 | R(C20-F47) | 1.3148 | 1.3378 | 1.3297 | 1.3453 | R(C40-H47) | 1.0801 | 1.091 | 1.0903 | 1.0977 |
| R(C27-H48) | 1.0821 | 1.093 | 1.0922 | 1.0998 | R(C22-F48) | 1.3163 | 1.3387 | 1.3305 | 1.3459 | R(C35-H53) | 1.0772 | 1.0882 | 1.0883 | 1.0951 |
| R(C29-H49) | 1.082 | 1.0929 | 1.0921 | 1.0997 | R(C25-F49) | 1.3137 | 1.3375 | 1.3289 | 1.345 | R(C29-H56) | 1.0816 | 1.0924 | 1.0917 | 1.0992 |
| R(C30-H50) | 1.082 | 1.0929 | 1.0921 | 1.0997 | R(C24-F50) | 1.3137 | 1.3375 | 1.3289 | 1.345 | R(C30-H43) | 1.0816 | 1.0924 | 1.0917 | 1.0992 |
| R(C32-H51) | 1.0821 | 1.093 | 1.0922 | 1.0998 | R(C40-F51) | 1.3163 | 1.3386 | 1.3305 | 1.3459 | R(C39-F55) | 1.3233 | 1.3465 | 1.3377 | 1.3538 |
| R(C33-H52) | 1.0818 | 1.0928 | 1.092 | 1.0996 | R(C39-F52) | 1.3148 | 1.3378 | 1.3297 | 1.3453 | R(C33-F50) | 1.3277 | 1.3511 | 1.3419 | 1.358 |
| R(C35-H53) | 1.0821 | 1.0933 | 1.0928 | 1.1002 | R(C37-F53) | 1.3131 | 1.3386 | 1.3294 | 1.3463 | R(C37-F51) | 1.3228 | 1.3471 | 1.3377 | 1.3538 |
| R(C37-H54) | 1.0821 | 1.0933 | 1.0928 | 1.1002 | R(C35-F54) | 1.3131 | 1.3386 | 1.3294 | 1.3463 | R(C26-F41) | 1.3276 | 1.3509 | 1.3418 | 1.358 |
| R(C39-H55) | 1.0818 | 1.0928 | 1.092 | 1.0996 | R(C33-F55) | 1.3148 | 1.3378 | 1.3297 | 1.3453 | R(C18-F42) | 1.3228 | 1.347 | 1.3377 | 1.3538 |
| R(C40-H56) | 1.0821 | 1.093 | 1.0922 | 1.0998 | R(C32-F56) | 1.3163 | 1.3386 | 1.3305 | 1.3459 | R(C20-F54) | 1.3233 | 1.3466 | 1.3377 | 1.3537 |

Table S2: Optimized geometrical parameters of the C_40_H_16_ molecule obtained with the RHF, B3LYP, wB97XD, and BPBE methods by employing cc-pVDZ basis sets. Bond angles are given in degrees (°).

| Bond angles of C_40_H_16_ (°) | | | | |
| --- | --- | --- | --- | --- |
|  | RHF | B3LYP | WB97XD | BPBE |
| A(C2-C1-C6) | 120.2379 | 120.1247 | 120.1699 | 120.1176 |
| A(C2-C1-C17) | 119.881 | 119.9376 | 119.9151 | 119.9412 |
| A(C6-C1-C17) | 119.881 | 119.9376 | 119.9151 | 119.9412 |
| A(C1-C2-C3) | 119.881 | 119.9376 | 119.915 | 119.9412 |
| A(C1-C2-C11) | 120.0365 | 120.0744 | 120.0375 | 120.097 |
| A(C3-C2-C11) | 120.0824 | 119.988 | 120.0474 | 119.9618 |
| A(C2-C3-C4) | 119.881 | 119.9376 | 119.915 | 119.9412 |
| A(C2-C3-C14) | 120.0824 | 119.988 | 120.0473 | 119.9618 |
| A(C4-C3-C14) | 120.0365 | 120.0744 | 120.0377 | 120.097 |
| A(C3-C4-C5) | 120.2379 | 120.1247 | 120.17 | 120.1176 |
| A(C3-C4-C36) | 119.881 | 119.9376 | 119.915 | 119.9412 |
| A(C5-C4-C36) | 119.881 | 119.9376 | 119.915 | 119.9412 |
| A(C4-C5-C6) | 119.881 | 119.9376 | 119.915 | 119.9412 |
| A(C4-C5-C8) | 120.0365 | 120.0744 | 120.0377 | 120.097 |
| A(C6-C5-C8) | 120.0824 | 119.988 | 120.0473 | 119.9618 |
| A(C1-C6-C5) | 119.881 | 119.9376 | 119.915 | 119.9412 |
| A(C1-C6-C7) | 120.0365 | 120.0744 | 120.0375 | 120.097 |
| A(C5-C6-C7) | 120.0824 | 119.988 | 120.0474 | 119.9618 |
| A(C6-C7-C10) | 120.0488 | 120.0346 | 120.029 | 120.04 |
| A(C6-C7-C19) | 119.9286 | 119.8131 | 119.909 | 119.7992 |
| A(C10-C7-C19) | 120.0227 | 120.1524 | 120.0621 | 120.1608 |
| A(C5-C8-C9) | 120.0488 | 120.0346 | 120.0291 | 120.04 |
| A(C5-C8-C38) | 119.9286 | 119.8131 | 119.9089 | 119.7992 |
| A(C9-C8-C38) | 120.0227 | 120.1524 | 120.062 | 120.1608 |
| A(C8-C9-C10) | 119.8688 | 119.9775 | 119.9236 | 119.9982 |
| A(C8-C9-C23) | 120.2126 | 120.1526 | 120.1769 | 120.1225 |
| A(C10-C9-C23) | 119.9186 | 119.87 | 119.8995 | 119.8792 |
| A(C7-C10-C9) | 119.8688 | 119.9775 | 119.9236 | 119.9982 |
| A(C7-C10-C21) | 120.2126 | 120.1526 | 120.1768 | 120.1225 |
| A(C9-C10-C21) | 119.9186 | 119.87 | 119.8996 | 119.8792 |
| A(C2-C11-C12) | 120.0488 | 120.0346 | 120.029 | 120.04 |
| A(C2-C11-C16) | 119.9286 | 119.8131 | 119.909 | 119.7992 |
| A(C12-C11-C16) | 120.0227 | 120.1524 | 120.0621 | 120.1608 |
| A(C11-C12-C13) | 119.8688 | 119.9775 | 119.9236 | 119.9982 |
| A(C11-C12-C28) | 120.2126 | 120.1526 | 120.1768 | 120.1225 |
| A(C13-C12-C28) | 119.9186 | 119.87 | 119.8996 | 119.8792 |
| A(C12-C13-C14) | 119.8688 | 119.9775 | 119.9236 | 119.9982 |
| A(C12-C13-C31) | 119.9186 | 119.87 | 119.8995 | 119.8792 |
| A(C14-C13-C31) | 120.2126 | 120.1526 | 120.1769 | 120.1225 |
| A(C3-C14-C13) | 120.0488 | 120.0346 | 120.0291 | 120.04 |
| A(C3-C14-C34) | 119.9286 | 119.8131 | 119.9089 | 119.7992 |
| A(C13-C14-C34) | 120.0227 | 120.1524 | 120.062 | 120.1608 |
| A(C16-C15-C17) | 121.5697 | 121.9068 | 121.6822 | 121.9413 |
| A(C11-C16-C15) | 119.6592 | 119.458 | 119.5612 | 119.3937 |
| A(C11-C16-C26) | 118.0764 | 118.1531 | 118.2101 | 118.2355 |
| A(C15-C16-C26) | 122.2644 | 122.3889 | 122.2287 | 122.3708 |
| A(C1-C17-C15) | 118.925 | 118.8101 | 118.895 | 118.8276 |
| A(C1-C17-C18) | 118.925 | 118.8101 | 118.895 | 118.8276 |
| A(C15-C17-C18) | 122.1499 | 122.3799 | 122.21 | 122.3448 |
| A(C17-C18-C19) | 121.5697 | 121.9068 | 121.6822 | 121.9413 |
| A(C7-C19-C18) | 119.6592 | 119.458 | 119.5612 | 119.3937 |
| A(C7-C19-C20) | 118.0764 | 118.1531 | 118.2101 | 118.2355 |
| A(C18-C19-C20) | 122.2644 | 122.3889 | 122.2287 | 122.3708 |
| A(C19-C20-C22) | 121.4124 | 121.3944 | 121.3429 | 121.3578 |
| A(C10-C21-C22) | 118.7027 | 118.6336 | 118.7471 | 118.6507 |
| A(C10-C21-C25) | 119.0751 | 118.9161 | 119.0274 | 118.8811 |
| A(C22-C21-C25) | 122.2222 | 122.4502 | 122.2255 | 122.4682 |
| A(C20-C22-C21) | 121.5732 | 121.514 | 121.461 | 121.4726 |
| A(C9-C23-C24) | 119.0751 | 118.9161 | 119.0274 | 118.8811 |
| A(C9-C23-C40) | 118.7027 | 118.6336 | 118.7471 | 118.6507 |
| A(C24-C23-C40) | 122.2222 | 122.4502 | 122.2255 | 122.4682 |
| A(C23-C24-C25) | 121.0063 | 121.2139 | 121.0731 | 121.2397 |
| A(C21-C25-C24) | 121.0063 | 121.2139 | 121.0731 | 121.2397 |
| A(C16-C26-C27) | 121.4124 | 121.3944 | 121.3429 | 121.3578 |
| A(C26-C27-C28) | 121.5732 | 121.514 | 121.461 | 121.4726 |
| A(C12-C28-C27) | 118.7027 | 118.6337 | 118.7471 | 118.6507 |
| A(C12-C28-C29) | 119.0751 | 118.9161 | 119.0274 | 118.8811 |
| A(C27-C28-C29) | 122.2222 | 122.4502 | 122.2255 | 122.4682 |
| A(C28-C29-C30) | 121.0063 | 121.2139 | 121.0731 | 121.2397 |
| A(C29-C30-C31) | 121.0063 | 121.2139 | 121.0731 | 121.2397 |
| A(C13-C31-C30) | 119.0751 | 118.9161 | 119.0274 | 118.8811 |
| A(C13-C31-C32) | 118.7027 | 118.6336 | 118.7471 | 118.6507 |
| A(C30-C31-C32) | 122.2222 | 122.4502 | 122.2255 | 122.4682 |
| A(C31-C32-C33) | 121.5732 | 121.514 | 121.461 | 121.4726 |
| A(C32-C33-C34) | 121.4124 | 121.3944 | 121.3429 | 121.3578 |
| A(C14-C34-C33) | 118.0764 | 118.1531 | 118.2102 | 118.2355 |
| A(C14-C34-C35) | 119.6592 | 119.458 | 119.5611 | 119.3937 |
| A(C33-C34-C35) | 122.2644 | 122.3889 | 122.2286 | 122.3708 |
| A(C34-C35-C36) | 121.5697 | 121.9068 | 121.6822 | 121.9413 |
| A(C4-C36-C35) | 118.925 | 118.8101 | 118.895 | 118.8276 |
| A(C4-C36-C37) | 118.925 | 118.8101 | 118.895 | 118.8276 |
| A(C35-C36-C37) | 122.1499 | 122.3799 | 122.21 | 122.3448 |
| A(C36-C37-C38) | 121.5697 | 121.9068 | 121.6822 | 121.9413 |
| A(C8-C38-C37) | 119.6592 | 119.458 | 119.5611 | 119.3937 |
| A(C8-C38-C39) | 118.0764 | 118.1531 | 118.2102 | 118.2355 |
| A(C37-C38-C39) | 122.2644 | 122.3889 | 122.2286 | 122.3708 |
| A(C38-C39-C40) | 121.4124 | 121.3944 | 121.3429 | 121.3578 |
| A(C23-C40-C39) | 121.5732 | 121.514 | 121.461 | 121.4726 |
| A(C16-C15-H41) | 119.5869 | 119.2649 | 119.4384 | 119.2065 |
| A(C17-C15-H41) | 118.8434 | 118.8283 | 118.8794 | 118.8522 |
| A(C17-C18-H42) | 118.8434 | 118.8283 | 118.8794 | 118.8522 |
| A(C19-C18-H42) | 119.5869 | 119.2649 | 119.4384 | 119.2065 |
| A(C19-C20-H43) | 118.1615 | 118.2576 | 118.214 | 118.3624 |
| A(C22-C20-H43) | 120.4261 | 120.3481 | 120.4431 | 120.2798 |
| A(C20-C22-H44) | 120.2705 | 120.2087 | 120.3219 | 120.1444 |
| A(C21-C22-H44) | 118.1563 | 118.2773 | 118.2171 | 118.3829 |
| A(C23-C24-H45) | 119.3013 | 118.8877 | 119.0467 | 118.8526 |
| A(C25-C24-H45) | 119.6924 | 119.8984 | 119.8802 | 119.9077 |
| A(C21-C25-H46) | 119.3013 | 118.8877 | 119.0467 | 118.8526 |
| A(C24-C25-H46) | 119.6924 | 119.8984 | 119.8803 | 119.9077 |
| A(C16-C26-H47) | 118.1615 | 118.2576 | 118.214 | 118.3624 |
| A(C27-C26-H47) | 120.4261 | 120.3481 | 120.4431 | 120.2798 |
| A(C26-C27-H48) | 120.2705 | 120.2087 | 120.3219 | 120.1444 |
| A(C28-C27-H48) | 118.1563 | 118.2773 | 118.2171 | 118.3829 |
| A(C28-C29-H49) | 119.3013 | 118.8877 | 119.0467 | 118.8526 |
| A(C30-C29-H49) | 119.6924 | 119.8984 | 119.8803 | 119.9077 |
| A(C29-C30-H50) | 119.6924 | 119.8984 | 119.8802 | 119.9077 |
| A(C31-C30-H50) | 119.3013 | 118.8877 | 119.0467 | 118.8526 |
| A(C31-C32-H51) | 118.1563 | 118.2773 | 118.217 | 118.3829 |
| A(C33-C32-H51) | 120.2705 | 120.2087 | 120.322 | 120.1444 |
| A(C32-C33-H52) | 120.4261 | 120.3481 | 120.443 | 120.2798 |
| A(C34-C33-C52) | 118.1615 | 118.2576 | 118.2142 | 118.3624 |
| A(C34-C35-H53) | 119.5869 | 119.2649 | 119.4382 | 119.2065 |
| A(C36-C35-H53) | 118.8434 | 118.8283 | 118.8795 | 118.8522 |
| A(C36-C37-H54) | 118.8434 | 118.8283 | 118.8795 | 118.8522 |
| A(C38-C37-H54) | 119.5869 | 119.2649 | 119.4382 | 119.2065 |
| A(C38-C39-H55) | 118.1615 | 118.2576 | 118.2142 | 118.3624 |
| A(C40-C39-H55) | 120.4261 | 120.3481 | 120.443 | 120.2798 |
| A(C23-C40-H56) | 118.1563 | 118.2773 | 118.217 | 118.3829 |
| A(C39-C40-H56) | 120.2705 | 120.2087 | 120.322 | 120.1444 |

Table S3: Optimized geometrical parameters of the C_40_F16, and C_40_H_10_F_6_ molecules obtained with the RHF, B3LYP, wB97XD, and BPBE methods by employing cc-pVDZ basis sets. Bond angles are given in degrees (°).

| Bond angles of C_40_F16 (°) | | | | | Bond angles of C_40_H_10_F_6_ (°) | | | | |
| --- | --- | --- | --- | --- | --- | --- | --- | --- | --- |
|  | RHF | B3LYP | WB97XD | BPBE |  | RHF | B3LYP | WB97XD | BPBE |
| A(C2-C1-C6) | 120.2614 | 120.3617 | 120.4116 | 120.4488 | A(C2-C1-C6) | 120.4427 | 120.4153 | 120.4572 | 120.444 |
| A(C2-C1-C17) | 119.8693 | 119.8192 | 119.7942 | 119.7756 | A(C2-C1-C17) | 119.3514 | 119.3091 | 119.2837 | 119.2638 |
| A(C6-C1-C17) | 119.8693 | 119.8192 | 119.7942 | 119.7756 | A(C6-C1-C17) | 120.2059 | 120.2756 | 120.2591 | 120.2921 |
| A(C1-C2-C3) | 119.8693 | 119.8192 | 119.7942 | 119.7756 | A(C1-C2-C3) | 119.8029 | 119.8073 | 119.7952 | 119.7978 |
| A(C1-C2-C11) | 120.0055 | 120.2614 | 120.1997 | 120.3679 | A(C1-C2-C11) | 120.1716 | 120.2989 | 120.2636 | 120.3549 |
| A(C3-C2-C11) | 120.1252 | 119.9194 | 120.0061 | 119.8564 | A(C3-C2-C11) | 120.0256 | 119.8938 | 119.9413 | 119.8473 |
| A(C2-C3-C4) | 119.8694 | 119.819 | 119.794 | 119.7756 | A(C2-C3-C4) | 119.8006 | 119.8215 | 119.7891 | 119.7924 |
| A(C2-C3-C14) | 120.1254 | 119.9193 | 120.006 | 119.8567 | A(C2-C3-C14) | 120.0242 | 119.8787 | 119.9473 | 119.8551 |
| A(C4-C3-C14) | 120.0053 | 120.2616 | 120.2 | 120.3678 | A(C4-C3-C14) | 120.1752 | 120.2998 | 120.2636 | 120.3525 |
| A(C3-C4-C5) | 120.2613 | 120.3619 | 120.4119 | 120.4487 | A(C3-C4-C5) | 120.4458 | 120.408 | 120.4604 | 120.4437 |
| A(C3-C4-C36) | 119.8694 | 119.819 | 119.794 | 119.7756 | A(C3-C4-C36) | 119.3497 | 119.3196 | 119.2791 | 119.2608 |
| A(C5-C4-C36) | 119.8693 | 119.819 | 119.794 | 119.7756 | A(C5-C4-C36) | 120.2045 | 120.2724 | 120.2605 | 120.2954 |
| A(C4-C5-C6) | 119.8693 | 119.819 | 119.794 | 119.7756 | A(C4-C5-C6) | 119.7531 | 119.7712 | 119.7491 | 119.763 |
| A(C4-C5-C8) | 120.0053 | 120.2616 | 120.2 | 120.3678 | A(C4-C5-C8) | 119.8927 | 120.0379 | 119.9722 | 120.0626 |
| A(C6-C5-C8) | 120.1254 | 119.9193 | 120.006 | 119.8567 | A(C6-C5-C8) | 120.3542 | 120.191 | 120.2787 | 120.1744 |
| A(C1-C6-C5) | 119.8693 | 119.8192 | 119.7942 | 119.7756 | A(C1-C6-C5) | 119.7549 | 119.7768 | 119.7491 | 119.759 |
| A(C1-C6-C7) | 120.0055 | 120.2614 | 120.1997 | 120.3679 | A(C1-C6-C7) | 119.8899 | 120.0384 | 119.9719 | 120.0664 |
| A(C5-C6-C7) | 120.1252 | 119.9194 | 120.0061 | 119.8564 | A(C5-C6-C7) | 120.3552 | 120.1848 | 120.279 | 120.1746 |
| A(C6-C7-C10) | 119.9697 | 120.144 | 120.097 | 120.2188 | A(C6-C7-C10) | 119.5981 | 119.6679 | 119.6228 | 119.6329 |
| A(C6-C7-C19) | 120.1554 | 119.8243 | 119.9857 | 119.7491 | A(C6-C7-C19) | 120.2833 | 120.0533 | 120.2134 | 120.0742 |
| A(C10-C7-C19) | 119.875 | 120.0317 | 119.9173 | 120.0321 | A(C10-C7-C19) | 120.1186 | 120.2788 | 120.1638 | 120.2929 |
| A(C5-C8-C9) | 119.9694 | 120.1443 | 120.0973 | 120.2187 | A(C5-C8-C9) | 119.5996 | 119.6658 | 119.6206 | 119.6286 |
| A(C5-C8-C38) | 120.1555 | 119.8242 | 119.9856 | 119.7491 | A(C5-C8-C38) | 120.2825 | 120.0561 | 120.2121 | 120.0739 |
| A(C9-C8-C38) | 119.8751 | 120.0315 | 119.9171 | 120.0322 | A(C9-C8-C38) | 120.1179 | 120.2782 | 120.1673 | 120.2974 |
| A(C8-C9-C10) | 119.9053 | 119.9364 | 119.8967 | 119.9246 | A(C8-C9-C10) | 120.0454 | 120.143 | 120.1004 | 120.1967 |
| A(C8-C9-C23) | 120.1113 | 120.1557 | 120.1541 | 120.1602 | A(C8-C9-C23) | 120.1541 | 120.1554 | 120.1676 | 120.1278 |
| A(C10-C9-C23) | 119.9834 | 119.9079 | 119.9492 | 119.9152 | A(C10-C9-C23) | 119.8006 | 119.7016 | 119.732 | 119.6755 |
| A(C7-C10-C9) | 119.9051 | 119.9366 | 119.897 | 119.9249 | A(C7-C10-C9) | 120.0475 | 120.1476 | 120.0985 | 120.1928 |
| A(C7-C10-C21) | 120.1115 | 120.1556 | 120.1538 | 120.1602 | A(C7-C10-C21) | 120.1517 | 120.1564 | 120.1656 | 120.1288 |
| A(C9-C10-C21) | 119.9835 | 119.9079 | 119.9492 | 119.9149 | A(C9-C10-C21) | 119.8008 | 119.6959 | 119.7359 | 119.6785 |
| A(C2-C11-C12) | 119.9697 | 120.144 | 120.097 | 120.2188 | A(C2-C11-C12) | 120.1854 | 120.2264 | 120.2336 | 120.2291 |
| A(C2-C11-C16) | 120.1554 | 119.8243 | 119.9857 | 119.7491 | A(C2-C11-C16) | 119.5552 | 119.3152 | 119.421 | 119.2724 |
| A(C12-C11-C16) | 119.875 | 120.0317 | 119.9173 | 120.0321 | A(C12-C11-C16) | 120.2594 | 120.4584 | 120.3455 | 120.4986 |
| A(C11-C12-C13) | 119.9051 | 119.9365 | 119.8969 | 119.9249 | A(C11-C12-C13) | 119.7896 | 119.8811 | 119.8247 | 119.9237 |
| A(C11-C12-C28) | 120.1115 | 120.1556 | 120.1538 | 120.1602 | A(C11-C12-C28) | 120.1633 | 120.1531 | 120.171 | 120.1121 |
| A(C13-C12-C28) | 119.9835 | 119.9079 | 119.9492 | 119.9149 | A(C13-C12-C28) | 120.047 | 119.9658 | 120.0042 | 119.9643 |
| A(C12-C13-C14) | 119.9053 | 119.9364 | 119.8967 | 119.9246 | A(C12-C13-C14) | 119.7876 | 119.8894 | 119.8216 | 119.9188 |
| A(C12-C13-C31) | 119.9834 | 119.9079 | 119.9492 | 119.9152 | A(C12-C13-C31) | 120.0467 | 119.957 | 120.0076 | 119.9699 |
| A(C14-C13-C31) | 120.1113 | 120.1557 | 120.1541 | 120.1602 | A(C14-C13-C31) | 120.1656 | 120.1536 | 120.1708 | 120.1113 |
| A(C3-C14-C13) | 119.9694 | 120.1443 | 120.0973 | 120.2187 | A(C3-C14-C13) | 120.1876 | 120.2305 | 120.2316 | 120.226 |
| A(C3-C14-C34) | 120.1555 | 119.8242 | 119.9856 | 119.7491 | A(C3-C14-C34) | 119.5535 | 119.3024 | 119.424 | 119.2768 |
| A(C13-C14-C34) | 119.8751 | 120.0315 | 119.9171 | 120.0322 | A(C13-C14-C34) | 120.259 | 120.4672 | 120.3445 | 120.4972 |
| A(C16-C15-C17) | 122.3785 | 122.7726 | 122.5232 | 122.8431 | A(C16-C15-C17) | 120.2737 | 120.5674 | 120.3483 | 120.6322 |
| A(C11-C16-C15) | 119.0537 | 118.8869 | 118.9574 | 118.8119 | A(C11-C16-C15) | 120.591 | 120.4767 | 120.5723 | 120.4043 |
| A(C11-C16-C26) | 117.9255 | 118.0579 | 118.1475 | 118.1792 | A(C11-C16-C26) | 116.9177 | 116.8949 | 116.9876 | 116.9553 |
| A(C15-C16-C26) | 123.0208 | 123.0552 | 122.8952 | 123.0089 | A(C15-C16-C26) | 122.4913 | 122.6284 | 122.4401 | 122.6404 |
| A(C1-C17-C15) | 118.5376 | 118.4356 | 118.5399 | 118.4524 | A(C1-C17-C15) | 120.0572 | 120.0327 | 120.1111 | 120.0724 |
| A(C1-C17-C18) | 118.5376 | 118.4356 | 118.5399 | 118.4524 | A(C1-C17-C18) | 118.1716 | 117.9997 | 118.1035 | 118.0085 |
| A(C15-C17-C18) | 122.9248 | 123.1287 | 122.9203 | 123.0952 | A(C15-C17-C18) | 121.7713 | 121.9675 | 121.7853 | 121.919 |
| A(C17-C18-C19) | 122.3785 | 122.7726 | 122.5232 | 122.8431 | A(C17-C18-C19) | 122.9704 | 123.3226 | 123.0617 | 123.3533 |
| A(C7-C19-C18) | 119.0537 | 118.8869 | 118.9574 | 118.8119 | A(C7-C19-C18) | 118.4789 | 118.3103 | 118.3903 | 118.2054 |
| A(C7-C19-C20) | 117.9255 | 118.0579 | 118.1474 | 118.1792 | A(C7-C19-C20) | 117.0349 | 117.0883 | 117.165 | 117.1555 |
| A(C18-C19-C20) | 123.0208 | 123.0552 | 122.8952 | 123.0089 | A(C18-C19-C20) | 124.4862 | 124.6015 | 124.4447 | 124.6391 |
| A(C19-C20-C22) | 121.7482 | 121.6076 | 121.5521 | 121.5398 | A(C19-C20-C22) | 122.8108 | 122.7594 | 122.6912 | 122.6937 |
| A(C10-C21-C22) | 118.5319 | 118.4406 | 118.6056 | 118.4567 | A(C10-C21-C22) | 119.0298 | 118.8832 | 119.0669 | 118.9312 |
| A(C10-C21-C25) | 118.5723 | 118.5679 | 118.6718 | 118.5813 | A(C10-C21-C25) | 119.2312 | 119.1636 | 119.2574 | 119.1895 |
| A(C22-C21-C25) | 122.8958 | 122.9915 | 122.7225 | 122.962 | A(C22-C21-C25) | 121.739 | 121.9532 | 121.6757 | 121.8793 |
| A(C20-C22-C21) | 121.808 | 121.7067 | 121.6237 | 121.6321 | A(C20-C22-C21) | 120.8541 | 120.8339 | 120.7476 | 120.7979 |
| A(C13-C31-C30) | 118.5725 | 118.5678 | 118.6716 | 118.5817 | A(C96-C23-C24) | 119.2325 | 119.1676 | 119.2591 | 119.1887 |
| A(C13-C31-C32) | 118.5322 | 118.4405 | 118.6053 | 118.4569 | A(C9-C23-C40) | 119.0292 | 118.8904 | 119.065 | 118.9286 |
| A(C9-C23-C24) | 118.5725 | 118.5678 | 118.6716 | 118.5817 | A(C24-C23-C40) | 121.7382 | 121.942 | 121.6759 | 121.8827 |
| A(C9-C23-C40) | 118.5321 | 118.4405 | 118.6053 | 118.4569 | A(C23-C24-C25) | 120.9669 | 121.1318 | 121.0088 | 121.1362 |
| A(C24-C23-C40) | 122.8954 | 122.9918 | 122.7232 | 122.9615 | A(C21-C25-C24) | 120.968 | 121.1394 | 121.0068 | 121.1317 |
| A(C23-C24-C25) | 121.4441 | 121.5243 | 121.3792 | 121.5032 | A(C16-C26-C27) | 123.3819 | 123.3631 | 123.2765 | 123.2775 |
| A(C21-C25-C24) | 121.4442 | 121.5242 | 121.379 | 121.5036 | A(C26-C27-C28) | 120.2129 | 120.1884 | 120.109 | 120.154 |
| A(C16-C26-C27) | 121.7482 | 121.6076 | 121.5521 | 121.5398 | A(C12-C28-C27) | 119.0648 | 118.9421 | 119.1104 | 119.0025 |
| A(C26-C27-C28) | 121.8079 | 121.7067 | 121.6237 | 121.6321 | A(C12-C28-C29) | 118.8514 | 118.7568 | 118.8353 | 118.7329 |
| A(C12-C28-C27) | 118.5319 | 118.4406 | 118.6056 | 118.4567 | A(C27-C28-C29) | 122.0838 | 122.3012 | 122.0543 | 122.2646 |
| A(C12-C28-C29) | 118.5723 | 118.5679 | 118.6718 | 118.5813 | A(C28-C29-C30) | 121.1017 | 121.2787 | 121.1598 | 121.3026 |
| A(C27-C28-C29) | 122.8958 | 122.9915 | 122.7225 | 122.962 | A(C29-C30-C31) | 121.1003 | 121.285 | 121.1575 | 121.2967 |
| A(C28-C29-C30) | 121.4442 | 121.5242 | 121.379 | 121.5036 | A(C13-C31-C30) | 118.8528 | 118.7567 | 118.8355 | 118.7336 |
| A(C29-C30-C31) | 121.4441 | 121.5243 | 121.3792 | 121.5032 | A(C13-C31-C32) | 119.0643 | 118.9324 | 119.1138 | 119.0075 |
| A(C30-C31-C32) | 122.8954 | 122.9918 | 122.7231 | 122.9614 | A(C30-C31-C32) | 122.0829 | 122.3109 | 122.0506 | 122.2589 |
| A(C31-C32-C33) | 121.8079 | 121.7067 | 121.6238 | 121.632 | A(C31-C32-C33) | 120.2098 | 120.198 | 120.1061 | 120.1488 |
| A(C32-C33-C34) | 121.7481 | 121.6075 | 121.5521 | 121.5398 | A(C32-C33-C34) | 123.3892 | 123.3589 | 123.28 | 123.2799 |
| A(C14-C34-C33) | 117.9255 | 118.0581 | 118.1476 | 118.1789 | A(C14-C34-C33) | 116.9121 | 116.8899 | 116.9848 | 116.9553 |
| A(C14-C34-C35) | 119.0537 | 118.8869 | 118.9572 | 118.8121 | A(C14-C34-C35) | 120.5883 | 120.4929 | 120.5673 | 120.4008 |
| A(C33-C34-C35) | 123.0209 | 123.0551 | 122.8952 | 123.009 | A(C33-C34-C35) | 122.4996 | 122.6172 | 122.448 | 122.6439 |
| A(C34-C35-C36) | 122.3783 | 122.7726 | 122.5232 | 122.8433 | A(C34-C35-C36) | 120.2804 | 120.573 | 120.3524 | 120.6337 |
| A(C4-C36-C35) | 118.5378 | 118.4357 | 118.54 | 118.4521 | A(C4-C36-C35) | 120.0529 | 120.0124 | 120.1136 | 120.0754 |
| A(C4-C36-C37) | 118.5378 | 118.4357 | 118.54 | 118.4521 | A(C4-C36-C37) | 118.1657 | 118.0028 | 118.0973 | 118.0053 |
| A(C35-C36-C37) | 122.9244 | 123.1286 | 122.9201 | 123.0958 | A(C35-C36-C37) | 121.7813 | 121.9847 | 121.789 | 121.9193 |
| A(C36-C37-C38) | 122.3783 | 122.7726 | 122.5232 | 122.8433 | A(C36-C37-C38) | 122.9785 | 123.3152 | 123.0704 | 123.3545 |
| A(C8-C38-C37) | 119.0537 | 118.8869 | 118.9572 | 118.8121 | A(C8-C38-C37) | 118.4761 | 118.3156 | 118.3874 | 118.2083 |
| A(C8-C38-C39) | 117.9254 | 118.0581 | 118.1476 | 118.1789 | A(C8-C38-C39) | 117.0307 | 117.0783 | 117.1565 | 117.1537 |
| A(C37-C38-C39) | 123.0209 | 123.0551 | 122.8952 | 123.009 | A(C37-C38-C39) | 124.4932 | 124.6061 | 124.4561 | 124.638 |
| A(C38-C39-C40) | 121.7481 | 121.6075 | 121.5521 | 121.5399 | A(C38-C39-C40) | 122.8172 | 122.7778 | 122.6957 | 122.6909 |
| A(C23-C40-C39) | 121.8079 | 121.7067 | 121.6238 | 121.632 | A(C23-C40-C39) | 120.8509 | 120.8199 | 120.7479 | 120.8016 |
| A(C16-C15-F45) | 119.3737 | 118.6828 | 118.9979 | 118.52 | A(C16-C15-H46) | 120.1747 | 119.9073 | 120.0015 | 119.804 |
| A(C17-C15-F45) | 118.2477 | 118.5446 | 118.479 | 118.6369 | A(C17-C15-H46) | 119.5516 | 119.5254 | 119.6502 | 119.5638 |
| A(C17-C18-F46) | 118.2477 | 118.5446 | 118.4789 | 118.6369 | A(C20-C22-H50) | 119.1179 | 118.8476 | 119.054 | 118.7981 |
| A(C19-C18-F46) | 119.3738 | 118.6828 | 118.9979 | 118.5201 | A(C21-C22-H50) | 120.028 | 120.3185 | 120.1985 | 120.404 |
| A(C19-C20-F47) | 119.273 | 120.2509 | 120.0317 | 120.6627 | A(C23-C24-H54) | 119.3069 | 118.9038 | 119.0511 | 118.8759 |
| A(C22-C20-F47) | 118.9788 | 118.1415 | 118.4162 | 117.7975 | A(C25-C24-H54) | 119.7262 | 119.9644 | 119.9401 | 119.988 |
| A(C20-C22-F48) | 119.3625 | 118.52 | 118.8589 | 118.1932 | A(C21-C25-H47) | 119.3067 | 118.9088 | 119.0518 | 118.8757 |
| A(C21-C22-F48) | 118.8295 | 119.7734 | 119.5174 | 120.1747 | A(C24-C25-H47) | 119.7253 | 119.9518 | 119.9414 | 119.9926 |
| A(C23-C24-F50) | 121.4789 | 121.0927 | 121.2967 | 121.1801 | A(C26-C27-H45) | 119.692 | 119.4598 | 119.6535 | 119.4267 |
| A(C25-C24-F50) | 117.077 | 117.3829 | 117.3242 | 117.3167 | A(C28-C27-H45) | 120.0952 | 120.3517 | 120.2374 | 120.4193 |
| A(C21-C25-F49) | 121.4794 | 121.0923 | 121.2958 | 121.18 | A(C28-C29-H53) | 119.3245 | 118.9258 | 119.0688 | 118.9063 |
| A(C24-C25-F49) | 117.0764 | 117.3835 | 117.3252 | 117.3164 | A(C30-C29-H53) | 119.5738 | 119.7955 | 119.7714 | 119.7912 |
| A(C16-C26-F44) | 119.273 | 120.251 | 120.0317 | 120.6627 | A(C29-C30-H44) | 119.5747 | 119.784 | 119.7739 | 119.7975 |
| A(C27-C26-F44) | 118.9788 | 118.1415 | 118.4162 | 117.7975 | A(C31-C30-H44) | 119.325 | 118.931 | 119.0686 | 118.9058 |
| A(C26-C27-F43) | 119.3625 | 118.52 | 118.8589 | 118.1932 | A(C31-C32-H49) | 120.0968 | 120.3614 | 120.2378 | 120.4213 |
| A(C28-C27-F43) | 118.8296 | 119.7734 | 119.5174 | 120.1747 | A(C33-C32-H49) | 119.6935 | 119.4405 | 119.6561 | 119.4299 |
| A(C28-C29-F42) | 121.4793 | 121.0923 | 121.2958 | 121.18 | A(C34-C35-H55) | 120.1717 | 119.8546 | 119.997 | 119.8038 |
| A(C30-C29-F42) | 117.0765 | 117.3835 | 117.3252 | 117.3164 | A(C36-C35-H55) | 119.5479 | 119.5724 | 119.6506 | 119.5625 |
| A(C29-C30-F41) | 117.0771 | 117.383 | 117.3242 | 117.3167 | A(C23-C40-H48) | 120.0299 | 120.3117 | 120.1985 | 120.4005 |
| A(C31-C30-F41) | 121.4788 | 121.0927 | 121.2966 | 121.18 | A(C39-C40-H48) | 119.1192 | 118.8684 | 119.0536 | 118.7979 |
| A(C31-C32-F56) | 118.8292 | 119.7738 | 119.5182 | 120.1741 | A(C17-C18-F42) | 116.3312 | 116.6161 | 116.5508 | 116.728 |
| A(C33-C32-F56) | 119.3629 | 118.5195 | 118.8581 | 118.194 | A(C19-C18-F42) | 120.6983 | 120.0613 | 120.3876 | 119.9187 |
| A(C32-C33-F55) | 118.9787 | 118.1414 | 118.4158 | 117.798 | A(C19-C20-F51) | 117.596 | 118.2897 | 118.0268 | 118.6019 |
| A(C34-C33-F55) | 119.2732 | 120.2511 | 120.032 | 120.6622 | A(C22-C20-F51) | 119.5932 | 118.9509 | 119.282 | 118.7045 |
| A(C34-C35-F54) | 119.3744 | 118.6828 | 118.9981 | 118.52 | A(C16-C26-F41) | 116.1045 | 116.7374 | 116.4551 | 117.049 |
| A(C36-C35-F54) | 118.2473 | 118.5446 | 118.4787 | 118.6367 | A(C27-C26-F41) | 120.5136 | 119.8995 | 120.2685 | 119.6735 |
| A(C36-C37-F53) | 118.2472 | 118.5446 | 118.4787 | 118.6367 | A(C32-C33-F52) | 120.5075 | 119.9107 | 120.2595 | 119.6674 |
| A(C38-C37-F53) | 119.3744 | 118.6828 | 118.9981 | 118.52 | A(C34-C33-F52) | 116.1032 | 116.7304 | 116.4605 | 117.0527 |
| A(C38-C39-F52) | 119.2732 | 120.2511 | 120.032 | 120.6622 | A(C36-C37-F56) | 116.3296 | 116.6217 | 116.5235 | 116.716 |
| A(C40-C39-F52) | 118.9787 | 118.1414 | 118.4158 | 117.798 | A(C38-C37-F56) | 120.692 | 120.0631 | 120.4061 | 119.9295 |
| A(C23-C40-F51) | 118.8291 | 119.7738 | 119.5182 | 120.1741 | A(C38-C39-F43) | 117.5935 | 118.2745 | 118.0325 | 118.5988 |
| A(C39-C40-F51) | 119.3629 | 118.5195 | 118.8581 | 118.194 | A(C40-C39-F43) | 119.5893 | 118.9477 | 119.2719 | 118.7104 |

Table S4: The observed FT-IR, FT-Raman for some aromatic compounds and calculated wave numbers (cm^-1^) using B3LYP method with cc-pVDZ basis set and probable assignments with potential energy distribution (PED, %) for C_40_H_16_ compound.

| Circumanthracene C_40_H_16_ | | | | | | | | |  |  |
| --- | --- | --- | --- | --- | --- | --- | --- | --- | --- | --- |
| Theoretical Wavenumbers (Cm^-1^) | | | | | | | | Vibrational Assignments | Experimental | |
| Mode | RHF/ccPVDZ | | B3LYP/ccPVDZ | | WB97XD/ccPVDZ | | BPBE | with potential Energy | FT-IR | FT-Raman |
| N° | Unscaled | Scaled | Unscaled | Scaled | Unscaled | Scaled | Unscaled | distribution (PED%) B3LYP |  |  |
| 162 | 3362 | 3058 | 3189 | 3080 | 3225 | 3073 | 3125 | νCH (100) | 3158 | 3098 |
| 161 | 3361 | 3058 | 3188 | 3079 | 3224 | 3073 | 3124 | νCH (100) | 3090 | 3034 |
| 160 | 3360 | 3057 | 3187 | 3078 | 3224 | 3073 | 3124 | νCH (88) | 2959 |  |
| 159 | 3360 | 3057 | 3187 | 3078 | 3224 | 3073 | 3124 | νCH (86) |  | 3016 |
| 158 | 3356 | 3053 | 3184 | 3076 | 3222 | 3070 | 3122 | νCH (79) | 2946 |  |
| 157 | 3356 | 3053 | 3183 | 3075 | 3221 | 3070 | 3122 | νCH (82) |  |  |
| 156 | 3346 | 3044 | 3171 | 3063 | 3205 | 3054 | 3108 | νCH (73) | 2939 | 2942 |
| 155 | 3346 | 3044 | 3171 | 3063 | 3205 | 3055 | 3107 | νCH (76) |  | 3006 |
| 154 | 3341 | 3039 | 3169 | 3061 | 3204 | 3053 | 3106 | $\nu_{asym}$CH (74) | 2948 | 2763 |
| 153 | 3341 | 3039 | 3169 | 3061 | 3204 | 3053 | 3106 | $\nu_{asym}$CH (69) |  |  |
| 152 | 3339 | 3037 | 3168 | 3060 | 3204 | 3054 | 3105 | $\nu_{asym}$CH (70) | 2839 | 2841 |
| 151 | 3338 | 3037 | 3167 | 3059 | 3204 | 3054 | 3105 | $\nu_{asym}$CH (71) | 3158 | 3048 |
| 150 | 3337 | 3035 | 3167 | 3059 | 3204 | 3054 | 3105 | $\nu_{asym}$CH (69) |  |  |
| 149 | 3336 | 3035 | 3165 | 3057 | 3203 | 3052 | 3104 | $\nu_{asym}$CH (75) |  |  |
| 148 | 3335 | 3034 | 3165 | 3057 | 3203 | 3052 | 3104 | $\nu_{asym}$CH (83) |  |  |
| 147 | 3335 | 3034 | 3166 | 3058 | 3203 | 3053 | 3104 | $\nu_{asy}$CH (85) |  |  |
| 146 | 1845 | 1657 | 1677 | 1665 | 1727 | 1646 | 1629 | $\nu_{asy}$CC (14) +$\nu_{asy}$CC(12) + $\nu_{sy}$CC (13) |  |  |
| 145 | 1836 | 1649 | 1667 | 1654 | 1719 | 1638 | 1618 | $\nu_{asym}$CC (32) |  |  |
| 144 | 1821 | 1635 | 1660 | 1648 | 1704 | 1624 | 1613 | νCC (42) |  |  |
| 143 | 1814 | 1629 | 1655 | 1643 | 1703 | 1623 | 1608 | $\nu_{asym}$CC (22) |  |  |
| 142 | 1806 | 1622 | 1651 | 1639 | 1694 | 1615 | 1605 | $\nu_{asy}$CC (15) |  |  |
| 141 | 1802 | 1618 | 1647 | 1635 | 1688 | 1609 | 1600 | $\nu_{asy}$CC (20) +$\nu_{asy}$CC (26) |  |  |
| 140 | 1799 | 1616 | 1638 | 1626 | 1686 | 1607 | 1592 | $\nu_{s}$CC (19) |  |  |
| 139 | 1776 | 1595 | 1624 | 1612 | 1663 | 1585 | 1578 | $\nu_{s}$CC (26) |  |  |
| 138 | 1756 | 1577 | 1603 | 1592 | 1643 | 1566 | 1557 | νCC (56) + βHCC(11) |  |  |
| 137 | 1754 | 1575 | 1599 | 1587 | 1642 | 1565 | 1553 | νCC (12) +   νCC (20) + $\gamma$ CCC(14) |  |  |
| 136 | 1721 | 1545 | 1572 | 1561 | 1613 | 1537 | 1528 | $\nu_{asy}$CC (13) + νCC (10) + βHCC(17) |  |  |
| 135 | 1701 | 1528 | 1563 | 1552 | 1594 | 1519 | 1523 | βHCC(11) |  |  |
| 134 | 1697 | 1524 | 1550 | 1538 | 1590 | 1516 | 1504 | νCC (13) +$\nu_{asy}$CC (14) + ϒHCC(13) |  |  |
| 133 | 1684 | 1513 | 1537 | 1526 | 1588 | 1514 | 1503 | νCC (10) + βHCC(15) |  |  |
| 132 | 1672 | 1502 | 1530 | 1519 | 1574 | 1500 | 1496 | νCC (17) + βHCC(26) |  |  |
| 131 | 1646 | 1478 | 1527 | 1516 | 1555 | 1482 | 1488 | βHCC(47) |  |  |
| 130 | 1630 | 1464 | 1504 | 1493 | 1541 | 1469 | 1470 | $\nu_{asy}$CC (16)  + βHCC(13) |  |  |
| 129 | 1617 | 1452 | 1493 | 1482 | 1525 | 1454 | 1460 | $\nu_{asy}$CC (19) +$\nu_{asy}$CC (13) + νCC (10) |  |  |

Table S4: Continued

| Circumanthracene C40H16 | | | | | | | | |  |  |
| --- | --- | --- | --- | --- | --- | --- | --- | --- | --- | --- |
| Theoretical Wavenumbers (Cm^-1^) | | | | | | | | Vibrational Assignments | Experimental | |
| Mode | RHF/ccPVDZ | | B3LYP/ccPVDZ | | WB97XD/ccPVDZ | | BPBE | with potential Energy |  |  |
| N° | Unscaled | Scaled | Unscaled | Scaled | Unscaled | Scaled | Unscaled | distribution (PED%) B3LYP | FT-IR | FT-Raman |
| 128 | 1597 | 1434 | 1488 | 1477 | 1511 | 1440 | 1455 | βHCC(12) |  |  |
| 127 | 1597 | 1434 | 1483 | 1473 | 1507 | 1437 | 1447 | νCC (19) + βCCC(30) + βCCC(14) |  |  |
| 126 | 1590 | 1428 | 1466 | 1455 | 1499 | 1428 | 1435 | βHCC(22) |  |  |
| 125 | 1571 | 1411 | 1465 | 1454 | 1483 | 1414 | 1428 | $\nu_{asy}$CC (15) + βHCC(13) |  |  |
| 124 | 1557 | 1398 | 1452 | 1442 | 1475 | 1405 | 1424 | $\nu_{asy}$CC (12) + βHCC(28) |  |  |
| 123 | 1554 | 1396 | 1445 | 1435 | 1468 | 1399 | 1421 | βHCC(13) |  |  |
| 122 | 1545 | 1387 | 1442 | 1432 | 1464 | 1395 | 1414 | βCCC(36) |  |  |
| 121 | 1541 | 1384 | 1434 | 1423 | 1463 | 1394 | 1412 | $\nu_{asy}$CC (22) |  |  |
| 120 | 1533 | 1377 | 1434 | 1423 | 1454 | 1385 | 1409 | $\nu_{asy}$CC (11) |  |  |
| 119 | 1517 | 1362 | 1428 | 1417 | 1452 | 1383 | 1406 | βHCC(42) + βHCC(18) + $\gamma$HCC(11) |  |  |
| 118 | 1516 | 1361 | 1427 | 1416 | 1440 | 1373 | 1392 | $\nu_{asy}$CC (11) +νCC (10) |  |  |
| 117 | 1508 | 1354 | 1426 | 1415 | 1436 | 1368 | 1390 | βHCC(16) |  |  |
| 116 | 1472 | 1322 | 1416 | 1406 | 1432 | 1364 | 1386 | $\nu_{asy}$CC (10) + βCCC(11) |  |  |
| 115 | 1471 | 1321 | 1398 | 1388 | 1413 | 1347 | 1385 | νCC (14) |  |  |
| 114 | 1449 | 1301 | 1393 | 1382 | 1405 | 1339 | 1364 | βHCC(31) |  |  |
| 113 | 1446 | 1298 | 1370 | 1360 | 1392 | 1327 | 1351 | νCC (17) |  |  |
| 112 | 1433 | 1287 | 1363 | 1353 | 1370 | 1305 | 1351 | νCC (18) + βHCC(13) |  |  |
| 111 | 1429 | 1283 | 1353 | 1344 | 1366 | 1302 | 1319 | βCCC(16) |  |  |
| 110 | 1404 | 1261 | 1343 | 1333 | 1359 | 1295 | 1318 | $\nu_{asy}$CC (21) +νCC (20) |  |  |
| 109 | 1399 | 1256 | 1333 | 1323 | 1326 | 1264 | 1308 | νCC (17) + βHCC(21) |  |  |
| 108 | 1383 | 1242 | 1312 | 1303 | 1316 | 1254 | 1276 | $\nu_{asy}$CC (10) + βCCC(21) |  |  |
| 107 | 1377 | 1237 | 1297 | 1288 | 1313 | 1252 | 1266 | $\nu_{asy}$CC (10) + βHCC(13) |  |  |
| 106 | 1363 | 1224 | 1294 | 1285 | 1306 | 1245 | 1260 | βHCC(31) + νCC (18) |  |  |
| 105 | 1342 | 105 | 1277 | 1267 | 1291 | 1230 | 1246 | $\beta$HCC (26) + νCC (13) |  |  |
| 104 | 1337 | 1201 | 1245 | 1236 | 1256 | 1197 | 1213 | $\beta$HCC (34) + νCC (11) |  |  |
| 103 | 1329 | 1193 | 1239 | 1230 | 1249 | 1190 | 1209 | $\beta$HCC (12) + νCC (34) |  |  |
| 102 | 1328 | 1193 | 1235 | 1226 | 1241 | 1183 | 1208 | βHCC(39) + νCC (21) |  |  |
| 101 | 1310 | 1176 | 1232 | 1223 | 1241 | 1183 | 1206 | νCC (28) + βHCC(17) |  |  |
| 100 | 1299 | 1166 | 1231 | 1222 | 1234 | 1176 | 1201 | $\nu_{asy}$CC (14) + $\beta$HCC (31) |  |  |
| 99 | 1289 | 1158 | 1221 | 1212 | 1227 | 1170 | 1191 | νCC (15) + βHCC(29) |  |  |
| 98 | 1268 | 1139 | 1190 | 1182 | 1197 | 1141 | 1161 | $\gamma$HCC (53) + νCC (19) |  |  |
| 97 | 1261 | 1132 | 1187 | 1178 | 1189 | 1133 | 1157 | $\gamma$HCC (58) + $\beta$HCC (44) |  |  |
| 96 | 1259 | 1131 | 1166 | 1157 | 1179 | 1124 | 1138 | βHCC(61) |  |  |
| 95 | 1244 | 1117 | 1163 | 1154 | 1175 | 1120 | 1133 | βHCC(34) |  |  |
| 94 | 1242 | 1115 | 1163 | 1155 | 1174 | 1119 | 1132 | $\beta$HCC (50) | 1156 | 1150 |
| 93 | 1233 | 1108 | 1156 | 1148 | 1169 | 1114 | 1125 | νCC (10) + $\beta$HCC (48) |  |  |
| 92 | 1196 | 1074 | 1151 | 1142 | 1137 | 1084 | 1124 | $\upsilon_{s}$CC (10) + $\gamma$HCC (46) | 1136 | 1136 |
| 91 | 1196 | 1074 | 1127 | 1119 | 1136 | 1082 | 1099 | $\nu_{asy}$CC (10) + βCCC(11) |  |  |
| 90 | 1195 | 1074 | 1124 | 1116 | 1135 | 1082 | 1096 | $\nu_{asy}$CC (14) + $\beta$HCC(29) | 1095 |  |

Table S4: Continued

| Circumanthracene C40H16 | | | | | | | | |  |  |
| --- | --- | --- | --- | --- | --- | --- | --- | --- | --- | --- |
| Theoretical Wavenumbers (Cm) | | | | | | | | Vibrational Assignments | Experimental | |
| Mode | RHF/ccPVDZ | | B3LYP/ccPVDZ | | WB97XD/ccPVDZ | | BPBE | with potential Energy |  |  |
| N° | Unscaled | Scaled | Unscaled | Scaled | Unscaled | Scaled | Unscaled | distribution (PED%) B3LYP | FT-IR | FT-Raman |
| 89 | 1143 | 1027 | 1119 | 1111 | 1128 | 1075 | 1087 | βHCC(67) |  |  |
| 88 | 1136 | 1020 | 1077 | 1069 | 1089 | 1038 | 1051 | $\nu_{asy}$CC (11) + $\gamma$CCC(10) | 1065 |  |
| 87 | 1099 | 987 | 1047 | 1039 | 1052 | 1003 | 1024 | $\beta$CCC (16) |  |  |
| 86 | 1099 | 987 | 1002 | 994 | 1011 | 963 | 990 | $\tau$HCCC (21) | 1025 | 1020 |
| 85 | 1097 | 985 | 999 | 992 | 1010 | 963 | 975 | νCC (11) + γHCC(24) |  |  |
| 84 | 1097 | 985 | 996 | 989 | 1008 | 961 | 958 | $\tau$HCCC (47) + Out CCCC (10) |  |  |
| 83 | 1068 | 959 | 992 | 984 | 1005 | 958 | 956 | $\tau$HCCC (63) | 1010 |  |
| 82 | 1068 | 959 | 993 | 986 | 1005 | 958 | 954 | $\tau$HCCC (63) + $\tau$CCCC (15) | 985 |  |
| 81 | 1061 | 953 | 984 | 977 | 996 | 949 | 954 | $\tau$HCCC (45) |  |  |
| 80 | 1039 | 933 | 975 | 967 | 987 | 941 | 949 | $\gamma$CCC (18) |  |  |
| 79 | 1037 | 932 | 971 | 964 | 987 | 941 | 936 | $\tau$HCCC (48) |  |  |
| 78 | 1014 | 910 | 971 | 964 | 985 | 939 | 936 | $\tau$HCCC (45$\tau$HCCC (64) |  | 964 |
| 77 | 1013 | 910 | 961 | 954 | 973 | 927 | 936 | $\beta$CCC (12) | 949 | 859 |
| 76 | 1013 | 909 | 947 | 940 | 949 | 905 | 930 | $r$CH (54) + $\tau$CCCC (10) | 937 |  |
| 75 | 993 | 892 | 931 | 924 | 939 | 895 | 900 | $\tau$HCCC (39) +$\tau$CCCC (15)+$\tau$CCCC (16) | 925 |  |
| 74 | 976 | 876 | 926 | 920 | 932 | 888 | 897 | $\gamma$CCC (31) |  |  |
| 73 | 975 | 875 | 925 | 919 | 931 | 888 | 894 | $\tau$HCCC (54) + $r$CH (38) |  |  |
| 72 | 973 | 874 | 908 | 901 | 914 | 871 | 880 | $\beta$CCC (37) + $r$CH (40) |  |  |
| 71 | 950 | 853 | 896 | 889 | 900 | 858 | 868 | $\tau$HCCC (17)+ Out CCCC (34) |  |  |
| 70 | 945 | 849 | 891 | 884 | 895 | 853 | 864 | $\tau$HCCC (66) |  |  |
| 69 | 943 | 846 | 890 | 884 | 894 | 852 | 859 | $\tau$CCCC (10)+ Out CCCC (14) | 850 | 876 |
| 68 | 937 | 842 | 878 | 872 | 884 | 842 | 852 | $\tau$HCCC (26)+ Out CCCC (13) |  |  |
| 67 | 914 | 821 | 870 | 863 | 877 | 835 | 840 | $\tau$HCCC (31) |  |  |
| 66 | 897 | 805 | 864 | 858 | 869 | 828 | 838 | $\tau$HCCC (29) +$\tau$HCCC (12) +$\tau$CCCC (11) |  |  |
| 65 | 894 | 803 | 835 | 829 | 836 | 796 | 808 | $\tau$HCCC (50) |  |  |
| 64 | 889 | 798 | 834 | 828 | 836 | 797 | 807 | $\tau$HCCC (50) |  |  |
| 63 | 877 | 787 | 834 | 828 | 835 | 796 | 807 | $\tau$HCCC (40)+ Out CCCC (11) | 826 |  |
| 62 | 876 | 787 | 829 | 823 | 833 | 794 | 807 | $\upsilon$CC (25) + $\beta$CCC (10) |  |  |
| 61 | 875 | 786 | 821 | 815 | 829 | 790 | 799 | $\nu_{asy}$CC (10) + $\gamma$CCC(16) |  |  |
| 60 | 872 | 783 | 808 | 802 | 810 | 772 | 781 | $\tau$CCCC (46) |  |  |
| 59 | 853 | 766 | 793 | 787 | 801 | 764 | 769 | $\tau$HCCC (13)+ Out HCCC (54) |  |  |
| 58 | 844 | 758 | 791 | 785 | 794 | 757 | 768 | $\tau$HCCC (32) |  |  |
| 57 | 829 | 745 | 789 | 783 | 789 | 752 | 764 | $\nu_{asy}$CC (29) + $\gamma$CCC(32) |  |  |
| 56 | 823 | 739 | 783 | 777 | 789 | 752 | 762 | $\gamma$CCC(15) |  |  |
| 55 | 820 | 737 | 775 | 770 | 781 | 744 | 753 | $\gamma$CCC(63) + $\beta$CCC (10) |  |  |
| 54 | 814 | 731 | 773 | 768 | 777 | 740 | 748 | $\tau$HCCC (23) + $\tau$CCCC (10) |  |  |
| 53 | 813 | 730 | 760 | 755 | 765 | 729 | 736 | $\beta$CCC (59) + $r$CH (86) |  |  |
| 52 | 767 | 689 | 723 | 717 | 726 | 691 | 702 | $\nu_{asy}$CC (16) + $\beta$CCC(10) |  |  |
| 51 | 758 | 681 | 710 | 705 | 716 | 682 | 689 | $\gamma$CCC(41) + $\beta$CCC (14) |  |  |
| 50 | 733 | 658 | 694 | 688 | 697 | 664 | 676 | $\tau$HCCC (13)+ Out CCCC (22) |  |  |

Table S4: Continued

| Circumanthracene C40H16 | | | | | | | | |  |  |
| --- | --- | --- | --- | --- | --- | --- | --- | --- | --- | --- |
| Theoretical Wavenumbers (Cm) | | | | | | | | Vibrational Assignments | Experimental | |
| Mode | RHF/ccPVDZ | | B3LYP/ccPVDZ | | WB97XD/ccPVDZ | | BPBE | with potential Energy | FT-IR | FT-Raman |
| N° | Unscaled | Scaled | Unscaled | Scaled | Unscaled | Scaled | Unscaled | distribution (PED%) B3LYP |  |  |
| 49 | 732 | 657 | 694 | 689 | 693 | 660 | 672 | $\upsilon CC$(34)  $+\tau$CCCC (55) |  |  |
| 48 | 728 | 654 | 688 | 683 | 690 | 658 | 668 | $\tau HCC$ (22) + $OutCCC$ (13) |  |  |
| 47 | 718 | 645 | 677 | 672 | 678 | 646 | 657 | $\tau HCC$ (13) + $OutCCC$ (10) |  |  |
| 46 | 703 | 632 | 662 | 657 | 664 | 633 | 641 | $\beta$CCC (10) + $\gamma$HCC (15) |  |  |
| 45 | 686 | 616 | 650 | 645 | 650 | 620 | 629 | $\tau HCC$ (17) + $OutCCC$ (24) |  |  |
| 44 | 682 | 612 | 638 | 633 | 641 | 611 | 614 | $\beta$CCC (44) + $\gamma$CCC (13) |  |  |
| 43 | 673 | 604 | 630 | 625 | 636 | 606 | 610 | $\tau$CCCC (15)+ Out CCCC (10) |  |  |
| 42 | 661 | 594 | 623 | 618 | 624 | 595 | 603 | $\beta$CCC (22) + $\gamma$CCC (22) |  |  |
| 41 | 642 | 576 | 609 | 605 | 606 | 577 | 587 | $\tau$HCCC (12)+ Out CCCC (18) |  |  |
| 40 | 637 | 572 | 601 | 596 | 604 | 575 | 580 | $\tau$CCCC (15)+ Out CCCC (19) |  |  |
| 39 | 619 | 556 | 586 | 581 | 592 | 564 | 572 | $\upsilon CC$(11) + $\beta$CCC (49) |  |  |
| 38 | 617 | 554 | 577 | 573 | 583 | 555 | 558 | $\beta$CCC (14) + $\gamma$CCC (19) |  |  |
| 37 | 610 | 548 | 573 | 569 | 572 | 545 | 552 | $\tau$HCCC (20)+ Out CCCC (15) +$\tau$CCCC (11) |  |  |
| 36 | 604 | 542 | 570 | 565 | 571 | 545 | 551 | $\gamma$CCC (31) |  |  |
| 35 | 599 | 538 | 564 | 560 | 562 | 535 | 544 | Out CCCC (25) |  |  |
| 34 | 573 | 514 | 540 | 536 | 543 | 517 | 524 | $\beta$CCC (38) |  |  |
| 33 | 572 | 514 | 531 | 527 | 536 | 511 | 511 | $\tau$CCCC (12) |  |  |
| 32 | 543 | 488 | 510 | 506 | 513 | 489 | 493 | $\beta$CCC (36) + $\gamma$CCC (11) |  |  |
| 31 | 536 | 481 | 503 | 500 | 506 | 483 | 488 | $\beta$CCC (29) |  |  |
| 30 | 534 | 479 | 499 | 495 | 502 | 479 | 482 | $\tau$CCCC (39) |  |  |
| 29 | 531 | 477 | 496 | 492 | 499 | 475 | 478 | $\tau$CCCC (15) + $\tau$HCCC (43) |  |  |
| 28 | 523 | 470 | 494 | 490 | 496 | 473 | 478 | $\beta$CCC (19) |  |  |
| 27 | 465 | 417 | 435 | 432 | 439 | 418 | 424 | $\upsilon$ CC (19) + $\beta$CCC (10) |  |  |
| 26 | 462 | 414 | 435 | 432 | 439 | 419 | 422 | $\beta$CCC (12) + $\gamma$CCC (10) |  |  |
| 25 | 459 | 412 | 428 | 425 | 428 | 408 | 412 | $\tau$CCCC (19)+ Out CCCC (22) |  |  |
| 24 | 443 | 398 | 417 | 414 | 419 | 400 | 404 | $\upsilon$ CC (39) + $\beta$CCC (40) |  |  |
| 23 | 431 | 387 | 407 | 404 | 409 | 390 | 396 | $\beta$CCC (36) + $\gamma$CCC (12) |  |  |
| 22 | 420 | 377 | 398 | 395 | 401 | 382 | 388 | $\upsilon$ CC (14) + $\beta$CCC (16) |  |  |
| 21 | 417 | 375 | 394 | 392 | 397 | 378 | 383 | $\beta$CCC (16) + $\gamma$CCC (11) |  |  |
| 20 | 414 | 371 | 389 | 386 | 391 | 372 | 376 | $\tau$CCCC (17)+ Out CCCC (25) |  |  |
| 19 | 408 | 366 | 384 | 382 | 383 | 365 | 371 | $Out$CCCC (23) +  $\tau$CCCC (12) |  |  |
| 18 | 405 | 364 | 379 | 376 | 378 | 360 | 364 | $\tau$CCCC (11) +  $\tau$CCCC (22) |  |  |
| 17 | 370 | 332 | 350 | 348 | 347 | 331 | 338 | $\tau$CCCC (13)+ Out CCCC (22) |  |  |
| 16 | 325 | 292 | 305 | 303 | 303 | 289 | 293 | Out CCCC (24) |  |  |
| 15 | 315 | 283 | 296 | 294 | 293 | 279 | 285 | $\tau$CCCC (22) |  |  |
| 14 | 300 | 270 | 282 | 280 | 284 | 271 | 274 | $\beta$CCC (14) + $\gamma$CCC (12) |  |  |
| 13 | 294 | 264 | 276 | 274 | 279 | 265 | 269 | $\beta$CCC (13) |  |  |

Table S4: Continued

| Circumanthracene C40H16 | | | | | | | | |  |  |
| --- | --- | --- | --- | --- | --- | --- | --- | --- | --- | --- |
| Theoretical Wavenumbers (Cm) | | | | | | | | Vibrational Assignments | Experimental | |
| Mode | RHF/ccPVDZ | | B3LYP/ccPVDZ | | WB97XD/ccPVDZ | | BPBE | with potential Energy |  |  |
| N° | Unscaled | Scaled | Unscaled | Scaled | Unscaled | Scaled | Unscaled | distribution (PED%) B3LYP | FT-IR | FT-Raman |
| 12 | 288 | 258 | 270 | 268 | 267 | 255 | 259 | Out CCCC (43) + $\tau$CCCC (13) |  |  |
| 11 | 273 | 245 | 258 | 256 | 256 | 244 | 249 | Out CCCC (32) |  |  |
| 10 | 271 | 244 | 253 | 251 | 252 | 240 | 242 | $\tau$CCCC (34) |  |  |
| 9 | 224 | 201 | 211 | 210 | 212 | 202 | 205 | $\beta$CCC (20) + $\gamma$CCCC (24) |  |  |
| 8 | 219 | 197 | 204 | 203 | 203 | 194 | 196 | $\tau$CCCC (26) |  |  |
| 7 | 175 | 157 | 167 | 165 | 164 | 156 | 160 | Out CCCC (11) + $\tau$CCCC (10) |  |  |
| 6 | 167 | 150 | 155 | 154 | 153 | 146 | 149 | Out CCCC (41) |  |  |
| 5 | 119 | 106 | 112 | 111 | 110 | 104 | 106 | $\tau$CCCC (12) |  |  |
| 4 | 112 | 101 | 105 | 104 | 101 | 97 | 101 | Out CCCC (13) + $\tau$CCCC (55) |  |  |
| 3 | 108 | 97 | 102 | 101 | 100 | 95 | 97 | $\tau$CCCC (18) |  |  |
| 2 | 49 | 44 | 47 | 46 | 44 | 42 | 44 | Out CCCC (47) + $\tau$CCCC (46) |  |  |
| 1 | 46 | 41 | 45 | 44 | 43 | 41 | 43 | Out CCCC (10) + $\tau$CCCC (46) |  |  |

ν: stretching; β: in-plane-bending; γ: out-of-plane bending; τ /Out: torsion; s: symmetric; asy: asymmetric;

PED: potential energy distribution.

Table S5: Second order perturbation theory analysis of Fock matrix in NBO basis of C_40_H_16_ using B3LYP/cc-pVDZ

| Donor NBO (i) | Types | Acceptor NBO (j) | Types | E(2) | E(j)-E(i) | F (i, j) |
| --- | --- | --- | --- | --- | --- | --- |
| Circumanthracene C_40_H_16_ | |  |  | kcal/mol | a.u. | a.u. |
| σ | C 1 - C 2 | σ* | C 1 - C 6 | 4.04 | 1.26 | 0.064 |
| σ | C 1 - C 2 | σ* | C 1 - C 17 | 3.64 | 1.23 | 0.060 |
| σ | C 1 - C 2 | σ* | C 2 - C 3 | 3.72 | 1.24 | 0.061 |
| σ | C 1 - C 2 | σ* | C 2 - C 11 | 4.19 | 1.27 | 0.065 |
| σ | C 1 - C 2 | σ* | C 3 - C 14 | 2.93 | 1.27 | 0.055 |
| σ | C 1 - C 2 | σ* | C 6 - C 7 | 3.14 | 1.27 | 0.056 |
| σ | C 1 - C 2 | σ* | C 11 - C 12 | 3.17 | 1.26 | 0.056 |
| σ | C 1 - C 2 | σ* | C 17 - C 18 | 2.64 | 1.24 | 0.051 |
| σ | C 1 - C 6 | σ* | C 1 - C 2 | 4.04 | 1.26 | 0.064 |
| σ | C 1 - C 6 | σ* | C 1 - C 17 | 3.64 | 1.23 | 0.060 |
| σ | C 1 - C 6 | σ* | C 2 - C 11 | 3.14 | 1.27 | 0.056 |
| σ | C 1 - C 6 | σ* | C 5 - C 6 | 3.72 | 1.24 | 0.061 |
| σ | C 1 - C 6 | σ* | C 5 - C 8 | 2.93 | 1.27 | 0.055 |
| σ | C 1 - C 6 | σ* | C 6 - C 7 | 4.19 | 1.27 | 0.065 |
| σ | C 1 - C 6 | σ* | C 7 - C 10 | 3.17 | 1.26 | 0.056 |
| σ | C 1 - C 6 | σ* | C 15 - C 17 | 2.64 | 1.24 | 0.051 |
| π | C 1 - C 6 | π* | C 2 - C 11 | 21.97 | 0.28 | 0.071 |
| π | C 1 - C 6 | π* | C 4 - C 5 | 18.25 | 0.28 | 0.064 |
| π | C 1 - C 6 | π* | C 7 - C 10 | 22.39 | 0.28 | 0.071 |
| π | C 1 - C 6 | π* | C 17 - C 18 | 18.84 | 0.27 | 0.065 |
| σ | C 1 - C 17 | σ* | C 1 - C 2 | 3.81 | 1.25 | 0.062 |
| σ | C 1 - C 17 | σ* | C 1 - C 6 | 3.81 | 1.25 | 0.062 |
| σ | C 1 - C 17 | σ* | C 2 - C 3 | 3.32 | 1.23 | 0.057 |
| σ | C 1 - C 17 | σ* | C 5 - C 6 | 3.32 | 1.23 | 0.057 |
| σ | C 1 - C 17 | σ* | C 15 - C 17 | 3.17 | 1.23 | 0.056 |
| σ | C 1 - C 17 | σ* | C 15 - H 41 | 2.47 | 1.10 | 0.047 |
| σ | C 1 - C 17 | σ* | C 17 - C 18 | 3.17 | 1.23 | 0.056 |
| σ | C 1 - C 17 | σ* | C 18 - H 42 | 2.47 | 1.10 | 0.047 |
| σ | C 2 - C 3 | σ* | C 1 - C 2 | 3.76 | 1.25 | 0.061 |
| σ | C 2 - C 3 | σ* | C 1 - C 17 | 3.20 | 1.22 | 0.056 |
| σ | C 2 - C 3 | σ* | C 2 - C 11 | 3.86 | 1.26 | 0.062 |
| σ | C 2 - C 3 | σ* | C 3 - C 4 | 3.76 | 1.25 | 0.061 |
| σ | C 2 - C 3 | σ* | C 3 - C 14 | 3.86 | 1.26 | 0.062 |
| σ | C 2 - C 3 | σ* | C 4 - C 36 | 3.20 | 1.22 | 0.056 |
| σ | C 2 - C 3 | σ* | C 11 - C 16 | 3.23 | 1.22 | 0.056 |
| σ | C 2 - C 3 | σ* | C 14 - C 34 | 3.23 | 1.22 | 0.056 |
| σ | C 2 - C 11 | σ* | C 1 - C 2 | 4.19 | 1.27 | 0.065 |
| σ | C 2 - C 11 | σ* | C 1 - C 6 | 3.11 | 1.27 | 0.056 |
| σ | C 2 - C 11 | σ* | C 2 - C 3 | 3.84 | 1.24 | 0.062 |

Table S5: Continued

| Donor NBO (i) | Types | Acceptor NBO (j) | Types | E(2) | E(j)-E(i) | F (i, j) |
| --- | --- | --- | --- | --- | --- | --- |
| Circumanthracene C40H16 | |  |  | kcal/mol | a.u. | a.u. |
| σ | C 2 - C 11 | σ* | C 3 - C 4 | 2.93 | 1.27 | 0.055 |
| σ | C 2 - C 11 | σ* | C 11 - C 12 | 4.04 | 1.26 | 0.064 |
| σ | C 2 - C 11 | σ* | C 11 - C 16 | 3.69 | 1.23 | 0.060 |
| σ | C 2 - C 11 | σ* | C 12 - C 28 | 3.07 | 1.25 | 0.055 |
| σ | C 2 - C 11 | σ* | C 16 - C 26 | 2.64 | 1.21 | 0.051 |
| π | C 2 - C 11 | π* | C 1 - C 6 | 21.73 | 0.28 | 0.070 |
| π | C 2 - C 11 | π* | C 3 - C 14 | 18.12 | 0.29 | 0.064 |
| π | C 2 - C 11 | π* | C 12 - C 13 | 19.12 | 0.28 | 0.066 |
| π | C 2 - C 11 | π* | C 15 - C 16 | 17.44 | 0.28 | 0.064 |
| σ | C 3 - C 4 | σ* | C 2 - C 3 | 3.72 | 1.24 | 0.061 |
| σ | C 3 - C 4 | σ* | C 2 - C 11 | 2.93 | 1.27 | 0.055 |
| σ | C 3 - C 4 | σ* | C 3 - C 14 | 4.19 | 1.27 | 0.065 |
| σ | C 3 - C 4 | σ* | C 4 - C 5 | 4.04 | 1.26 | 0.064 |
| σ | C 3 - C 4 | σ* | C 4 - C 36 | 3.64 | 1.23 | 0.060 |
| σ | C 3 - C 4 | σ* | C 5 - C 8 | 3.14 | 1.27 | 0.056 |
| σ | C 3 - C 4 | σ* | C 13 - C 14 | 3.17 | 1.26 | 0.056 |
| σ | C 3 - C 4 | σ* | C 36 - C 37 | 2.64 | 1.24 | 0.051 |
| σ | C 3 - C 14 | σ* | C 1 - C 2 | 2.93 | 1.27 | 0.055 |
| σ | C 3 - C 14 | σ* | C 2 - C 3 | 3.84 | 1.24 | 0.062 |
| σ | C 3 - C 14 | σ* | C 3 - C 4 | 4.19 | 1.27 | 0.065 |
| σ | C 3 - C 14 | σ* | C 4 - C 5 | 3.11 | 1.27 | 0.056 |
| σ | C 3 - C 14 | σ* | C 13 - C 14 | 4.04 | 1.26 | 0.064 |
| σ | C 3 - C 14 | σ* | C 13 - C 31 | 3.07 | 1.25 | 0.055 |
| σ | C 3 - C 14 | σ* | C 14 - C 34 | 3.69 | 1.23 | 0.060 |
| σ | C 3 - C 14 | σ* | C 33 - C 34 | 2.64 | 1.21 | 0.051 |
| π | C 3 - C 14 | π* | C 2 - C 11 | 18.12 | 0.29 | 0.064 |
| π | C 3 - C 14 | π* | C 4 - C 5 | 21.73 | 0.28 | 0.070 |
| π | C 3 - C 14 | π* | C 12 - C 13 | 19.12 | 0.28 | 0.066 |
| π | C 3 - C 14 | π* | C 34 - C 35 | 17.44 | 0.28 | 0.064 |
| σ | C 4 - C 5 | σ* | C 3 - C 4 | 4.04 | 1.26 | 0.064 |
| σ | C 4 - C 5 | σ* | C 3 - C 14 | 3.14 | 1.27 | 0.056 |
| σ | C 4 - C 5 | σ* | C 4 - C 36 | 3.64 | 1.23 | 0.060 |
| σ | C 4 - C 5 | σ* | C 5 - C 6 | 3.72 | 1.24 | 0.061 |
| σ | C 4 - C 5 | σ* | C 5 - C 8 | 4.19 | 1.27 | 0.065 |
| σ | C 4 - C 5 | σ* | C 6 - C 7 | 2.93 | 1.27 | 0.055 |
| σ | C 4 - C 5 | σ* | C 8 - C 9 | 3.17 | 1.26 | 0.056 |
| σ | C 4 - C 5 | σ* | C 35 - C 36 | 2.64 | 1.24 | 0.051 |
| π | C 4 - C 5 | π* | C 1 - C 6 | 18.25 | 0.28 | 0.064 |
| π | C 4 - C 5 | π* | C 3 - C 14 | 21.97 | 0.28 | 0.071 |
| π | C 4 - C 5 | π* | C 8 - C 9 | 22.39 | 0.28 | 0.071 |
| π | C 4 - C 5 | π* | C 36 - C 37 | 18.84 | 0.27 | 0.065 |
| σ | C 4 - C 36 | σ* | C 2 - C 3 | 3.32 | 1.23 | 0.057 |
| σ | C 4 - C 36 | σ* | C 3 - C 4 | 3.81 | 1.25 | 0.062 |

Table S5: Continued

| Donor NBO (i) | Types | Acceptor NBO (j) | Types | E(2) | E(j)-E(i) | F (i, j) |
| --- | --- | --- | --- | --- | --- | --- |
| Circumanthracene C40H16 | |  |  | kcal/mol | a.u. | a.u. |
| σ | C 4 - C 36 | σ* | C 4 - C 5 | 3.81 | 1.25 | 0.062 |
| σ | C 4 - C 36 | σ* | C 5 - C 6 | 3.32 | 1.23 | 0.057 |
| σ | C 4 - C 36 | σ* | C 35 - C 36 | 3.17 | 1.23 | 0.056 |
| σ | C 4 - C 36 | σ* | C 35 - H 53 | 2.47 | 1.10 | 0.047 |
| σ | C 4 - C 36 | σ* | C 36 - C 37 | 3.17 | 1.23 | 0.056 |
| σ | C 4 - C 36 | σ* | C 37 - H 54 | 2.47 | 1.10 | 0.047 |
| σ | C 5 - C 6 | σ* | C 1 - C 6 | 3.76 | 1.25 | 0.061 |
| σ | C 5 - C 6 | σ* | C 1 - C 17 | 3.20 | 1.22 | 0.056 |
| σ | C 5 - C 6 | σ* | C 4 - C 5 | 3.76 | 1.25 | 0.061 |
| σ | C 5 - C 6 | σ* | C 4 - C 36 | 3.20 | 1.22 | 0.056 |
| σ | C 5 - C 6 | σ* | C 5 - C 8 | 3.86 | 1.26 | 0.062 |
| σ | C 5 - C 6 | σ* | C 6 - C 7 | 3.86 | 1.26 | 0.062 |
| σ | C 5 - C 6 | σ* | C 7 - C 19 | 3.23 | 1.22 | 0.056 |
| σ | C 5 - C 6 | σ* | C 8 - C 38 | 3.23 | 1.22 | 0.056 |
| σ | C 5 - C 8 | σ* | C 1 - C 6 | 2.93 | 1.27 | 0.055 |
| σ | C 5 - C 8 | σ* | C 3 - C 4 | 3.11 | 1.27 | 0.056 |
| σ | C 5 - C 8 | σ* | C 4 - C 5 | 4.19 | 1.27 | 0.065 |
| σ | C 5 - C 8 | σ* | C 5 - C 6 | 3.84 | 1.24 | 0.062 |
| σ | C 5 - C 8 | σ* | C 8 - C 9 | 4.04 | 1.26 | 0.064 |
| σ | C 5 - C 8 | σ* | C 8 - C 38 | 3.69 | 1.23 | 0.060 |
| σ | C 5 - C 8 | σ* | C 9 - C 23 | 3.07 | 1.25 | 0.055 |
| σ | C 5 - C 8 | σ* | C 38 - C 39 | 2.64 | 1.21 | 0.051 |
| σ | C 6 - C 7 | σ* | C 1 - C 2 | 3.11 | 1.27 | 0.056 |
| σ | C 6 - C 7 | σ* | C 1 - C 6 | 4.19 | 1.27 | 0.065 |
| σ | C 6 - C 7 | σ* | C 4 - C 5 | 2.93 | 1.27 | 0.055 |
| σ | C 6 - C 7 | σ* | C 5 - C 6 | 3.84 | 1.24 | 0.062 |
| σ | C 6 - C 7 | σ* | C 7 - C 10 | 4.04 | 1.26 | 0.064 |
| σ | C 6 - C 7 | σ* | C 7 - C 19 | 3.69 | 1.23 | 0.060 |
| σ | C 6 - C 7 | σ* | C 10 - C 21 | 3.07 | 1.25 | 0.055 |
| σ | C 6 - C 7 | σ* | C 19 - C 20 | 2.64 | 1.21 | 0.051 |
| σ | C 7 - C 10 | σ* | C 1 - C 6 | 3.24 | 1.26 | 0.057 |
| σ | C 7 - C 10 | σ* | C 6 - C 7 | 4.08 | 1.27 | 0.064 |
| σ | C 7 - C 10 | σ* | C 7 - C 19 | 3.56 | 1.22 | 0.059 |
| σ | C 7 - C 10 | σ* | C 9 - C 10 | 3.82 | 1.25 | 0.062 |
| σ | C 7 - C 10 | σ* | C 9 - C 23 | 3.02 | 1.24 | 0.055 |
| σ | C 7 - C 10 | σ* | C 10 - C 21 | 3.83 | 1.24 | 0.062 |
| σ | C 7 - C 10 | σ* | C 18 - C 19 | 2.57 | 1.27 | 0.051 |
| σ | C 7 - C 10 | σ* | C 21 - C 25 | 2.72 | 1.24 | 0.052 |
| π | C 7 - C 10 | LP ( 1) | C 19 | 50.87 | 0.14 | 0.087 |
| π | C 7 - C 10 | π* | C 1 - C 6 | 22.68 | 0.28 | 0.072 |
| π | C 7 - C 10 | π* | C 8 - C 9 | 19.29 | 0.28 | 0.066 |

Table S5: Continued

| Donor NBO (i) | Types | Acceptor NBO (j) | Types | E(2) | E(j)-E(i) | F (i, j) |
| --- | --- | --- | --- | --- | --- | --- |
| Circumanthracene C40H16 | |  |  | kcal/mol | a.u. | a.u. |
| π | C 7 - C 10 | π* | C 21 - C 25 | 21.30 | 0.27 | 0.069 |
| σ | C 7 - C 19 | σ* | C 5 - C 6 | 3.41 | 1.23 | 0.058 |
| σ | C 7 - C 19 | σ* | C 6 - C 7 | 3.86 | 1.26 | 0.062 |
| σ | C 7 - C 19 | σ* | C 7 - C 10 | 3.70 | 1.25 | 0.061 |
| σ | C 7 - C 19 | σ* | C 9 - C 10 | 3.17 | 1.24 | 0.056 |
| σ | C 7 - C 19 | σ* | C 18 - C 19 | 3.58 | 1.26 | 0.060 |
| σ | C 7 - C 19 | σ* | C 18 - H 42 | 2.70 | 1.10 | 0.049 |
| σ | C 7 - C 19 | σ* | C 19 - C 20 | 2.66 | 1.19 | 0.050 |
| σ | C 7 - C 19 | σ* | C 20 - H 43 | 2.16 | 1.11 | 0.044 |
| σ | C 8 - C 9 | σ* | C 4 - C 5 | 3.24 | 1.26 | 0.057 |
| σ | C 8 - C 9 | σ* | C 5 - C 8 | 4.08 | 1.27 | 0.064 |
| σ | C 8 - C 9 | σ* | C 8 - C 38 | 3.56 | 1.22 | 0.059 |
| σ | C 8 - C 9 | σ* | C 9 - C 10 | 3.82 | 1.25 | 0.062 |
| σ | C 8 - C 9 | σ* | C 9 - C 23 | 3.83 | 1.24 | 0.062 |
| σ | C 8 - C 9 | σ* | C 10 - C 21 | 3.02 | 1.24 | 0.055 |
| σ | C 8 - C 9 | σ* | C 23 - C 24 | 2.72 | 1.24 | 0.052 |
| σ | C 8 - C 9 | σ* | C 37 - C 38 | 2.57 | 1.27 | 0.051 |
| π | C 8 - C 9 | π* | C 4 - C 5 | 22.68 | 0.28 | 0.072 |
| π | C 8 - C 9 | π* | C 7 - C 10 | 19.29 | 0.28 | 0.066 |
| π | C 8 - C 9 | π* | C 23 - C 24 | 21.30 | 0.27 | 0.069 |
| σ | C 8 - C 38 | σ* | C 5 - C 6 | 3.41 | 1.23 | 0.058 |
| σ | C 8 - C 38 | σ* | C 5 - C 8 | 3.86 | 1.26 | 0.062 |
| σ | C 8 - C 38 | σ* | C 8 - C 9 | 3.70 | 1.25 | 0.061 |
| σ | C 8 - C 38 | σ* | C 9 - C 10 | 3.17 | 1.24 | 0.056 |
| σ | C 8 - C 38 | σ* | C 37 - C 38 | 3.58 | 1.26 | 0.060 |
| σ | C 8 - C 38 | σ* | C 37 - H 54 | 2.70 | 1.10 | 0.049 |
| σ | C 8 - C 38 | σ* | C 38 - C 39 | 2.66 | 1.19 | 0.050 |
| σ | C 8 - C 38 | σ* | C 39 - H 55 | 2.16 | 1.11 | 0.044 |
| σ | C 9 - C 10 | σ* | C 7 - C 10 | 3.87 | 1.25 | 0.062 |
| σ | C 9 - C 10 | σ* | C 7 - C 19 | 3.17 | 1.22 | 0.056 |
| σ | C 9 - C 10 | σ* | C 8 - C 9 | 3.87 | 1.25 | 0.062 |
| σ | C 9 - C 10 | σ* | C 8 - C 38 | 3.17 | 1.22 | 0.056 |
| σ | C 9 - C 10 | σ* | C 9 - C 23 | 3.80 | 1.24 | 0.061 |
| σ | C 9 - C 10 | σ* | C 10 - C 21 | 3.80 | 1.24 | 0.061 |
| σ | C 9 - C 10 | σ* | C 21 - C 22 | 2.82 | 1.20 | 0.052 |
| σ | C 9 - C 10 | σ* | C 23 - C 40 | 2.82 | 1.20 | 0.052 |
| σ | C 9 - C 23 | σ* | C 5 - C 8 | 3.11 | 1.27 | 0.056 |
| σ | C 9 - C 23 | σ* | C 7 - C 10 | 3.13 | 1.25 | 0.056 |
| σ | C 9 - C 23 | σ* | C 8 - C 9 | 3.96 | 1.25 | 0.063 |
| σ | C 9 - C 23 | σ* | C 9 - C 10 | 3.91 | 1.25 | 0.062 |
| σ | C 9 - C 23 | σ* | C 23 - C 24 | 3.45 | 1.24 | 0.059 |
| σ | C 9 - C 23 | σ* | C 23 - C 40 | 2.91 | 1.20 | 0.053 |
| σ | C 9 - C 23 | σ* | C 24 - H 45 | 2.45 | 1.12 | 0.047 |
| σ | C 9 - C 23 | σ* | C 40 - H 56 | 2.14 | 1.12 | 0.044 |
| σ | C 10 - C 21 | σ* | C 6 - C 7 | 3.11 | 1.27 | 0.056 |
| σ | C 10 - C 21 | σ* | C 7 - C 10 | 3.96 | 1.25 | 0.063 |
| σ | C 10 - C 21 | σ* | C 8 - C 9 | 3.13 | 1.25 | 0.056 |
| σ | C 10 - C 21 | σ* | C 9 - C 10 | 3.91 | 1.25 | 0.062 |
| σ | C 10 - C 21 | σ* | C 21 - C 22 | 2.91 | 1.20 | 0.053 |
| σ | C 10 - C 21 | σ* | C 21 - C 25 | 3.45 | 1.24 | 0.059 |
| σ | C 10 - C 21 | σ* | C 22 - H 44 | 2.14 | 1.12 | 0.044 |
| σ | C 10 - C 21 | σ* | C 25 - H 46 | 2.45 | 1.12 | 0.047 |
| σ | C 11 - C 12 | σ* | C 1 - C 2 | 3.24 | 1.26 | 0.057 |
| σ | C 11 - C 12 | σ* | C 2 - C 11 | 4.08 | 1.27 | 0.064 |
| σ | C 11 - C 12 | σ* | C 11 - C 16 | 3.56 | 1.22 | 0.059 |
| σ | C 11 - C 12 | σ* | C 12 - C 13 | 3.82 | 1.25 | 0.062 |
| σ | C 11 - C 12 | σ* | C 12 - C 28 | 3.83 | 1.24 | 0.062 |
| σ | C 11 - C 12 | σ* | C 13 - C 31 | 3.02 | 1.24 | 0.055 |
| σ | C 11 - C 12 | σ* | C 15 - C 16 | 2.57 | 1.27 | 0.051 |
| σ | C 11 - C 12 | σ* | C 28 - C 29 | 2.72 | 1.24 | 0.052 |
| σ | C 11 - C 16 | σ* | C 2 - C 3 | 3.41 | 1.23 | 0.058 |
| σ | C 11 - C 16 | σ* | C 2 - C 11 | 3.86 | 1.26 | 0.062 |
| σ | C 11 - C 16 | σ* | C 11 - C 12 | 3.70 | 1.25 | 0.061 |
| σ | C 11 - C 16 | σ* | C 12 - C 13 | 3.17 | 1.24 | 0.056 |
| σ | C 11 - C 16 | σ* | C 15 - C 16 | 3.58 | 1.26 | 0.060 |
| σ | C 11 - C 16 | σ* | C 15 - H 41 | 2.70 | 1.10 | 0.049 |
| σ | C 11 - C 16 | σ* | C 16 - C 26 | 2.66 | 1.19 | 0.050 |
| σ | C 11 - C 16 | σ* | C 26 - H 47 | 2.16 | 1.11 | 0.044 |
| σ | C 12 - C 13 | σ* | C 11 - C 12 | 3.87 | 1.25 | 0.062 |

Table S5: Continued

| Donor NBO (i) | Types | Acceptor NBO (j) | Types | E(2) | E(j)-E(i) | F (i, j) |
| --- | --- | --- | --- | --- | --- | --- |
| Circumanthracene C40H16 | |  |  | kcal/mol | a.u. | a.u. |
| σ | C 12 - C 13 | σ* | C 11 - C 16 | 3.17 | 1.22 | 0.056 |
| σ | C 12 - C 13 | σ* | C 12 - C 28 | 3.80 | 1.24 | 0.061 |
| σ | C 12 - C 13 | σ* | C 13 - C 14 | 3.87 | 1.25 | 0.062 |
| σ | C 12 - C 13 | σ* | C 13 - C 31 | 3.80 | 1.24 | 0.061 |
| σ | C 12 - C 13 | σ* | C 14 - C 34 | 3.17 | 1.22 | 0.056 |
| σ | C 12 - C 13 | σ* | C 27 - C 28 | 2.82 | 1.20 | 0.052 |
| σ | C 12 - C 13 | σ* | C 31 - C 32 | 2.82 | 1.20 | 0.052 |
| π | C 12 - C 13 | π* | C 2 - C 11 | 18.78 | 0.28 | 0.066 |
| π | C 12 - C 13 | π* | C 3 - C 14 | 18.78 | 0.28 | 0.066 |
| π | C 12 - C 13 | π* | C 28 - C 29 | 19.47 | 0.27 | 0.066 |
| π | C 12 - C 13 | π* | C 30 - C 31 | 19.47 | 0.27 | 0.066 |
| σ | C 12 - C 28 | σ* | C 2 - C 11 | 3.11 | 1.27 | 0.056 |
| σ | C 12 - C 28 | σ* | C 11 - C 12 | 3.96 | 1.25 | 0.063 |
| σ | C 12 - C 28 | σ* | C 12 - C 13 | 3.91 | 1.25 | 0.062 |
| σ | C 12 - C 28 | σ* | C 13 - C 14 | 3.13 | 1.25 | 0.056 |
| σ | C 12 - C 28 | σ* | C 27 - C 28 | 2.91 | 1.20 | 0.053 |
| σ | C 12 - C 28 | σ* | C 27 - H 48 | 2.14 | 1.12 | 0.044 |
| σ | C 12 - C 28 | σ* | C 28 - C 29 | 3.45 | 1.24 | 0.059 |
| σ | C 12 - C 28 | σ* | C 29 - H 49 | 2.45 | 1.12 | 0.047 |
| σ | C 13 - C 14 | σ* | C 3 - C 4 | 3.24 | 1.26 | 0.057 |
| σ | C 13 - C 14 | σ* | C 3 - C 14 | 4.08 | 1.27 | 0.064 |
| σ | C 13 - C 14 | σ* | C 12 - C 13 | 3.82 | 1.25 | 0.062 |
| σ | C 13 - C 14 | σ* | C 12 - C 28 | 3.02 | 1.24 | 0.055 |
| σ | C 13 - C 14 | σ* | C 13 - C 31 | 3.83 | 1.24 | 0.062 |
| σ | C 13 - C 14 | σ* | C 14 - C 34 | 3.56 | 1.22 | 0.059 |
| σ | C 13 - C 14 | σ* | C 30 - C 31 | 2.72 | 1.24 | 0.052 |
| σ | C 13 - C 14 | σ* | C 34 - C 35 | 2.57 | 1.27 | 0.051 |
| σ | C 13 - C 31 | σ* | C 3 - C 14 | 3.11 | 1.27 | 0.056 |
| σ | C 13 - C 31 | σ* | C 11 - C 12 | 3.13 | 1.25 | 0.056 |
| σ | C 13 - C 31 | σ* | C 12 - C 13 | 3.91 | 1.25 | 0.062 |
| σ | C 13 - C 31 | σ* | C 13 - C 14 | 3.96 | 1.25 | 0.063 |
| σ | C 13 - C 31 | σ* | C 30 - C 31 | 3.45 | 1.24 | 0.059 |
| σ | C 13 - C 31 | σ* | C 30 - H 50 | 2.45 | 1.12 | 0.047 |
| σ | C 13 - C 31 | σ* | C 31 - C 32 | 2.91 | 1.20 | 0.053 |
| σ | C 13 - C 31 | σ* | C 32 - H 51 | 2.14 | 1.12 | 0.044 |
| σ | C 14 - C 34 | σ* | C 2 - C 3 | 3.41 | 1.23 | 0.058 |
| σ | C 14 - C 34 | σ* | C 3 - C 14 | 3.86 | 1.26 | 0.062 |
| σ | C 14 - C 34 | σ* | C 12 - C 13 | 3.17 | 1.24 | 0.056 |
| σ | C 14 - C 34 | σ* | C 13 - C 14 | 3.70 | 1.25 | 0.061 |
| σ | C 14 - C 34 | σ* | C 33 - C 34 | 2.66 | 1.19 | 0.050 |
| σ | C 14 - C 34 | σ* | C 33 - H 52 | 2.16 | 1.11 | 0.044 |
| σ | C 14 - C 34 | σ* | C 34 - C 35 | 3.58 | 1.26 | 0.060 |
| σ | C 14 - C 34 | σ* | C 35 - H 53 | 2.70 | 1.10 | 0.049 |
| σ | C 15 - C 16 | σ* | C 11 - C 12 | 3.08 | 1.28 | 0.056 |
| σ | C 15 - C 16 | σ* | C 11 - C 16 | 4.07 | 1.25 | 0.064 |
| σ | C 15 - C 16 | σ* | C 15 - C 17 | 3.09 | 1.27 | 0.056 |
| σ | C 15 - C 16 | σ* | C 15 - H 41 | 1.25 | 1.13 | 0.034 |
| σ | C 15 - C 16 | σ* | C 16 - C 26 | 3.48 | 1.23 | 0.058 |
| σ | C 15 - C 16 | σ* | C 17 - C 18 | 3.45 | 1.27 | 0.059 |
| σ | C 15 - C 16 | σ* | C 26 - C 27 | 1.73 | 1.33 | 0.043 |
| π | C 15 - C 16 | π* | C 2 - C 11 | 16.17 | 0.30 | 0.063 |
| π | C 15 - C 16 | π* | C 17 - C 18 | 20.48 | 0.29 | 0.070 |
| π | C 15 - C 16 | π* | C 26 - C 27 | 17.22 | 0.30 | 0.066 |
| σ | C 15 - C 17 | σ* | C 1 - C 6 | 3.17 | 1.27 | 0.057 |
| σ | C 15 - C 17 | σ* | C 1 - C 17 | 3.65 | 1.24 | 0.060 |
| σ | C 15 - C 17 | σ* | C 15 - C 16 | 3.14 | 1.28 | 0.057 |
| σ | C 15 - C 17 | σ* | C 15 - H 41 | 1.03 | 1.12 | 0.030 |
| σ | C 15 - C 17 | σ* | C 16 - C 26 | 3.83 | 1.21 | 0.061 |
| σ | C 15 - C 17 | σ* | C 17 - C 18 | 3.61 | 1.25 | 0.060 |
| σ | C 15 - C 17 | σ* | C 18 - C 19 | 2.16 | 1.28 | 0.047 |
| σ | C 15 - H 41 | σ* | C 1 - C 17 | 4.81 | 1.07 | 0.064 |
| σ | C 15 - H 41 | σ* | C 11 - C 16 | 5.23 | 1.07 | 0.067 |
| σ | C 15 - H 41 | σ* | C 15 - C 16 | 0.98 | 1.12 | 0.030 |
| σ | C 15 - H 41 | σ* | C 15 - C 17 | 0.72 | 1.09 | 0.025 |
| σ | C 16 - C 26 | σ* | C 2 - C 11 | 3.26 | 1.26 | 0.057 |
| σ | C 16 - C 26 | σ* | C 11 - C 16 | 3.07 | 1.22 | 0.055 |
| σ | C 16 - C 26 | σ* | C 15 - C 16 | 3.63 | 1.27 | 0.061 |
| σ | C 16 - C 26 | σ* | C 15 - C 17 | 2.56 | 1.24 | 0.050 |
| σ | C 16 - C 26 | σ* | C 26 - C 27 | 2.80 | 1.30 | 0.054 |
| σ | C 16 - C 26 | σ* | C 26 - H 47 | 0.75 | 1.11 | 0.026 |
| σ | C 16 - C 26 | σ* | C 27 - H 48 | 3.06 | 1.11 | 0.052 |
| σ | C 17 - C 18 | σ* | C 1 - C 2 | 3.17 | 1.27 | 0.057 |
| σ | C 17 - C 18 | σ* | C 1 - C 17 | 3.65 | 1.24 | 0.060 |
| σ | C 17 - C 18 | σ* | C 15 - C 16 | 2.16 | 1.28 | 0.047 |
| σ | C 17 - C 18 | σ* | C 15 - C 17 | 3.61 | 1.25 | 0.060 |
| σ | C 17 - C 18 | σ* | C 18 - C 19 | 3.14 | 1.28 | 0.057 |
| σ | C 17 - C 18 | σ* | C 18 - H 42 | 1.03 | 1.12 | 0.030 |
| σ | C 17 - C 18 | σ* | C 19 - C 20 | 3.83 | 1.21 | 0.061 |
| π | C 17 - C 18 | π* | C 1 - C 6 | 17.66 | 0.29 | 0.064 |
| π | C 17 - C 18 | π* | C 15 - C 16 | 20.66 | 0.28 | 0.070 |
| σ | C 18 - C 19 | σ* | C 7 - C 10 | 3.08 | 1.28 | 0.056 |
| σ | C 18 - C 19 | σ* | C 7 - C 19 | 4.07 | 1.25 | 0.064 |
| σ | C 18 - C 19 | σ* | C 15 - C 17 | 3.45 | 1.27 | 0.059 |
| σ | C 18 - C 19 | σ* | C 17 - C 18 | 3.09 | 1.27 | 0.056 |
| σ | C 18 - C 19 | σ* | C 18 - H 42 | 1.25 | 1.13 | 0.034 |
| σ | C 18 - C 19 | σ* | C 19 - C 20 | 3.48 | 1.23 | 0.058 |
| σ | C 18 - C 19 | σ* | C 20 - C 22 | 1.73 | 1.33 | 0.043 |
| σ | C 18 - H 42 | σ* | C 1 - C 17 | 4.81 | 1.07 | 0.064 |
| σ | C 18 - H 42 | σ* | C 7 - C 19 | 5.23 | 1.07 | 0.067 |
| σ | C 18 - H 42 | σ* | C 17 - C 18 | 0.72 | 1.09 | 0.025 |
| σ | C 18 - H 42 | σ* | C 18 - C 19 | 0.98 | 1.12 | 0.030 |
| σ | C 19 - C 20 | σ* | C 6 - C 7 | 3.26 | 1.26 | 0.057 |
| σ | C 19 - C 20 | σ* | C 7 - C 19 | 3.07 | 1.22 | 0.055 |
| σ | C 19 - C 20 | σ* | C 17 - C 18 | 2.56 | 1.24 | 0.050 |
| σ | C 19 - C 20 | σ* | C 18 - C 19 | 3.63 | 1.27 | 0.061 |
| σ | C 19 - C 20 | σ* | C 20 - C 22 | 2.80 | 1.30 | 0.054 |
| σ | C 19 - C 20 | σ* | C 20 - H 43 | 0.75 | 1.11 | 0.026 |
| σ | C 19 - C 20 | σ* | C 22 - H 44 | 3.06 | 1.11 | 0.052 |
| σ | C 20 - C 22 | σ* | C 18 - C 19 | 3.20 | 1.32 | 0.058 |
| σ | C 20 - C 22 | σ* | C 19 - C 20 | 2.93 | 1.25 | 0.054 |
| σ | C 20 - C 22 | σ* | C 20 - H 43 | 1.53 | 1.16 | 0.038 |
| σ | C 20 - C 22 | σ* | C 21 - C 22 | 3.00 | 1.25 | 0.055 |
| σ | C 20 - C 22 | σ* | C 21 - C 25 | 3.21 | 1.29 | 0.058 |
| σ | C 20 - C 22 | σ* | C 22 - H 44 | 1.52 | 1.16 | 0.038 |
| π | C 20 - C 22 | π* | C 21 - C 25 | 15.82 | 0.30 | 0.065 |
| σ | C 20 - H 43 | σ* | C 7 - C 19 | 4.45 | 1.07 | 0.062 |
| σ | C 20 - H 43 | σ* | C 20 - C 22 | 1.10 | 1.15 | 0.032 |
| σ | C 20 - H 43 | σ* | C 21 - C 22 | 4.92 | 1.05 | 0.064 |
| σ | C 21 - C 22 | σ* | C 9 - C 10 | 3.45 | 1.25 | 0.059 |
| σ | C 21 - C 22 | σ* | C 10 - C 21 | 3.38 | 1.24 | 0.058 |
| σ | C 21 - C 22 | σ* | C 20 - C 22 | 2.86 | 1.31 | 0.055 |
| σ | C 21 - C 22 | σ* | C 20 - H 43 | 3.04 | 1.11 | 0.052 |
| σ | C 21 - C 22 | σ* | C 21 - C 25 | 3.28 | 1.24 | 0.057 |
| σ | C 21 - C 22 | σ* | C 22 - H 44 | 0.78 | 1.11 | 0.026 |
| σ | C 21 - C 22 | σ* | C 24 - C 25 | 2.18 | 1.27 | 0.047 |
| σ | C 21 - C 25 | σ* | C 7 - C 10 | 3.38 | 1.27 | 0.058 |
| σ | C 21 - C 25 | σ* | C 10 - C 21 | 3.94 | 1.26 | 0.063 |
| σ | C 21 - C 25 | σ* | C 20 - C 22 | 1.81 | 1.32 | 0.044 |
| σ | C 21 - C 25 | σ* | C 21 - C 22 | 3.25 | 1.22 | 0.056 |
| σ | C 21 - C 25 | σ* | C 24 - C 25 | 2.85 | 1.29 | 0.054 |
| σ | C 21 - C 25 | σ* | C 24 - H 45 | 2.68 | 1.13 | 0.049 |
| σ | C 21 - C 25 | σ* | C 25 - H 46 | 1.02 | 1.13 | 0.030 |
| π | C 21 - C 25 | π* | C 7 - C 10 | 20.28 | 0.29 | 0.069 |
| π | C 21 - C 25 | π* | C 20 - C 22 | 17.24 | 0.29 | 0.067 |
| π | C 21 - C 25 | π* | C 23 - C 24 | 21.90 | 0.28 | 0.070 |
| σ | C 22 - H 44 | σ* | C 10 - C 21 | 4.46 | 1.09 | 0.062 |
| σ | C 22 - H 44 | σ* | C 19 - C 20 | 4.92 | 1.05 | 0.064 |
| σ | C 22 - H 44 | σ* | C 20 - C 22 | 1.08 | 1.15 | 0.032 |
| σ | C 23 - C 24 | σ* | C 8 - C 9 | 3.38 | 1.27 | 0.058 |
| σ | C 23 - C 24 | σ* | C 9 - C 23 | 3.94 | 1.26 | 0.063 |
| σ | C 23 - C 24 | σ* | C 23 - C 40 | 3.25 | 1.22 | 0.056 |
| σ | C 23 - C 24 | σ* | C 24 - C 25 | 2.85 | 1.29 | 0.054 |
| σ | C 23 - C 24 | σ* | C 24 - H 45 | 1.02 | 1.13 | 0.030 |
| σ | C 23 - C 24 | σ* | C 25 - H 46 | 2.68 | 1.13 | 0.049 |
| σ | C 23 - C 24 | σ* | C 39 - C 40 | 1.81 | 1.32 | 0.044 |
| π | C 23 - C 24 | π* | C 8 - C 9 | 20.28 | 0.29 | 0.069 |
| π | C 23 - C 24 | π* | C 21 - C 25 | 21.90 | 0.28 | 0.070 |
| π | C 23 - C 24 | π* | C 39 - C 40 | 17.24 | 0.29 | 0.067 |
| σ | C 23 - C 40 | σ* | C 9 - C 10 | 3.45 | 1.25 | 0.059 |
| σ | C 23 - C 40 | σ* | C 9 - C 23 | 3.38 | 1.24 | 0.058 |
| σ | C 23 - C 40 | σ* | C 23 - C 24 | 3.28 | 1.24 | 0.057 |
| σ | C 23 - C 40 | σ* | C 24 - C 25 | 2.18 | 1.27 | 0.047 |
| σ | C 23 - C 40 | σ* | C 39 - C 40 | 2.86 | 1.31 | 0.055 |
| σ | C 23 - C 40 | σ* | C 39 - H 55 | 3.04 | 1.11 | 0.052 |
| σ | C 23 - C 40 | σ* | C 40 - H 56 | 0.78 | 1.11 | 0.026 |
| σ | C 24 - C 25 | σ* | C 21 - C 22 | 3.73 | 1.24 | 0.061 |
| σ | C 24 - C 25 | σ* | C 21 - C 25 | 3.15 | 1.27 | 0.057 |
| σ | C 24 - C 25 | σ* | C 23 - C 24 | 3.15 | 1.27 | 0.057 |
| σ | C 24 - C 25 | σ* | C 23 - C 40 | 3.73 | 1.24 | 0.061 |
| σ | C 24 - C 25 | σ* | C 24 - H 45 | 1.28 | 1.15 | 0.034 |
| σ | C 24 - C 25 | σ* | C 25 - H 46 | 1.28 | 1.15 | 0.034 |
| σ | C 24 - H 45 | σ* | C 9 - C 23 | 4.90 | 1.09 | 0.065 |
| σ | C 24 - H 45 | σ* | C 21 - C 25 | 4.37 | 1.09 | 0.062 |
| σ | C 24 - H 45 | σ* | C 23 - C 24 | 0.70 | 1.09 | 0.025 |
| σ | C 24 - H 45 | σ* | C 24 - C 25 | 0.78 | 1.12 | 0.026 |
| σ | C 25 - H 46 | σ* | C 10 - C 21 | 4.90 | 1.09 | 0.065 |
| σ | C 25 - H 46 | σ* | C 21 - C 25 | 0.70 | 1.09 | 0.025 |
| σ | C 25 - H 46 | σ* | C 23 - C 24 | 4.37 | 1.09 | 0.062 |
| σ | C 25 - H 46 | σ* | C 24 - C 25 | 0.78 | 1.12 | 0.026 |
| σ | C 26 - C 27 | σ* | C 15 - C 16 | 3.20 | 1.32 | 0.058 |
| σ | C 26 - C 27 | σ* | C 16 - C 26 | 2.93 | 1.25 | 0.054 |
| σ | C 26 - C 27 | σ* | C 26 - H 47 | 1.53 | 1.16 | 0.038 |
| σ | C 26 - C 27 | σ* | C 27 - C 28 | 3.00 | 1.25 | 0.055 |
| σ | C 26 - C 27 | σ* | C 27 - H 48 | 1.52 | 1.16 | 0.038 |
| σ | C 26 - C 27 | σ* | C 28 - C 29 | 3.21 | 1.29 | 0.058 |
| π | C 26 - C 27 | π* | C 15 - C 16 | 16.24 | 0.31 | 0.065 |
| π | C 26 - C 27 | π* | C 28 - C 29 | 15.82 | 0.30 | 0.065 |
| σ | C 26 - H 47 | σ* | C 11 - C 16 | 4.45 | 1.07 | 0.062 |
| σ | C 26 - H 47 | σ* | C 26 - C 27 | 1.10 | 1.15 | 0.032 |
| σ | C 26 - H 47 | σ* | C 27 - C 28 | 4.92 | 1.05 | 0.064 |
| σ | C 27 - C 28 | σ* | C 12 - C 13 | 3.45 | 1.25 | 0.059 |
| σ | C 27 - C 28 | σ* | C 12 - C 28 | 3.38 | 1.24 | 0.058 |
| σ | C 27 - C 28 | σ* | C 26 - C 27 | 2.86 | 1.31 | 0.055 |
| σ | C 27 - C 28 | σ* | C 26 - H 47 | 3.04 | 1.11 | 0.052 |
| σ | C 27 - C 28 | σ* | C 27 - H 48 | 0.78 | 1.11 | 0.026 |
| σ | C 27 - C 28 | σ* | C 28 - C 29 | 3.28 | 1.24 | 0.057 |
| σ | C 27 - C 28 | σ* | C 29 - C 30 | 2.18 | 1.27 | 0.047 |
| σ | C 27 - H 48 | σ* | C 12 - C 28 | 4.46 | 1.09 | 0.062 |
| σ | C 27 - H 48 | σ* | C 16 - C 26 | 4.92 | 1.05 | 0.064 |
| σ | C 27 - H 48 | σ* | C 26 - C 27 | 1.08 | 1.15 | 0.032 |
| σ | C 28 - C 29 | σ* | C 11 - C 12 | 3.38 | 1.27 | 0.058 |
| σ | C 28 - C 29 | σ* | C 12 - C 28 | 3.94 | 1.26 | 0.063 |
| σ | C 28 - C 29 | σ* | C 26 - C 27 | 1.81 | 1.32 | 0.044 |
| σ | C 28 - C 29 | σ* | C 27 - C 28 | 3.25 | 1.22 | 0.056 |
| σ | C 28 - C 29 | σ* | C 29 - C 30 | 2.85 | 1.29 | 0.054 |
| σ | C 28 - C 29 | σ* | C 29 - H 49 | 1.02 | 1.13 | 0.030 |
| σ | C 28 - C 29 | σ* | C 30 - H 50 | 2.68 | 1.13 | 0.049 |
| π | C 28 - C 29 | π* | C 12 - C 13 | 18.46 | 0.29 | 0.066 |
| π | C 28 - C 29 | π* | C 26 - C 27 | 17.24 | 0.29 | 0.067 |
| π | C 28 - C 29 | π* | C 30 - C 31 | 21.90 | 0.28 | 0.070 |
| σ | C 29 - C 30 | σ* | C 27 - C 28 | 3.73 | 1.24 | 0.061 |
| σ | C 29 - C 30 | σ* | C 28 - C 29 | 3.15 | 1.27 | 0.057 |
| σ | C 29 - C 30 | σ* | C 29 - H 49 | 1.28 | 1.15 | 0.034 |
| σ | C 29 - C 30 | σ* | C 30 - C 31 | 3.15 | 1.27 | 0.057 |
| σ | C 29 - C 30 | σ* | C 30 - H 50 | 1.28 | 1.15 | 0.034 |
| σ | C 29 - C 30 | σ* | C 31 - C 32 | 3.73 | 1.24 | 0.061 |
| σ | C 29 - H 49 | σ* | C 12 - C 28 | 4.90 | 1.09 | 0.065 |
| σ | C 29 - H 49 | σ* | C 28 - C 29 | 0.70 | 1.09 | 0.025 |
| σ | C 29 - H 49 | σ* | C 29 - C 30 | 0.78 | 1.12 | 0.026 |
| σ | C 29 - H 49 | σ* | C 30 - C 31 | 4.37 | 1.09 | 0.062 |
| σ | C 30 - C 31 | σ* | C 13 - C 14 | 3.38 | 1.27 | 0.058 |
| σ | C 30 - C 31 | σ* | C 13 - C 31 | 3.94 | 1.26 | 0.063 |
| σ | C 30 - C 31 | σ* | C 29 - C 30 | 2.85 | 1.29 | 0.054 |
| σ | C 30 - C 31 | σ* | C 29 - H 49 | 2.68 | 1.13 | 0.049 |
| σ | C 30 - C 31 | σ* | C 30 - H 50 | 1.02 | 1.13 | 0.030 |
| σ | C 30 - C 31 | σ* | C 31 - C 32 | 3.25 | 1.22 | 0.056 |
| σ | C 30 - C 31 | σ* | C 32 - C 33 | 1.81 | 1.32 | 0.044 |
| π | C 30 - C 31 | π* | C 12 - C 13 | 18.46 | 0.29 | 0.066 |
| π | C 30 - C 31 | π* | C 28 - C 29 | 21.90 | 0.28 | 0.070 |
| π | C 30 - C 31 | π* | C 32 - C 33 | 17.24 | 0.29 | 0.067 |
| σ | C 30 - H 50 | σ* | C 13 - C 31 | 4.90 | 1.09 | 0.065 |
| σ | C 30 - H 50 | σ* | C 28 - C 29 | 4.37 | 1.09 | 0.062 |
| σ | C 30 - H 50 | σ* | C 29 - C 30 | 0.78 | 1.12 | 0.026 |
| σ | C 30 - H 50 | σ* | C 30 - C 31 | 0.70 | 1.09 | 0.025 |
| σ | C 31 - C 32 | σ* | C 12 - C 13 | 3.45 | 1.25 | 0.059 |
| σ | C 31 - C 32 | σ* | C 13 - C 31 | 3.38 | 1.24 | 0.058 |
| σ | C 31 - C 32 | σ* | C 29 - C 30 | 2.18 | 1.27 | 0.047 |
| σ | C 31 - C 32 | σ* | C 30 - C 31 | 3.28 | 1.24 | 0.057 |
| σ | C 31 - C 32 | σ* | C 32 - C 33 | 2.86 | 1.31 | 0.055 |
| σ | C 31 - C 32 | σ* | C 32 - H 51 | 0.78 | 1.11 | 0.026 |
| σ | C 31 - C 32 | σ* | C 33 - H 52 | 3.04 | 1.11 | 0.052 |
| σ | C 32 - C 33 | σ* | C 30 - C 31 | 3.21 | 1.29 | 0.058 |
| σ | C 32 - C 33 | σ* | C 31 - C 32 | 3.00 | 1.25 | 0.055 |
| σ | C 32 - C 33 | σ* | C 32 - H 51 | 1.52 | 1.16 | 0.038 |
| σ | C 32 - C 33 | σ* | C 33 - C 34 | 2.93 | 1.25 | 0.054 |
| σ | C 32 - C 33 | σ* | C 33 - H 52 | 1.53 | 1.16 | 0.038 |
| σ | C 32 - C 33 | σ* | C 34 - C 35 | 3.20 | 1.32 | 0.058 |
| π | C 32 - C 33 | π* | C 30 - C 31 | 15.82 | 0.30 | 0.065 |
| π | C 32 - C 33 | π* | C 34 - C 35 | 16.24 | 0.31 | 0.065 |
| σ | C 32 - H 51 | σ* | C 13 - C 31 | 4.46 | 1.09 | 0.062 |
| σ | C 32 - H 51 | σ* | C 32 - C 33 | 1.08 | 1.15 | 0.032 |
| σ | C 32 - H 51 | σ* | C 33 - C 34 | 4.92 | 1.05 | 0.064 |
| σ | C 33 - C 34 | σ* | C 3 - C 14 | 3.26 | 1.26 | 0.057 |
| σ | C 33 - C 34 | σ* | C 14 - C 34 | 3.07 | 1.22 | 0.055 |
| σ | C 33 - C 34 | σ* | C 32 - C 33 | 2.80 | 1.30 | 0.054 |
| σ | C 33 - C 34 | σ* | C 32 - H 51 | 3.06 | 1.11 | 0.052 |
| σ | C 33 - C 34 | σ* | C 33 - H 52 | 0.75 | 1.11 | 0.026 |
| σ | C 33 - C 34 | σ* | C 34 - C 35 | 3.63 | 1.27 | 0.061 |
| σ | C 33 - C 34 | σ* | C 35 - C 36 | 2.56 | 1.24 | 0.050 |
| σ | C 33 - H 52 | σ* | C 14 - C 34 | 4.45 | 1.07 | 0.062 |
| σ | C 33 - H 52 | σ* | C 31 - C 32 | 4.92 | 1.05 | 0.064 |
| σ | C 33 - H 52 | σ* | C 32 - C 33 | 1.10 | 1.15 | 0.032 |
| σ | C 34 - C 35 | σ* | C 13 - C 14 | 3.08 | 1.28 | 0.056 |
| σ | C 34 - C 35 | σ* | C 14 - C 34 | 4.07 | 1.25 | 0.064 |
| σ | C 34 - C 35 | σ* | C 32 - C 33 | 1.73 | 1.33 | 0.043 |
| σ | C 34 - C 35 | σ* | C 33 - C 34 | 3.48 | 1.23 | 0.058 |
| σ | C 34 - C 35 | σ* | C 35 - C 36 | 3.09 | 1.27 | 0.056 |
| σ | C 34 - C 35 | σ* | C 35 - H 53 | 1.25 | 1.13 | 0.034 |
| σ | C 34 - C 35 | σ* | C 36 - C 37 | 3.45 | 1.27 | 0.059 |
| π | C 34 - C 35 | π* | C 3 - C 14 | 16.17 | 0.30 | 0.063 |
| π | C 34 - C 35 | π* | C 32 - C 33 | 17.22 | 0.30 | 0.066 |
| π | C 34 - C 35 | π* | C 36 - C 37 | 20.48 | 0.29 | 0.070 |
| σ | C 35 - C 36 | σ* | C 4 - C 5 | 3.17 | 1.27 | 0.057 |
| σ | C 35 - C 36 | σ* | C 4 - C 36 | 3.65 | 1.24 | 0.060 |
| σ | C 35 - C 36 | σ* | C 33 - C 34 | 3.83 | 1.21 | 0.061 |
| σ | C 35 - C 36 | σ* | C 34 - C 35 | 3.14 | 1.28 | 0.057 |
| σ | C 35 - C 36 | σ* | C 35 - H 53 | 1.03 | 1.12 | 0.030 |
| σ | C 35 - C 36 | σ* | C 36 - C 37 | 3.61 | 1.25 | 0.060 |
| σ | C 35 - C 36 | σ* | C 37 - C 38 | 2.16 | 1.28 | 0.047 |
| σ | C 35 - H 53 | σ* | C 4 - C 36 | 4.81 | 1.07 | 0.064 |
| σ | C 35 - H 53 | σ* | C 14 - C 34 | 5.23 | 1.07 | 0.067 |
| σ | C 35 - H 53 | σ* | C 34 - C 35 | 0.98 | 1.12 | 0.030 |
| σ | C 35 - H 53 | σ* | C 35 - C 36 | 0.72 | 1.09 | 0.025 |
| σ | C 36 - C 37 | σ* | C 3 - C 4 | 3.17 | 1.27 | 0.057 |
| σ | C 36 - C 37 | σ* | C 4 - C 36 | 3.65 | 1.24 | 0.060 |
| σ | C 36 - C 37 | σ* | C 34 - C 35 | 2.16 | 1.28 | 0.047 |
| σ | C 36 - C 37 | σ* | C 35 - C 36 | 3.61 | 1.25 | 0.060 |
| σ | C 36 - C 37 | σ* | C 37 - C 38 | 3.14 | 1.28 | 0.057 |
| σ | C 36 - C 37 | σ* | C 37 - H 54 | 1.03 | 1.12 | 0.030 |
| σ | C 36 - C 37 | σ* | C 38 - C 39 | 3.83 | 1.21 | 0.061 |
| π | C 36 - C 37 | π* | C 4 - C 5 | 17.66 | 0.29 | 0.064 |
| π | C 36 - C 37 | π* | C 34 - C 35 | 20.66 | 0.28 | 0.070 |
| σ | C 37 - C 38 | σ* | C 8 - C 9 | 3.08 | 1.28 | 0.056 |
| σ | C 37 - C 38 | σ* | C 8 - C 38 | 4.07 | 1.25 | 0.064 |
| σ | C 37 - C 38 | σ* | C 35 - C 36 | 3.45 | 1.27 | 0.059 |
| σ | C 37 - C 38 | σ* | C 36 - C 37 | 3.09 | 1.27 | 0.056 |
| σ | C 37 - C 38 | σ* | C 37 - H 54 | 1.25 | 1.13 | 0.034 |
| σ | C 37 - C 38 | σ* | C 38 - C 39 | 3.48 | 1.23 | 0.058 |
| σ | C 37 - C 38 | σ* | C 39 - C 40 | 1.73 | 1.33 | 0.043 |
| σ | C 37 - H 54 | σ* | C 4 - C 36 | 4.81 | 1.07 | 0.064 |
| σ | C 37 - H 54 | σ* | C 8 - C 38 | 5.23 | 1.07 | 0.067 |
| σ | C 37 - H 54 | σ* | C 36 - C 37 | 0.72 | 1.09 | 0.025 |
| σ | C 37 - H 54 | σ* | C 37 - C 38 | 0.98 | 1.12 | 0.030 |
| σ | C 38 - C 39 | σ* | C 5 - C 8 | 3.26 | 1.26 | 0.057 |
| σ | C 38 - C 39 | σ* | C 8 - C 38 | 3.07 | 1.22 | 0.055 |
| σ | C 38 - C 39 | σ* | C 36 - C 37 | 2.56 | 1.24 | 0.050 |
| σ | C 38 - C 39 | σ* | C 37 - C 38 | 3.63 | 1.27 | 0.061 |
| σ | C 38 - C 39 | σ* | C 39 - C 40 | 2.80 | 1.30 | 0.054 |
| σ | C 38 - C 39 | σ* | C 39 - H 55 | 0.75 | 1.11 | 0.026 |
| σ | C 38 - C 39 | σ* | C 40 - H 56 | 3.06 | 1.11 | 0.052 |
| σ | C 39 - C 40 | σ* | C 23 - C 24 | 3.21 | 1.29 | 0.058 |
| σ | C 39 - C 40 | σ* | C 23 - C 40 | 3.00 | 1.25 | 0.055 |
| σ | C 39 - C 40 | σ* | C 37 - C 38 | 3.20 | 1.32 | 0.058 |
| σ | C 39 - C 40 | σ* | C 38 - C 39 | 2.93 | 1.25 | 0.054 |
| σ | C 39 - C 40 | σ* | C 39 - H 55 | 1.53 | 1.16 | 0.038 |
| σ | C 39 - C 40 | σ* | C 40 - H 56 | 1.52 | 1.16 | 0.038 |
| π | C 39 - C 40 | π* | C 23 - C 24 | 15.82 | 0.30 | 0.065 |
| σ | C 39 - H 55 | σ* | C 8 - C 38 | 4.45 | 1.07 | 0.062 |
| σ | C 39 - H 55 | σ* | C 23 - C 40 | 4.92 | 1.05 | 0.064 |
| σ | C 39 - H 55 | σ* | C 39 - C 40 | 1.10 | 1.15 | 0.032 |
| σ | C 40 - H 56 | σ* | C 9 - C 23 | 4.46 | 1.09 | 0.062 |
| σ | C 40 - H 56 | σ* | C 38 - C 39 | 4.92 | 1.05 | 0.064 |
| σ | C 40 - H 56 | σ* | C 39 - C 40 | 1.08 | 1.15 | 0.032 |
| LP ( 1) | C 19 | π* | C 7 - C 10 | 65.55 | 0.14 | 0.101 |
| LP ( 1) | C 19 | π* | C 17 - C 18 | 78.32 | 0.14 | 0.109 |
| LP ( 1) | C 19 | π* | C 20 - C 22 | 48.27 | 0.15 | 0.097 |

E(2) : means energy of hyper conjugative interaction (stabilization energy).

E(j)-E(i) : Energy difference between donor and acceptor i and j NBO orbitals.

F (i, j) is the Fock matrix element between i and j NBO orbitals.

Table S6: Second order perturbation theory analysis of Fock matrix in NBO basis of C_40_F_16_ using B3LYP/cc-pVDZ

| C_40_F_16_/B3LYP/cc-pVDZ | |  |  | E(2) | E(j)-E(i) | F (i, j) |
| --- | --- | --- | --- | --- | --- | --- |
| Donor NBO (i) | Types | Acceptor NBO (j) | Types | kcal/mol | a.u. | a.u. |
| σ | C 1 - C 2 | σ* | C 1 - C 6 | 4.12 | 1.26 | 0.064 |
| σ | C 1 - C 2 | σ* | C 1 - C 17 | 3.44 | 1.22 | 0.058 |
| σ | C 1 - C 2 | σ* | C 2 - C 3 | 3.74 | 1.24 | 0.061 |
| σ | C 1 - C 2 | σ* | C 2 - C 11 | 4.08 | 1.27 | 0.064 |
| σ | C 1 - C 2 | σ* | C 3 - C 14 | 2.94 | 1.27 | 0.055 |
| σ | C 1 - C 2 | σ* | C 6 - C 7 | 3.13 | 1.27 | 0.056 |
| σ | C 1 - C 2 | σ* | C 11 - C 12 | 3.15 | 1.25 | 0.056 |
| σ | C 1 - C 2 | σ* | C 17 - C 18 | 2.64 | 1.23 | 0.051 |
| σ | C 1 - C 6 | σ* | C 1 - C 2 | 4.12 | 1.26 | 0.064 |
| σ | C 1 - C 6 | σ* | C 1 - C 17 | 3.44 | 1.22 | 0.058 |
| σ | C 1 - C 6 | σ* | C 2 - C 11 | 3.13 | 1.27 | 0.056 |
| σ | C 1 - C 6 | σ* | C 5 - C 6 | 3.74 | 1.24 | 0.061 |
| σ | C 1 - C 6 | σ* | C 5 - C 8 | 2.94 | 1.27 | 0.055 |
| σ | C 1 - C 6 | σ* | C 6 - C 7 | 4.08 | 1.27 | 0.064 |
| σ | C 1 - C 6 | σ* | C 7 - C 10 | 3.15 | 1.25 | 0.056 |
| σ | C 1 - C 6 | σ* | C 15 - C 17 | 2.64 | 1.23 | 0.051 |
| σ | C 1 - C 17 | σ* | C 1 - C 2 | 3.50 | 1.26 | 0.059 |
| σ | C 1 - C 17 | σ* | C 1 - C 6 | 3.50 | 1.26 | 0.059 |
| σ | C 1 - C 17 | σ* | C 2 - C 3 | 3.24 | 1.24 | 0.057 |
| σ | C 1 - C 17 | σ* | C 5 - C 6 | 3.24 | 1.24 | 0.057 |
| σ | C 1 - C 17 | σ* | C 15 - C 17 | 3.28 | 1.23 | 0.057 |
| σ | C 1 - C 17 | σ* | C 15 - F 55 | 3.78 | 0.94 | 0.054 |
| σ | C 1 - C 17 | σ* | C 17 - C 18 | 3.28 | 1.23 | 0.057 |
| σ | C 1 - C 17 | σ* | C 18 - F 41 | 3.78 | 0.94 | 0.054 |
| π | C 1 - C 17 | π* | C 2 - C 11 | 17.84 | 0.28 | 0.064 |
| π | C 1 - C 17 | π* | C 6 - C 7 | 17.84 | 0.28 | 0.064 |
| π | C 1 - C 17 | π* | C 15 - C 16 | 22.03 | 0.27 | 0.070 |
| π | C 1 - C 17 | π* | C 18 - C 19 | 22.03 | 0.27 | 0.070 |
| σ | C 2 - C 3 | σ* | C 1 - C 2 | 3.77 | 1.25 | 0.061 |
| σ | C 2 - C 3 | σ* | C 1 - C 17 | 3.20 | 1.21 | 0.056 |
| σ | C 2 - C 3 | σ* | C 2 - C 11 | 3.84 | 1.25 | 0.062 |
| σ | C 2 - C 3 | σ* | C 3 - C 4 | 3.77 | 1.25 | 0.061 |
| σ | C 2 - C 3 | σ* | C 3 - C 14 | 3.84 | 1.25 | 0.062 |
| σ | C 2 - C 3 | σ* | C 4 - C 36 | 3.20 | 1.21 | 0.056 |
| σ | C 2 - C 3 | σ* | C 11 - C 16 | 3.25 | 1.21 | 0.056 |
| σ | C 2 - C 3 | σ* | C 14 - C 34 | 3.25 | 1.21 | 0.056 |
| σ | C 2 - C 11 | σ* | C 1 - C 2 | 4.06 | 1.26 | 0.064 |
| σ | C 2 - C 11 | σ* | C 1 - C 6 | 3.07 | 1.26 | 0.056 |
| σ | C 2 - C 11 | σ* | C 2 - C 3 | 3.83 | 1.25 | 0.062 |
| σ | C 2 - C 11 | σ* | C 3 - C 4 | 2.92 | 1.26 | 0.054 |
| σ | C 2 - C 11 | σ* | C 11 - C 12 | 4.14 | 1.26 | 0.064 |
| σ | C 2 - C 11 | σ* | C 11 - C 16 | 3.51 | 1.22 | 0.059 |
| σ | C 2 - C 11 | σ* | C 12 - C 28 | 3.14 | 1.23 | 0.056 |
| σ | C 2 - C 11 | σ* | C 16 - C 26 | 2.64 | 1.21 | 0.050 |
| π | C 2 - C 11 | π* | C 1 - C 17 | 20.60 | 0.27 | 0.069 |
| π | C 2 - C 11 | π* | C 3 - C 14 | 18.16 | 0.28 | 0.064 |
| π | C 2 - C 11 | π* | C 12 - C 28 | 22.30 | 0.27 | 0.071 |

Table S6: Continued

| C_40_F_16_/B3LYP/cc-pVDZ | | |  | E(2) | E(j)-E(i) | F (i, j) |
| --- | --- | --- | --- | --- | --- | --- |
| Donor NBO (i) | Types | Acceptor NBO (j) | Types | kcal/mol | a.u. | a.u. |
| π | C 2 - C 11 | π* | C 15 - C 16 | 17.47 | 0.27 | 0.062 |
| σ | C 3 - C 4 | σ* | C 2 - C 3 | 3.74 | 1.24 | 0.061 |
| σ | C 3 - C 4 | σ* | C 2 - C 11 | 2.94 | 1.27 | 0.055 |
| σ | C 3 - C 4 | σ* | C 3 - C 14 | 4.08 | 1.27 | 0.064 |
| σ | C 3 - C 4 | σ* | C 4 - C 5 | 4.12 | 1.26 | 0.064 |
| σ | C 3 - C 4 | σ* | C 4 - C 36 | 3.44 | 1.22 | 0.058 |
| σ | C 3 - C 4 | σ* | C 5 - C 8 | 3.13 | 1.27 | 0.056 |
| σ | C 3 - C 4 | σ* | C 13 - C 14 | 3.15 | 1.25 | 0.056 |
| σ | C 3 - C 4 | σ* | C 36 - C 37 | 2.64 | 1.23 | 0.051 |
| σ | C 3 - C 14 | σ* | C 1 - C 2 | 2.92 | 1.26 | 0.054 |
| σ | C 3 - C 14 | σ* | C 2 - C 3 | 3.83 | 1.25 | 0.062 |
| σ | C 3 - C 14 | σ* | C 3 - C 4 | 4.06 | 1.26 | 0.064 |
| σ | C 3 - C 14 | σ* | C 4 - C 5 | 3.07 | 1.26 | 0.056 |
| σ | C 3 - C 14 | σ* | C 13 - C 14 | 4.14 | 1.26 | 0.064 |
| σ | C 3 - C 14 | σ* | C 13 - C 31 | 3.14 | 1.23 | 0.056 |
| σ | C 3 - C 14 | σ* | C 14 - C 34 | 3.51 | 1.22 | 0.059 |
| σ | C 3 - C 14 | σ* | C 33 - C 34 | 2.64 | 1.21 | 0.050 |
| π | C 3 - C 14 | π* | C 2 - C 11 | 18.16 | 0.28 | 0.064 |
| π | C 3 - C 14 | π* | C 4 - C 5 | 21.60 | 0.28 | 0.070 |
| π | C 3 - C 14 | π* | C 13 - C 31 | 22.30 | 0.27 | 0.071 |
| π | C 3 - C 14 | π* | C 34 - C 35 | 17.47 | 0.27 | 0.062 |
| σ | C 4 - C 5 | σ* | C 3 - C 4 | 4.12 | 1.26 | 0.064 |
| σ | C 4 - C 5 | σ* | C 3 - C 14 | 3.13 | 1.27 | 0.056 |
| σ | C 4 - C 5 | σ* | C 4 - C 36 | 3.44 | 1.22 | 0.058 |
| σ | C 4 - C 5 | σ* | C 5 - C 6 | 3.74 | 1.24 | 0.061 |
| σ | C 4 - C 5 | σ* | C 5 - C 8 | 4.08 | 1.27 | 0.064 |
| σ | C 4 - C 5 | σ* | C 6 - C 7 | 2.94 | 1.27 | 0.055 |
| σ | C 4 - C 5 | σ* | C 8 - C 9 | 3.15 | 1.25 | 0.056 |
| σ | C 4 - C 5 | σ* | C 35 - C 36 | 2.64 | 1.23 | 0.051 |
| π | C 4 - C 5 | LP ( 1) | C 36 | 50.82 | 0.13 | 0.086 |
| π | C 4 - C 5 | π* | C 3 - C 14 | 21.86 | 0.28 | 0.070 |
| π | C 4 - C 5 | π* | C 6 - C 7 | 20.62 | 0.28 | 0.068 |
| π | C 4 - C 5 | π* | C 8 - C 9 | 22.68 | 0.28 | 0.071 |
| σ | C 4 - C 36 | σ* | C 2 - C 3 | 3.24 | 1.24 | 0.057 |
| σ | C 4 - C 36 | σ* | C 3 - C 4 | 3.50 | 1.26 | 0.059 |
| σ | C 4 - C 36 | σ* | C 4 - C 5 | 3.50 | 1.26 | 0.059 |
| σ | C 4 - C 36 | σ* | C 5 - C 6 | 3.24 | 1.24 | 0.057 |
| σ | C 4 - C 36 | σ* | C 35 - C 36 | 3.28 | 1.23 | 0.057 |
| σ | C 4 - C 36 | σ* | C 35 - F 49 | 3.78 | 0.94 | 0.054 |
| σ | C 4 - C 36 | σ* | C 36 - C 37 | 3.28 | 1.23 | 0.057 |
| σ | C 4 - C 36 | σ* | C 37 - F 48 | 3.78 | 0.94 | 0.054 |
| σ | C 5 - C 6 | σ* | C 1 - C 6 | 3.77 | 1.25 | 0.061 |
| σ | C 5 - C 6 | σ* | C 1 - C 17 | 3.20 | 1.21 | 0.056 |
| σ | C 5 - C 6 | σ* | C 4 - C 5 | 3.77 | 1.25 | 0.061 |
| σ | C 5 - C 6 | σ* | C 4 - C 36 | 3.20 | 1.21 | 0.056 |
| σ | C 5 - C 6 | σ* | C 5 - C 8 | 3.84 | 1.25 | 0.062 |
| σ | C 5 - C 6 | σ* | C 6 - C 7 | 3.84 | 1.25 | 0.062 |
| σ | C 5 - C 6 | σ* | C 7 - C 19 | 3.25 | 1.21 | 0.056 |
| σ | C 5 - C 6 | σ* | C 8 - C 38 | 3.25 | 1.21 | 0.056 |
| σ | C 5 - C 8 | σ* | C 1 - C 6 | 2.92 | 1.26 | 0.054 |
| σ | C 5 - C 8 | σ* | C 3 - C 4 | 3.07 | 1.26 | 0.056 |
| σ | C 5 - C 8 | σ* | C 4 - C 5 | 4.06 | 1.26 | 0.064 |

Table S6: Continued

| C_40_F_16_/B3LYP/cc-pVDZ | | |  | E(2) | E(j)-E(i) | F (i, j) |
| --- | --- | --- | --- | --- | --- | --- |
| Donor NBO (i) | Types | Acceptor NBO (j) | Types | kcal/mol | a.u. | a.u. |
| σ | C 5 - C 8 | σ* | C 5 - C 6 | 3.83 | 1.25 | 0.062 |
| σ | C 5 - C 8 | σ* | C 8 - C 9 | 4.14 | 1.26 | 0.064 |
| σ | C 5 - C 8 | σ* | C 8 - C 38 | 3.51 | 1.22 | 0.059 |
| σ | C 5 - C 8 | σ* | C 9 - C 23 | 3.14 | 1.23 | 0.056 |
| σ | C 5 - C 8 | σ* | C 38 - C 39 | 2.64 | 1.21 | 0.050 |
| σ | C 6 - C 7 | σ* | C 1 - C 2 | 3.07 | 1.26 | 0.056 |
| σ | C 6 - C 7 | σ* | C 1 - C 6 | 4.06 | 1.26 | 0.064 |
| σ | C 6 - C 7 | σ* | C 4 - C 5 | 2.92 | 1.26 | 0.054 |
| σ | C 6 - C 7 | σ* | C 5 - C 6 | 3.83 | 1.25 | 0.062 |
| σ | C 6 - C 7 | σ* | C 7 - C 10 | 4.14 | 1.26 | 0.064 |
| σ | C 6 - C 7 | σ* | C 7 - C 19 | 3.51 | 1.22 | 0.059 |
| σ | C 6 - C 7 | σ* | C 10 - C 21 | 3.14 | 1.23 | 0.056 |
| σ | C 6 - C 7 | σ* | C 19 - C 20 | 2.64 | 1.21 | 0.050 |
| π | C 6 - C 7 | π* | C 1 - C 17 | 20.60 | 0.27 | 0.069 |
| π | C 6 - C 7 | π* | C 4 - C 5 | 19.90 | 0.28 | 0.068 |
| π | C 6 - C 7 | π* | C 10 - C 21 | 22.30 | 0.27 | 0.071 |
| π | C 6 - C 7 | π* | C 18 - C 19 | 17.47 | 0.27 | 0.062 |
| σ | C 7 - C 10 | σ* | C 1 - C 6 | 3.20 | 1.26 | 0.057 |
| σ | C 7 - C 10 | σ* | C 6 - C 7 | 4.16 | 1.27 | 0.065 |
| σ | C 7 - C 10 | σ* | C 7 - C 19 | 3.32 | 1.22 | 0.057 |
| σ | C 7 - C 10 | σ* | C 9 - C 10 | 4.00 | 1.25 | 0.063 |
| σ | C 7 - C 10 | σ* | C 9 - C 23 | 3.09 | 1.23 | 0.055 |
| σ | C 7 - C 10 | σ* | C 10 - C 21 | 3.58 | 1.23 | 0.059 |
| σ | C 7 - C 10 | σ* | C 18 - C 19 | 2.47 | 1.27 | 0.050 |
| σ | C 7 - C 10 | σ* | C 21 - C 25 | 2.61 | 1.24 | 0.051 |
| σ | C 7 - C 19 | σ* | C 5 - C 6 | 3.29 | 1.24 | 0.057 |
| σ | C 7 - C 19 | σ* | C 6 - C 7 | 3.59 | 1.26 | 0.060 |
| σ | C 7 - C 19 | σ* | C 7 - C 10 | 3.36 | 1.25 | 0.058 |
| σ | C 7 - C 19 | σ* | C 9 - C 10 | 3.15 | 1.24 | 0.056 |
| σ | C 7 - C 19 | σ* | C 18 - C 19 | 3.71 | 1.26 | 0.061 |
| σ | C 7 - C 19 | σ* | C 18 - F 41 | 4.04 | 0.94 | 0.055 |
| σ | C 7 - C 19 | σ* | C 19 - C 20 | 2.74 | 1.20 | 0.051 |
| σ | C 7 - C 19 | σ* | C 20 - F 42 | 3.70 | 0.94 | 0.053 |
| σ | C 8 - C 9 | σ* | C 4 - C 5 | 3.20 | 1.26 | 0.057 |
| σ | C 8 - C 9 | σ* | C 5 - C 8 | 4.16 | 1.27 | 0.065 |
| σ | C 8 - C 9 | σ* | C 8 - C 38 | 3.32 | 1.22 | 0.057 |
| σ | C 8 - C 9 | σ* | C 9 - C 10 | 4.00 | 1.25 | 0.063 |
| σ | C 8 - C 9 | σ* | C 9 - C 23 | 3.58 | 1.23 | 0.059 |
| σ | C 8 - C 9 | σ* | C 10 - C 21 | 3.09 | 1.23 | 0.055 |
| σ | C 8 - C 9 | σ* | C 23 - C 24 | 2.61 | 1.24 | 0.051 |
| σ | C 8 - C 9 | σ* | C 37 - C 38 | 2.47 | 1.27 | 0.050 |
| π | C 8 - C 9 | π* | C 4 - C 5 | 21.80 | 0.28 | 0.070 |
| π | C 8 - C 9 | π* | C 10 - C 21 | 22.30 | 0.27 | 0.071 |
| π | C 8 - C 9 | π* | C 37 - C 38 | 19.01 | 0.27 | 0.065 |
| σ | C 8 - C 38 | σ* | C 5 - C 6 | 3.29 | 1.24 | 0.057 |
| σ | C 8 - C 38 | σ* | C 5 - C 8 | 3.59 | 1.26 | 0.060 |
| σ | C 8 - C 38 | σ* | C 8 - C 9 | 3.36 | 1.25 | 0.058 |
| σ | C 8 - C 38 | σ* | C 9 - C 10 | 3.15 | 1.24 | 0.056 |
| σ | C 8 - C 38 | σ* | C 37 - C 38 | 3.71 | 1.26 | 0.061 |
| σ | C 8 - C 38 | σ* | C 37 - F 48 | 4.04 | 0.94 | 0.055 |

Table S6: Continued

| C_40_F_16_/B3LYP/cc-pVDZ | | |  | E(2) | E(j)-E(i) | F (i, j) |
| --- | --- | --- | --- | --- | --- | --- |
| Donor NBO (i) | Types | Acceptor NBO (j) | Types | kcal/mol | a.u. | a.u. |
| σ | C 8 - C 38 | σ* | C 38 - C 39 | 2.74 | 1.20 | 0.051 |
| σ | C 8 - C 38 | σ* | C 39 - F 47 | 3.70 | 0.94 | 0.053 |
| σ | C 9 - C 10 | σ* | C 7 - C 10 | 4.04 | 1.25 | 0.064 |
| σ | C 9 - C 10 | σ* | C 7 - C 19 | 3.16 | 1.22 | 0.056 |
| σ | C 9 - C 10 | σ* | C 8 - C 9 | 4.04 | 1.25 | 0.064 |
| σ | C 9 - C 10 | σ* | C 8 - C 38 | 3.16 | 1.22 | 0.056 |
| σ | C 9 - C 10 | σ* | C 9 - C 23 | 3.52 | 1.23 | 0.059 |
| σ | C 9 - C 10 | σ* | C 10 - C 21 | 3.52 | 1.23 | 0.059 |
| σ | C 9 - C 10 | σ* | C 21 - C 22 | 2.69 | 1.21 | 0.051 |
| σ | C 9 - C 10 | σ* | C 23 - C 40 | 2.69 | 1.21 | 0.051 |
| σ | C 9 - C 23 | σ* | C 5 - C 8 | 3.06 | 1.27 | 0.056 |
| σ | C 9 - C 23 | σ* | C 7 - C 10 | 3.09 | 1.26 | 0.056 |
| σ | C 9 - C 23 | σ* | C 8 - C 9 | 3.60 | 1.26 | 0.060 |
| σ | C 9 - C 23 | σ* | C 9 - C 10 | 3.56 | 1.25 | 0.060 |
| σ | C 9 - C 23 | σ* | C 23 - C 24 | 3.49 | 1.24 | 0.059 |
| σ | C 9 - C 23 | σ* | C 23 - C 40 | 3.01 | 1.21 | 0.054 |
| σ | C 9 - C 23 | σ* | C 24 - F 45 | 4.16 | 0.95 | 0.056 |
| σ | C 9 - C 23 | σ* | C 40 - F 46 | 3.61 | 0.95 | 0.053 |
| σ | C 10 - C 21 | σ* | C 6 - C 7 | 3.06 | 1.27 | 0.056 |
| σ | C 10 - C 21 | σ* | C 7 - C 10 | 3.60 | 1.26 | 0.060 |
| σ | C 10 - C 21 | σ* | C 8 - C 9 | 3.09 | 1.26 | 0.056 |
| σ | C 10 - C 21 | σ* | C 9 - C 10 | 3.56 | 1.25 | 0.060 |
| σ | C 10 - C 21 | σ* | C 21 - C 22 | 3.01 | 1.21 | 0.054 |
| σ | C 10 - C 21 | σ* | C 21 - C 25 | 3.49 | 1.24 | 0.059 |
| σ | C 10 - C 21 | σ* | C 22 - F 43 | 3.61 | 0.95 | 0.053 |
| σ | C 10 - C 21 | σ* | C 25 - F 44 | 4.16 | 0.95 | 0.056 |
| π | C 10 - C 21 | π* | C 6 - C 7 | 19.86 | 0.29 | 0.068 |
| π | C 10 - C 21 | π* | C 8 - C 9 | 19.33 | 0.28 | 0.067 |
| π | C 10 - C 21 | π* | C 20 - C 22 | 17.40 | 0.28 | 0.065 |
| π | C 10 - C 21 | π* | C 24 - C 25 | 22.02 | 0.27 | 0.069 |
| σ | C 11 - C 12 | σ* | C 1 - C 2 | 3.20 | 1.26 | 0.057 |
| σ | C 11 - C 12 | σ* | C 2 - C 11 | 4.16 | 1.27 | 0.065 |
| σ | C 11 - C 12 | σ* | C 11 - C 16 | 3.32 | 1.22 | 0.057 |
| σ | C 11 - C 12 | σ* | C 12 - C 13 | 4.00 | 1.25 | 0.063 |
| σ | C 11 - C 12 | σ* | C 12 - C 28 | 3.58 | 1.23 | 0.059 |
| σ | C 11 - C 12 | σ* | C 13 - C 31 | 3.09 | 1.23 | 0.055 |
| σ | C 11 - C 12 | σ* | C 15 - C 16 | 2.47 | 1.27 | 0.050 |
| σ | C 11 - C 12 | σ* | C 28 - C 29 | 2.61 | 1.24 | 0.051 |
| σ | C 11 - C 16 | σ* | C 2 - C 3 | 3.29 | 1.24 | 0.057 |
| σ | C 11 - C 16 | σ* | C 2 - C 11 | 3.59 | 1.26 | 0.060 |
| σ | C 11 - C 16 | σ* | C 11 - C 12 | 3.36 | 1.25 | 0.058 |
| σ | C 11 - C 16 | σ* | C 12 - C 13 | 3.15 | 1.24 | 0.056 |
| σ | C 11 - C 16 | σ* | C 15 - C 16 | 3.71 | 1.26 | 0.061 |
| σ | C 11 - C 16 | σ* | C 15 - F 55 | 4.04 | 0.94 | 0.055 |
| σ | C 11 - C 16 | σ* | C 16 - C 26 | 2.74 | 1.20 | 0.051 |
| σ | C 11 - C 16 | σ* | C 26 - F 54 | 3.70 | 0.94 | 0.053 |
| σ | C 12 - C 13 | σ* | C 11 - C 12 | 4.04 | 1.25 | 0.064 |
| σ | C 12 - C 13 | σ* | C 11 - C 16 | 3.16 | 1.22 | 0.056 |
| σ | C 12 - C 13 | σ* | C 12 - C 28 | 3.52 | 1.23 | 0.059 |
| σ | C 12 - C 13 | σ* | C 13 - C 14 | 4.04 | 1.25 | 0.064 |
| σ | C 12 - C 13 | σ* | C 13 - C 31 | 3.52 | 1.23 | 0.059 |
| σ | C 12 - C 13 | σ* | C 14 - C 34 | 3.16 | 1.22 | 0.056 |

Table S6: Continued

| C_40_F_16_/B3LYP/cc-pVDZ | | |  | E(2) | E(j)-E(i) | F (i, j) |
| --- | --- | --- | --- | --- | --- | --- |
| Donor NBO (i) | Types | Acceptor NBO (j) | Types | kcal/mol | a.u. | a.u. |
| σ | C 12 - C 13 | σ* | C 27 - C 28 | 2.69 | 1.21 | 0.051 |
| σ | C 12 - C 13 | σ* | C 31 - C 32 | 2.69 | 1.21 | 0.051 |
| σ | C 12 - C 28 | σ* | C 2 - C 11 | 3.06 | 1.27 | 0.056 |
| σ | C 12 - C 28 | σ* | C 11 - C 12 | 3.60 | 1.26 | 0.060 |
| σ | C 12 - C 28 | σ* | C 12 - C 13 | 3.56 | 1.25 | 0.060 |
| σ | C 12 - C 28 | σ* | C 13 - C 14 | 3.09 | 1.26 | 0.056 |
| σ | C 12 - C 28 | σ* | C 27 - C 28 | 3.01 | 1.21 | 0.054 |
| σ | C 12 - C 28 | σ* | C 27 - F 56 | 3.61 | 0.95 | 0.053 |
| σ | C 12 - C 28 | σ* | C 28 - C 29 | 3.49 | 1.24 | 0.059 |
| σ | C 12 - C 28 | σ* | C 29 - F 53 | 4.16 | 0.95 | 0.056 |
| π | C 12 - C 28 | π* | C 2 - C 11 | 19.86 | 0.29 | 0.068 |
| π | C 12 - C 28 | π* | C 13 - C 31 | 18.79 | 0.28 | 0.066 |
| π | C 12 - C 28 | π* | C 26 - C 27 | 17.40 | 0.28 | 0.065 |
| π | C 12 - C 28 | π* | C 29 - C 30 | 22.02 | 0.27 | 0.069 |
| σ | C 13 - C 14 | σ* | C 3 - C 4 | 3.20 | 1.26 | 0.057 |
| σ | C 13 - C 14 | σ* | C 3 - C 14 | 4.16 | 1.27 | 0.065 |
| σ | C 13 - C 14 | σ* | C 12 - C 13 | 4.00 | 1.25 | 0.063 |
| σ | C 13 - C 14 | σ* | C 12 - C 28 | 3.09 | 1.23 | 0.055 |
| σ | C 13 - C 14 | σ* | C 13 - C 31 | 3.58 | 1.23 | 0.059 |
| σ | C 13 - C 14 | σ* | C 14 - C 34 | 3.32 | 1.22 | 0.057 |
| σ | C 13 - C 14 | σ* | C 30 - C 31 | 2.61 | 1.24 | 0.051 |
| σ | C 13 - C 14 | σ* | C 34 - C 35 | 2.47 | 1.27 | 0.050 |
| σ | C 13 - C 31 | σ* | C 3 - C 14 | 3.06 | 1.27 | 0.056 |
| σ | C 13 - C 31 | σ* | C 11 - C 12 | 3.09 | 1.26 | 0.056 |
| σ | C 13 - C 31 | σ* | C 12 - C 13 | 3.56 | 1.25 | 0.060 |
| σ | C 13 - C 31 | σ* | C 13 - C 14 | 3.60 | 1.26 | 0.060 |
| σ | C 13 - C 31 | σ* | C 30 - C 31 | 3.49 | 1.24 | 0.059 |
| σ | C 13 - C 31 | σ* | C 30 - F 52 | 4.16 | 0.95 | 0.056 |
| σ | C 13 - C 31 | σ* | C 31 - C 32 | 3.01 | 1.21 | 0.054 |
| σ | C 13 - C 31 | σ* | C 32 - F 51 | 3.61 | 0.95 | 0.053 |
| π | C 13 - C 31 | π* | C 3 - C 14 | 19.86 | 0.29 | 0.068 |
| π | C 13 - C 31 | π* | C 12 - C 28 | 18.79 | 0.28 | 0.066 |
| π | C 13 - C 31 | π* | C 29 - C 30 | 22.02 | 0.27 | 0.069 |
| π | C 13 - C 31 | π* | C 32 - C 33 | 17.40 | 0.28 | 0.065 |
| σ | C 14 - C 34 | σ* | C 2 - C 3 | 3.29 | 1.24 | 0.057 |
| σ | C 14 - C 34 | σ* | C 3 - C 14 | 3.59 | 1.26 | 0.060 |
| σ | C 14 - C 34 | σ* | C 12 - C 13 | 3.15 | 1.24 | 0.056 |
| σ | C 14 - C 34 | σ* | C 13 - C 14 | 3.36 | 1.25 | 0.058 |
| σ | C 14 - C 34 | σ* | C 33 - C 34 | 2.74 | 1.20 | 0.051 |
| σ | C 14 - C 34 | σ* | C 33 - F 50 | 3.70 | 0.94 | 0.053 |
| σ | C 14 - C 34 | σ* | C 34 - C 35 | 3.71 | 1.26 | 0.061 |
| σ | C 14 - C 34 | σ* | C 35 - F 49 | 4.04 | 0.94 | 0.055 |
| σ | C 15 - C 16 | σ* | C 11 - C 12 | 3.02 | 1.30 | 0.056 |
| σ | C 15 - C 16 | σ* | C 11 - C 16 | 4.26 | 1.26 | 0.066 |
| σ | C 15 - C 16 | σ* | C 15 - C 17 | 3.82 | 1.28 | 0.062 |
| σ | C 15 - C 16 | σ* | C 16 - C 26 | 3.52 | 1.25 | 0.059 |
| σ | C 15 - C 16 | σ* | C 17 - C 18 | 3.11 | 1.28 | 0.056 |
| σ | C 15 - C 16 | σ* | C 26 - C 27 | 1.96 | 1.34 | 0.046 |
| π | C 15 - C 16 | π* | C 1 - C 17 | 15.38 | 0.30 | 0.064 |
| π | C 15 - C 16 | π* | C 2 - C 11 | 16.71 | 0.31 | 0.066 |
| π | C 15 - C 16 | π* | C 15 - C 16 | 0.90 | 0.30 | 0.015 |
| π | C 15 - C 16 | π* | C 26 - C 27 | 19.21 | 0.30 | 0.069 |
| σ | C 15 - C 17 | σ* | C 1 - C 6 | 3.06 | 1.29 | 0.056 |
| σ | C 15 - C 17 | σ* | C 1 - C 17 | 3.80 | 1.25 | 0.062 |

Table S6: Continued

| C_40_F_16_/B3LYP/cc-pVDZ | | |  | E(2) | E(j)-E(i) | F (i, j) |
| --- | --- | --- | --- | --- | --- | --- |
| Donor NBO (i) | Types | Acceptor NBO (j) | Types | kcal/mol | a.u. | a.u. |
| σ | C 15 - C 17 | σ* | C 15 - C 16 | 3.85 | 1.29 | 0.063 |
| σ | C 15 - C 17 | σ* | C 16 - C 26 | 3.44 | 1.23 | 0.058 |
| σ | C 15 - C 17 | σ* | C 17 - C 18 | 3.63 | 1.26 | 0.061 |
| σ | C 15 - C 17 | σ* | C 18 - C 19 | 2.50 | 1.29 | 0.051 |
| σ | C 15 - F 55 | σ* | C 1 - C 17 | 2.16 | 1.57 | 0.052 |
| σ | C 15 - F 55 | σ* | C 11 - C 16 | 2.37 | 1.57 | 0.055 |
| σ | C 16 - C 26 | σ* | C 2 - C 11 | 3.11 | 1.28 | 0.056 |
| σ | C 16 - C 26 | σ* | C 11 - C 16 | 3.19 | 1.24 | 0.056 |
| σ | C 16 - C 26 | σ* | C 15 - C 16 | 3.67 | 1.28 | 0.061 |
| σ | C 16 - C 26 | σ* | C 15 - C 17 | 2.95 | 1.25 | 0.054 |
| σ | C 16 - C 26 | σ* | C 26 - C 27 | 3.29 | 1.31 | 0.059 |
| σ | C 16 - C 26 | σ* | C 27 - F 56 | 3.69 | 0.97 | 0.053 |
| σ | C 17 - C 18 | σ* | C 1 - C 2 | 3.06 | 1.29 | 0.056 |
| σ | C 17 - C 18 | σ* | C 1 - C 17 | 3.80 | 1.25 | 0.062 |
| σ | C 17 - C 18 | σ* | C 15 - C 16 | 2.50 | 1.29 | 0.051 |
| σ | C 17 - C 18 | σ* | C 15 - C 17 | 3.63 | 1.26 | 0.061 |
| σ | C 17 - C 18 | σ* | C 18 - C 19 | 3.85 | 1.29 | 0.063 |
| σ | C 17 - C 18 | σ* | C 19 - C 20 | 3.44 | 1.23 | 0.058 |
| σ | C 18 - C 19 | σ* | C 7 - C 10 | 3.02 | 1.30 | 0.056 |
| σ | C 18 - C 19 | σ* | C 7 - C 19 | 4.26 | 1.26 | 0.066 |
| σ | C 18 - C 19 | σ* | C 15 - C 17 | 3.11 | 1.28 | 0.056 |
| σ | C 18 - C 19 | σ* | C 17 - C 18 | 3.82 | 1.28 | 0.062 |
| σ | C 18 - C 19 | σ* | C 19 - C 20 | 3.52 | 1.25 | 0.059 |
| σ | C 18 - C 19 | σ* | C 20 - C 22 | 1.96 | 1.34 | 0.046 |
| π | C 18 - C 19 | π* | C 1 - C 17 | 15.38 | 0.30 | 0.064 |
| π | C 18 - C 19 | π* | C 6 - C 7 | 16.71 | 0.31 | 0.066 |
| π | C 18 - C 19 | π* | C 18 - C 19 | 0.90 | 0.30 | 0.015 |
| π | C 18 - C 19 | π* | C 20 - C 22 | 19.21 | 0.30 | 0.069 |
| σ | C 18 - F 41 | σ* | C 1 - C 17 | 2.16 | 1.57 | 0.052 |
| σ | C 18 - F 41 | σ* | C 7 - C 19 | 2.37 | 1.57 | 0.055 |
| σ | C 19 - C 20 | σ* | C 6 - C 7 | 3.11 | 1.28 | 0.056 |
| σ | C 19 - C 20 | σ* | C 7 - C 19 | 3.19 | 1.24 | 0.056 |
| σ | C 19 - C 20 | σ* | C 17 - C 18 | 2.95 | 1.25 | 0.054 |
| σ | C 19 - C 20 | σ* | C 18 - C 19 | 3.67 | 1.28 | 0.061 |
| σ | C 19 - C 20 | σ* | C 20 - C 22 | 3.29 | 1.31 | 0.059 |
| σ | C 19 - C 20 | σ* | C 22 - F 43 | 3.69 | 0.97 | 0.053 |
| σ | C 20 - C 22 | σ* | C 18 - C 19 | 2.92 | 1.34 | 0.056 |
| σ | C 20 - C 22 | σ* | C 19 - C 20 | 3.68 | 1.27 | 0.061 |
| σ | C 20 - C 22 | σ* | C 21 - C 22 | 3.80 | 1.28 | 0.062 |
| σ | C 20 - C 22 | σ* | C 21 - C 25 | 2.94 | 1.31 | 0.056 |
| π | C 20 - C 22 | π* | C 10 - C 21 | 13.69 | 0.32 | 0.063 |
| π | C 20 - C 22 | π* | C 18 - C 19 | 16.16 | 0.32 | 0.067 |
| σ | C 20 - F 42 | σ* | C 7 - C 19 | 1.91 | 1.56 | 0.049 |
| σ | C 20 - F 42 | σ* | C 21 - C 22 | 2.29 | 1.55 | 0.054 |
| σ | C 21 - C 22 | σ* | C 9 - C 10 | 3.29 | 1.27 | 0.058 |
| σ | C 21 - C 22 | σ* | C 10 - C 21 | 3.53 | 1.25 | 0.059 |
| σ | C 21 - C 22 | σ* | C 20 - C 22 | 3.38 | 1.32 | 0.060 |
| σ | C 21 - C 22 | σ* | C 20 - F 42 | 3.57 | 0.97 | 0.053 |

Table S6: Continued

| C_40_F_16_/B3LYP/cc-pVDZ | | |  | E(2) | E(j)-E(i) | F (i, j) |
| --- | --- | --- | --- | --- | --- | --- |
| Donor NBO (i) | Types | Acceptor NBO (j) | Types | kcal/mol | a.u. | a.u. |
| σ | C 21 - C 22 | σ* | C 21 - C 25 | 3.35 | 1.26 | 0.058 |
| σ | C 21 - C 22 | σ* | C 24 - C 25 | 2.48 | 1.28 | 0.050 |
| σ | C 21 - C 25 | σ* | C 7 - C 10 | 3.22 | 1.29 | 0.058 |
| σ | C 21 - C 25 | σ* | C 10 - C 21 | 3.99 | 1.27 | 0.064 |
| σ | C 21 - C 25 | σ* | C 20 - C 22 | 2.10 | 1.33 | 0.047 |
| σ | C 21 - C 25 | σ* | C 21 - C 22 | 3.34 | 1.25 | 0.058 |
| σ | C 21 - C 25 | σ* | C 24 - C 25 | 3.33 | 1.29 | 0.059 |
| σ | C 21 - C 25 | σ* | C 24 - F 45 | 3.08 | 0.99 | 0.049 |
| σ | C 22 - F 43 | σ* | C 10 - C 21 | 1.95 | 1.57 | 0.050 |
| σ | C 22 - F 43 | σ* | C 19 - C 20 | 2.26 | 1.54 | 0.053 |
| σ | C 23 - C 24 | σ* | C 8 - C 9 | 3.22 | 1.29 | 0.058 |
| σ | C 23 - C 24 | σ* | C 9 - C 23 | 3.99 | 1.27 | 0.064 |
| σ | C 23 - C 24 | σ* | C 23 - C 40 | 3.34 | 1.25 | 0.058 |
| σ | C 23 - C 24 | σ* | C 24 - C 25 | 3.33 | 1.29 | 0.059 |
| σ | C 23 - C 24 | σ* | C 25 - F 44 | 3.08 | 0.99 | 0.049 |
| σ | C 23 - C 24 | σ* | C 39 - C 40 | 2.10 | 1.33 | 0.047 |
| σ | C 23 - C 40 | σ* | C 9 - C 10 | 3.29 | 1.27 | 0.058 |
| σ | C 23 - C 40 | σ* | C 9 - C 23 | 3.53 | 1.25 | 0.059 |
| σ | C 23 - C 40 | σ* | C 23 - C 24 | 3.35 | 1.26 | 0.058 |
| σ | C 23 - C 40 | σ* | C 24 - C 25 | 2.48 | 1.28 | 0.050 |
| σ | C 23 - C 40 | σ* | C 39 - C 40 | 3.38 | 1.32 | 0.060 |
| σ | C 23 - C 40 | σ* | C 39 - F 47 | 3.57 | 0.97 | 0.053 |
| σ | C 24 - C 25 | σ* | C 21 - C 22 | 3.39 | 1.26 | 0.059 |
| σ | C 24 - C 25 | σ* | C 21 - C 25 | 3.86 | 1.30 | 0.063 |
| σ | C 24 - C 25 | σ* | C 23 - C 24 | 3.86 | 1.30 | 0.063 |
| σ | C 24 - C 25 | σ* | C 23 - C 40 | 3.39 | 1.26 | 0.059 |
| π | C 24 - C 25 | π* | C 10 - C 21 | 17.13 | 0.31 | 0.068 |
| σ | C 24 - F 45 | σ* | C 9 - C 23 | 2.09 | 1.58 | 0.052 |
| σ | C 24 - F 45 | σ* | C 21 - C 25 | 2.06 | 1.59 | 0.051 |
| σ | C 25 - F 44 | σ* | C 10 - C 21 | 2.09 | 1.58 | 0.052 |
| σ | C 25 - F 44 | σ* | C 23 - C 24 | 2.06 | 1.59 | 0.051 |
| σ | C 26 - C 27 | σ* | C 15 - C 16 | 2.92 | 1.34 | 0.056 |
| σ | C 26 - C 27 | σ* | C 16 - C 26 | 3.68 | 1.27 | 0.061 |
| σ | C 26 - C 27 | σ* | C 27 - C 28 | 3.80 | 1.28 | 0.062 |
| σ | C 26 - C 27 | σ* | C 28 - C 29 | 2.94 | 1.31 | 0.056 |
| π | C 26 - C 27 | π* | C 12 - C 28 | 13.69 | 0.32 | 0.063 |
| π | C 26 - C 27 | π* | C 15 - C 16 | 16.16 | 0.32 | 0.067 |
| σ | C 26 - F 54 | σ* | C 11 - C 16 | 1.91 | 1.56 | 0.049 |
| σ | C 26 - F 54 | σ* | C 27 - C 28 | 2.29 | 1.55 | 0.054 |
| σ | C 27 - C 28 | σ* | C 12 - C 13 | 3.29 | 1.27 | 0.058 |
| σ | C 27 - C 28 | σ* | C 12 - C 28 | 3.53 | 1.25 | 0.059 |
| σ | C 27 - C 28 | σ* | C 26 - C 27 | 3.38 | 1.32 | 0.060 |
| σ | C 27 - C 28 | σ* | C 26 - F 54 | 3.57 | 0.97 | 0.053 |
| σ | C 27 - C 28 | σ* | C 28 - C 29 | 3.35 | 1.26 | 0.058 |
| σ | C 27 - C 28 | σ* | C 29 - C 30 | 2.48 | 1.28 | 0.050 |
| σ | C 27 - F 56 | σ* | C 12 - C 28 | 1.95 | 1.57 | 0.050 |
| σ | C 27 - F 56 | σ* | C 16 - C 26 | 2.26 | 1.54 | 0.053 |
| σ | C 28 - C 29 | σ* | C 11 - C 12 | 3.22 | 1.29 | 0.058 |
| σ | C 28 - C 29 | σ* | C 12 - C 28 | 3.99 | 1.27 | 0.064 |
| σ | C 28 - C 29 | σ* | C 26 - C 27 | 2.10 | 1.33 | 0.047 |
| σ | C 28 - C 29 | σ* | C 27 - C 28 | 3.34 | 1.25 | 0.058 |
| σ | C 28 - C 29 | σ* | C 29 - C 30 | 3.33 | 1.29 | 0.059 |
| σ | C 28 - C 29 | σ* | C 30 - F 52 | 3.08 | 0.99 | 0.049 |
| σ | C 29 - C 30 | σ* | C 27 - C 28 | 3.39 | 1.26 | 0.059 |
| σ | C 29 - C 30 | σ* | C 28 - C 29 | 3.86 | 1.30 | 0.063 |
| σ | C 29 - C 30 | σ* | C 30 - C 31 | 3.86 | 1.30 | 0.063 |
| σ | C 29 - C 30 | σ* | C 31 - C 32 | 3.39 | 1.26 | 0.059 |
| π | C 29 - C 30 | π* | C 12 - C 28 | 17.13 | 0.31 | 0.068 |
| π | C 29 - C 30 | π* | C 13 - C 31 | 17.13 | 0.31 | 0.068 |
| σ | C 29 - F 53 | σ* | C 12 - C 28 | 2.09 | 1.58 | 0.052 |
| σ | C 29 - F 53 | σ* | C 30 - C 31 | 2.06 | 1.59 | 0.051 |
| σ | C 30 - C 31 | σ* | C 13 - C 14 | 3.22 | 1.29 | 0.058 |

Table S6: Continued

| C_40_F_16_/B3LYP/cc-pVDZ | | |  | E(2) | E(j)-E(i) | F (i, j) |
| --- | --- | --- | --- | --- | --- | --- |
| Donor NBO (i) | Types | Acceptor NBO (j) | Types | kcal/mol | a.u. | a.u. |
| π | C 29 - C 30 | π* | C 12 - C 28 | 17.13 | 0.31 | 0.068 |
| π | C 29 - C 30 | π* | C 13 - C 31 | 17.13 | 0.31 | 0.068 |
| σ | C 29 - F 53 | σ* | C 12 - C 28 | 2.09 | 1.58 | 0.052 |
| σ | C 29 - F 53 | σ* | C 30 - C 31 | 2.06 | 1.59 | 0.051 |
| σ | C 30 - C 31 | σ* | C 13 - C 14 | 3.22 | 1.29 | 0.058 |
| σ | C 30 - C 31 | σ* | C 13 - C 31 | 3.99 | 1.27 | 0.064 |
| σ | C 30 - C 31 | σ* | C 29 - C 30 | 3.33 | 1.29 | 0.059 |
| σ | C 30 - C 31 | σ* | C 29 - F 53 | 3.08 | 0.99 | 0.049 |
| σ | C 30 - C 31 | σ* | C 31 - C 32 | 3.34 | 1.25 | 0.058 |
| σ | C 30 - C 31 | σ* | C 32 - C 33 | 2.10 | 1.33 | 0.047 |
| σ | C 30 - F 52 | σ* | C 13 - C 31 | 2.09 | 1.58 | 0.052 |
| σ | C 30 - F 52 | σ* | C 28 - C 29 | 2.06 | 1.59 | 0.051 |
| σ | C 31 - C 32 | σ* | C 12 - C 13 | 3.29 | 1.27 | 0.058 |
| σ | C 31 - C 32 | σ* | C 13 - C 31 | 3.53 | 1.25 | 0.059 |
| σ | C 31 - C 32 | σ* | C 29 - C 30 | 2.48 | 1.28 | 0.050 |
| σ | C 31 - C 32 | σ* | C 30 - C 31 | 3.35 | 1.26 | 0.058 |
| σ | C 31 - C 32 | σ* | C 32 - C 33 | 3.38 | 1.32 | 0.060 |
| σ | C 31 - C 32 | σ* | C 33 - F 50 | 3.57 | 0.97 | 0.053 |
| σ | C 32 - C 33 | σ* | C 30 - C 31 | 2.94 | 1.31 | 0.056 |
| σ | C 32 - C 33 | σ* | C 31 - C 32 | 3.80 | 1.28 | 0.062 |
| σ | C 32 - C 33 | σ* | C 33 - C 34 | 3.68 | 1.27 | 0.061 |
| σ | C 32 - C 33 | σ* | C 34 - C 35 | 2.92 | 1.34 | 0.056 |
| π | C 32 - C 33 | π* | C 13 - C 31 | 13.69 | 0.32 | 0.063 |
| π | C 32 - C 33 | π* | C 34 - C 35 | 16.16 | 0.32 | 0.067 |
| σ | C 32 - F 51 | σ* | C 13 - C 31 | 1.95 | 1.57 | 0.050 |
| σ | C 32 - F 51 | σ* | C 33 - C 34 | 2.26 | 1.54 | 0.053 |
| σ | C 33 - C 34 | σ* | C 3 - C 14 | 3.11 | 1.28 | 0.056 |
| σ | C 33 - C 34 | σ* | C 14 - C 34 | 3.19 | 1.24 | 0.056 |
| σ | C 33 - C 34 | σ* | C 32 - C 33 | 3.29 | 1.31 | 0.059 |
| σ | C 33 - C 34 | σ* | C 32 - F 51 | 3.69 | 0.97 | 0.053 |
| σ | C 33 - C 34 | σ* | C 34 - C 35 | 3.67 | 1.28 | 0.061 |
| σ | C 33 - C 34 | σ* | C 35 - C 36 | 2.95 | 1.25 | 0.054 |
| σ | C 33 - F 50 | σ* | C 14 - C 34 | 1.91 | 1.56 | 0.049 |
| σ | C 33 - F 50 | σ* | C 31 - C 32 | 2.29 | 1.55 | 0.054 |
| σ | C 34 - C 35 | σ* | C 13 - C 14 | 3.02 | 1.30 | 0.056 |
| σ | C 34 - C 35 | σ* | C 14 - C 34 | 4.26 | 1.26 | 0.066 |
| σ | C 34 - C 35 | σ* | C 32 - C 33 | 1.96 | 1.34 | 0.046 |
| σ | C 34 - C 35 | σ* | C 33 - C 34 | 3.52 | 1.25 | 0.059 |
| σ | C 34 - C 35 | σ* | C 35 - C 36 | 3.82 | 1.28 | 0.062 |
| σ | C 34 - C 35 | σ* | C 36 - C 37 | 3.11 | 1.28 | 0.056 |
| π | C 34 - C 35 | π* | C 3 - C 14 | 16.71 | 0.31 | 0.066 |
| π | C 34 - C 35 | π* | C 32 - C 33 | 19.21 | 0.30 | 0.069 |
| π | C 34 - C 35 | π* | C 34 - C 35 | 0.90 | 0.30 | 0.015 |
| σ | C 35 - C 36 | σ* | C 4 - C 5 | 3.06 | 1.29 | 0.056 |
| σ | C 35 - C 36 | σ* | C 4 - C 36 | 3.80 | 1.25 | 0.062 |
| σ | C 35 - C 36 | σ* | C 33 - C 34 | 3.44 | 1.23 | 0.058 |
| σ | C 35 - C 36 | σ* | C 34 - C 35 | 3.85 | 1.29 | 0.063 |
| σ | C 35 - C 36 | σ* | C 36 - C 37 | 3.63 | 1.26 | 0.061 |
| σ | C 35 - C 36 | σ* | C 37 - C 38 | 2.50 | 1.29 | 0.051 |
| σ | C 35 - F 49 | σ* | C 4 - C 36 | 2.16 | 1.57 | 0.052 |
| σ | C 35 - F 49 | σ* | C 14 - C 34 | 2.37 | 1.57 | 0.055 |
| σ | C 36 - C 37 | σ* | C 3 - C 4 | 3.06 | 1.29 | 0.056 |
| σ | C 36 - C 37 | σ* | C 4 - C 36 | 3.80 | 1.25 | 0.062 |
| σ | C 36 - C 37 | σ* | C 34 - C 35 | 2.50 | 1.29 | 0.051 |
| σ | C 36 - C 37 | σ* | C 35 - C 36 | 3.63 | 1.26 | 0.061 |
| σ | C 36 - C 37 | σ* | C 37 - C 38 | 3.85 | 1.29 | 0.063 |
| σ | C 36 - C 37 | σ* | C 38 - C 39 | 3.44 | 1.23 | 0.058 |
| σ | C 37 - C 38 | σ* | C 8 - C 9 | 3.02 | 1.30 | 0.056 |
| σ | C 37 - C 38 | σ* | C 8 - C 38 | 4.26 | 1.26 | 0.066 |
| σ | C 37 - C 38 | σ* | C 35 - C 36 | 3.11 | 1.28 | 0.056 |
| σ | C 37 - C 38 | σ* | C 36 - C 37 | 3.82 | 1.28 | 0.062 |
| σ | C 37 - C 38 | σ* | C 38 - C 39 | 3.52 | 1.25 | 0.059 |
| σ | C 37 - C 38 | σ* | C 39 - C 40 | 1.96 | 1.34 | 0.046 |
| π | C 37 - C 38 | π* | C 8 - C 9 | 18.28 | 0.30 | 0.069 |
| π | C 37 - C 38 | π* | C 37 - C 38 | 0.90 | 0.30 | 0.015 |
| π | C 37 - C 38 | π* | C 39 - C 40 | 19.21 | 0.30 | 0.069 |
| σ | C 37 - F 48 | σ* | C 4 - C 36 | 2.16 | 1.57 | 0.052 |
| σ | C 37 - F 48 | σ* | C 8 - C 38 | 2.37 | 1.57 | 0.055 |
| σ | C 38 - C 39 | σ* | C 5 - C 8 | 3.11 | 1.28 | 0.056 |
| σ | C 38 - C 39 | σ* | C 8 - C 38 | 3.19 | 1.24 | 0.056 |
| σ | C 38 - C 39 | σ* | C 36 - C 37 | 2.95 | 1.25 | 0.054 |
| σ | C 38 - C 39 | σ* | C 37 - C 38 | 3.67 | 1.28 | 0.061 |
| σ | C 38 - C 39 | σ* | C 39 - C 40 | 3.29 | 1.31 | 0.059 |
| σ | C 38 - C 39 | σ* | C 40 - F 46 | 3.69 | 0.97 | 0.053 |
| σ | C 39 - C 40 | σ* | C 23 - C 24 | 2.94 | 1.31 | 0.056 |
| σ | C 39 - C 40 | σ* | C 23 - C 40 | 3.80 | 1.28 | 0.062 |
| σ | C 39 - C 40 | σ* | C 37 - C 38 | 2.92 | 1.34 | 0.056 |
| σ | C 39 - C 40 | σ* | C 38 - C 39 | 3.68 | 1.27 | 0.061 |
| π | C 39 - C 40 | π* | C 37 - C 38 | 16.16 | 0.32 | 0.067 |
| σ | C 39 - F 47 | σ* | C 8 - C 38 | 1.91 | 1.56 | 0.049 |
| σ | C 39 - F 47 | σ* | C 23 - C 40 | 2.29 | 1.55 | 0.054 |
| σ | C 40 - F 46 | σ* | C 9 - C 23 | 1.95 | 1.57 | 0.050 |
| σ | C 40 - F 46 | σ* | C 38 - C 39 | 2.26 | 1.54 | 0.053 |
| LP ( 1) | C 36 | π* | C 4 - C 5 | 65.27 | 0.16 | 0.102 |
| LP ( 1) | C 36 | π* | C 34 - C 35 | 74.59 | 0.14 | 0.108 |
| LP ( 1) | C 36 | π* | C 37 - C 38 | 74.59 | 0.14 | 0.108 |
| LP ( 1) | F 41 | σ* | C 17 - C 18 | 1.41 | 1.57 | 0.042 |
| LP ( 1) | F 41 | σ* | C 18 - C 19 | 1.54 | 1.60 | 0.044 |
| LP ( 2) | F 41 | σ* | C 17 - C 18 | 7.19 | 0.96 | 0.074 |
| LP ( 2) | F 41 | σ* | C 18 - C 19 | 6.73 | 0.99 | 0.073 |
| LP ( 3) | F 41 | π* | C 18 - C 19 | 21.03 | 0.43 | 0.092 |
| LP ( 1) | F 42 | σ* | C 19 - C 20 | 1.63 | 1.54 | 0.045 |
| LP ( 1) | F 42 | σ* | C 20 - C 22 | 1.31 | 1.63 | 0.042 |
| LP ( 2) | F 42 | σ* | C 19 - C 20 | 7.44 | 0.93 | 0.074 |
| LP ( 2) | F 42 | σ* | C 20 - C 22 | 6.65 | 1.02 | 0.073 |
| LP ( 2) | F 42 | σ* | C 21 - C 22 | 0.55 | 0.93 | 0.020 |
| LP ( 3) | F 42 | π* | C 20 - C 22 | 21.10 | 0.43 | 0.090 |
| LP ( 1) | F 43 | σ* | C 20 - C 22 | 1.30 | 1.63 | 0.041 |
| LP ( 1) | F 43 | σ* | C 21 - C 22 | 1.66 | 1.54 | 0.045 |
| LP ( 2) | F 43 | σ* | C 19 - C 20 | 0.53 | 0.93 | 0.020 |
| LP ( 2) | F 43 | σ* | C 20 - C 22 | 6.73 | 1.02 | 0.074 |
| LP ( 2) | F 43 | σ* | C 21 - C 22 | 7.24 | 0.93 | 0.073 |
| LP ( 3) | F 43 | π* | C 20 - C 22 | 20.90 | 0.43 | 0.090 |
| LP ( 1) | F 44 | σ* | C 21 - C 25 | 1.78 | 1.58 | 0.048 |
| LP ( 1) | F 44 | σ* | C 24 - C 25 | 1.23 | 1.59 | 0.040 |
| LP ( 2) | F 44 | σ* | C 21 - C 25 | 7.02 | 0.97 | 0.074 |
| LP ( 2) | F 44 | σ* | C 23 - C 24 | 0.54 | 0.97 | 0.020 |
| LP ( 2) | F 44 | σ* | C 24 - C 25 | 6.96 | 0.98 | 0.074 |
| LP ( 3) | F 44 | π* | C 24 - C 25 | 20.11 | 0.42 | 0.089 |
| LP ( 1) | F 45 | σ* | C 23 - C 24 | 1.78 | 1.58 | 0.048 |
| LP ( 1) | F 45 | σ* | C 24 - C 25 | 1.23 | 1.59 | 0.040 |
| LP ( 2) | F 45 | σ* | C 21 - C 25 | 0.54 | 0.97 | 0.020 |
| LP ( 2) | F 45 | σ* | C 23 - C 24 | 7.02 | 0.97 | 0.074 |
| LP ( 2) | F 45 | σ* | C 24 - C 25 | 6.96 | 0.98 | 0.074 |
| LP ( 3) | F 45 | π* | C 24 - C 25 | 20.11 | 0.42 | 0.089 |
| LP ( 1) | F 46 | σ* | C 23 - C 40 | 1.66 | 1.54 | 0.045 |
| LP ( 1) | F 46 | σ* | C 39 - C 40 | 1.30 | 1.63 | 0.041 |
| LP ( 2) | F 46 | σ* | C 23 - C 40 | 7.24 | 0.93 | 0.073 |
| LP ( 2) | F 46 | σ* | C 38 - C 39 | 0.53 | 0.93 | 0.020 |
| LP ( 2) | F 46 | σ* | C 39 - C 40 | 6.73 | 1.02 | 0.074 |
| LP ( 3) | F 46 | π* | C 39 - C 40 | 20.90 | 0.43 | 0.090 |
| LP ( 1) | F 47 | σ* | C 38 - C 39 | 1.63 | 1.54 | 0.045 |
| LP ( 1) | F 47 | σ* | C 39 - C 40 | 1.31 | 1.63 | 0.042 |
| LP ( 2) | F 47 | σ* | C 23 - C 40 | 0.55 | 0.93 | 0.020 |
| LP ( 2) | F 47 | σ* | C 38 - C 39 | 7.44 | 0.93 | 0.074 |
| LP ( 2) | F 47 | σ* | C 39 - C 40 | 6.65 | 1.02 | 0.073 |
| LP ( 3) | F 47 | σ* | C 39 - C 40 | 21.10 | 0.43 | 0.090 |
| LP ( 1) | F 48 | σ* | C 36 - C 37 | 1.41 | 1.57 | 0.042 |
| LP ( 1) | F 48 | σ* | C 37 - C 38 | 1.54 | 1.60 | 0.044 |
| LP ( 2) | F 48 | σ* | C 36 - C 37 | 7.19 | 0.96 | 0.074 |
| LP ( 2) | F 48 | σ* | C 37 - C 38 | 6.73 | 0.99 | 0.073 |
| LP ( 3) | F 48 | π* | C 37 - C 38 | 21.03 | 0.43 | 0.092 |
| LP ( 1) | F 49 | σ* | C 34 - C 35 | 1.54 | 1.60 | 0.044 |
| LP ( 1) | F 49 | σ* | C 35 - C 36 | 1.41 | 1.57 | 0.042 |
| LP ( 2) | F 49 | σ* | C 34 - C 35 | 6.73 | 0.99 | 0.073 |
| LP ( 2) | F 49 | σ* | C 35 - C 36 | 7.19 | 0.96 | 0.074 |
| LP ( 3) | F 49 | π* | C 34 - C 35 | 21.03 | 0.43 | 0.092 |
| LP ( 1) | F 50 | σ* | C 32 - C 33 | 1.31 | 1.63 | 0.042 |
| LP ( 1) | F 50 | σ* | C 33 - C 34 | 1.63 | 1.54 | 0.045 |
| LP ( 2) | F 50 | σ* | C 31 - C 32 | 0.55 | 0.93 | 0.020 |
| LP ( 2) | F 50 | σ* | C 32 - C 33 | 6.65 | 1.02 | 0.073 |
| LP ( 2) | F 50 | σ* | C 33 - C 34 | 7.44 | 0.93 | 0.074 |
| LP ( 3) | F 50 | π* | C 32 - C 33 | 21.10 | 0.43 | 0.090 |
| LP ( 1) | F 51 | σ* | C 31 - C 32 | 1.66 | 1.54 | 0.045 |
| LP ( 1) | F 51 | σ* | C 32 - C 33 | 1.30 | 1.63 | 0.041 |
| LP ( 2) | F 51 | σ* | C 31 - C 32 | 7.24 | 0.93 | 0.073 |
| LP ( 2) | F 51 | σ* | C 32 - C 33 | 6.73 | 1.02 | 0.074 |
| LP ( 2) | F 51 | σ* | C 33 - C 34 | 0.53 | 0.93 | 0.020 |
| LP ( 3) | F 51 | π* | C 32 - C 33 | 20.90 | 0.43 | 0.090 |
| LP ( 1) | F 52 | σ* | C 29 - C 30 | 1.23 | 1.59 | 0.040 |
| LP ( 1) | F 52 | σ* | C 30 - C 31 | 1.78 | 1.58 | 0.048 |
| LP ( 2) | F 52 | σ* | C 28 - C 29 | 0.54 | 0.97 | 0.020 |
| LP ( 2) | F 52 | σ* | C 29 - C 30 | 6.96 | 0.98 | 0.074 |
| LP ( 2) | F 52 | σ* | C 30 - C 31 | 7.02 | 0.97 | 0.074 |
| LP ( 3) | F 52 | π* | C 29 - C 30 | 20.11 | 0.42 | 0.089 |
| LP ( 1) | F 53 | σ* | C 28 - C 29 | 1.78 | 1.58 | 0.048 |
| LP ( 1) | F 53 | σ* | C 29 - C 30 | 1.23 | 1.59 | 0.040 |
| LP ( 2) | F 53 | σ* | C 28 - C 29 | 7.02 | 0.97 | 0.074 |
| LP ( 2) | F 53 | σ* | C 29 - C 30 | 6.96 | 0.98 | 0.074 |
| LP ( 2) | F 53 | σ* | C 30 - C 31 | 0.54 | 0.97 | 0.020 |
| LP ( 3) | F 53 | π* | C 29 - C 30 | 20.11 | 0.42 | 0.089 |
| LP ( 1) | F 54 | σ* | C 16 - C 26 | 1.63 | 1.54 | 0.045 |
| LP ( 1) | F 54 | σ* | C 26 - C 27 | 1.31 | 1.63 | 0.042 |
| LP ( 2) | F 54 | σ* | C 16 - C 26 | 7.44 | 0.93 | 0.074 |
| LP ( 2) | F 54 | σ* | C 26 - C 27 | 6.65 | 1.02 | 0.073 |
| LP ( 2) | F 54 | σ* | C 27 - C 28 | 0.55 | 0.93 | 0.020 |
| LP ( 3) | F 54 | π* | C 26 - C 27 | 21.10 | 0.43 | 0.090 |
| LP ( 1) | F 55 | σ* | C 15 - C 16 | 1.54 | 1.60 | 0.044 |
| LP ( 1) | F 55 | σ* | C 15 - C 17 | 1.41 | 1.57 | 0.042 |
| LP ( 2) | F 55 | σ* | C 15 - C 16 | 6.73 | 0.99 | 0.073 |
| LP ( 2) | F 55 | σ* | C 15 - C 17 | 7.19 | 0.96 | 0.074 |
| LP ( 3) | F 55 | π* | C 15 - C 16 | 21.03 | 0.43 | 0.092 |
| LP ( 1) | F 56 | σ* | C 26 - C 27 | 1.30 | 1.63 | 0.041 |
| LP ( 1) | F 56 | σ* | C 27 - C 28 | 1.66 | 1.54 | 0.045 |
| LP ( 2) | F 56 | σ* | C 16 - C 26 | 0.53 | 0.93 | 0.020 |
| LP ( 2) | F 56 | σ* | C 26 - C 27 | 6.73 | 1.02 | 0.074 |
| LP ( 2) | F 56 | σ* | C 27 - C 28 | 7.24 | 0.93 | 0.073 |
| LP ( 3) | F 56 | π* | C 26 - C 27 | 20.90 | 0.43 | 0.090 |

E(2) : means energy of hyper conjugative interaction (stabilization energy).

E(j)-E(i) : Energy difference between donor and acceptor i and j NBO orbitals.

F (i, j) is the Fock matrix element between i and j NBO orbitals.

Table S7: Second order perturbation theory analysis of Fock matrix in NBO basis of C_40_H_10_F_6_ using B3LYP/cc-pVDZ

| C_40_H_10_F_6_/B3LYP/cc-pVDZ | | |  | E(2) | E(j)-E(i) | F (i, j) |
| --- | --- | --- | --- | --- | --- | --- |
| Donor NBO (i) | Types | Acceptor NBO (j) | Types | kcal/mol | a.u. | a.u. |
| σ | C 1 - C 2 | σ* | C 1 - C 6 | 4.10 | 1.27 | 0.064 |
| σ | C 1 - C 2 | σ* | C 1 - C 17 | 3.69 | 1.23 | 0.060 |
| σ | C 1 - C 2 | σ* | C 2 - C 3 | 3.72 | 1.24 | 0.061 |
| σ | C 1 - C 2 | σ* | C 2 - C 11 | 4.20 | 1.27 | 0.065 |
| σ | C 1 - C 2 | σ* | C 3 - C 14 | 2.94 | 1.27 | 0.055 |
| σ | C 1 - C 2 | σ* | C 6 - C 7 | 3.11 | 1.27 | 0.056 |
| σ | C 1 - C 2 | σ* | C 11 - C 12 | 3.12 | 1.26 | 0.056 |
| σ | C 1 - C 2 | σ* | C 17 - C 18 | 2.69 | 1.24 | 0.052 |
| π | C 1 - C 2 | π* | C 6 - C 7 | 21.75 | 0.28 | 0.071 |
| π | C 1 - C 2 | π* | C 11 - C 12 | 22.47 | 0.28 | 0.072 |
| π | C 1 - C 2 | π* | C 17 - C 18 | 20.87 | 0.27 | 0.067 |
| σ | C 1 - C 6 | σ* | C 1 - C 2 | 4.17 | 1.26 | 0.065 |
| σ | C 1 - C 6 | σ* | C 1 - C 17 | 3.56 | 1.23 | 0.059 |
| σ | C 1 - C 6 | σ* | C 2 - C 11 | 3.14 | 1.27 | 0.056 |
| σ | C 1 - C 6 | σ* | C 5 - C 6 | 3.78 | 1.24 | 0.061 |
| σ | C 1 - C 6 | σ* | C 5 - C 8 | 2.99 | 1.27 | 0.055 |
| σ | C 1 - C 6 | σ* | C 6 - C 7 | 4.11 | 1.27 | 0.065 |
| σ | C 1 - C 6 | σ* | C 7 - C 10 | 3.09 | 1.26 | 0.056 |
| σ | C 1 - C 6 | σ* | C 15 - C 17 | 2.70 | 1.25 | 0.052 |
| σ | C 1 - C 17 | σ* | C 1 - C 2 | 3.67 | 1.26 | 0.061 |
| σ | C 1 - C 17 | σ* | C 1 - C 6 | 3.82 | 1.26 | 0.062 |
| σ | C 1 - C 17 | σ* | C 2 - C 3 | 3.33 | 1.24 | 0.057 |
| σ | C 1 - C 17 | σ* | C 5 - C 6 | 3.27 | 1.24 | 0.057 |
| σ | C 1 - C 17 | σ* | C 15 - C 17 | 3.70 | 1.24 | 0.061 |
| σ | C 1 - C 17 | σ* | C 15 - H 45 | 2.36 | 1.13 | 0.046 |
| σ | C 1 - C 17 | σ* | C 17 - C 18 | 2.96 | 1.23 | 0.054 |
| σ | C 1 - C 17 | σ* | C 18 - F 42 | 3.67 | 0.92 | 0.052 |
| σ | C 2 - C 3 | σ* | C 1 - C 2 | 3.77 | 1.25 | 0.061 |
| σ | C 2 - C 3 | σ* | C 1 - C 17 | 3.17 | 1.22 | 0.056 |
| σ | C 2 - C 3 | σ* | C 2 - C 11 | 3.88 | 1.26 | 0.063 |
| σ | C 2 - C 3 | σ* | C 3 - C 4 | 3.77 | 1.25 | 0.061 |
| σ | C 2 - C 3 | σ* | C 3 - C 14 | 3.88 | 1.26 | 0.063 |
| σ | C 2 - C 3 | σ* | C 4 - C 36 | 3.17 | 1.22 | 0.056 |
| σ | C 2 - C 3 | σ* | C 11 - C 16 | 3.20 | 1.22 | 0.056 |
| σ | C 2 - C 3 | σ* | C 14 - C 34 | 3.20 | 1.22 | 0.056 |
| σ | C 2 - C 11 | σ* | C 1 - C 2 | 4.21 | 1.27 | 0.065 |
| σ | C 2 - C 11 | σ* | C 1 - C 6 | 3.11 | 1.27 | 0.056 |
| σ | C 2 - C 11 | σ* | C 2 - C 3 | 3.86 | 1.25 | 0.062 |
| σ | C 2 - C 11 | σ* | C 3 - C 4 | 2.94 | 1.27 | 0.055 |
| σ | C 2 - C 11 | σ* | C 11 - C 12 | 4.08 | 1.26 | 0.064 |
| σ | C 2 - C 11 | σ* | C 11 - C 16 | 3.69 | 1.23 | 0.060 |
| σ | C 2 - C 11 | σ* | C 12 - C 28 | 3.03 | 1.25 | 0.055 |

Table S7: Continued

| C_40_H_10_F_6_/B3LYP/cc-pVDZ | | |  | E(2) | E(j)-E(i) | F (i, j) |
| --- | --- | --- | --- | --- | --- | --- |
| Donor NBO (i) | Types | Acceptor NBO (j) | Types | kcal/mol | a.u. | a.u. |
| σ | C 3 - C 4 | σ* | C 2 - C 3 | 3.72 | 1.24 | 0.061 |
| σ | C 3 - C 4 | σ* | C 2 - C 11 | 2.94 | 1.27 | 0.055 |
| σ | C 3 - C 4 | σ* | C 3 - C 14 | 4.20 | 1.27 | 0.065 |
| σ | C 3 - C 4 | σ* | C 4 - C 5 | 4.09 | 1.27 | 0.064 |
| σ | C 3 - C 4 | σ* | C 4 - C 36 | 3.69 | 1.23 | 0.060 |
| σ | C 3 - C 4 | σ* | C 5 - C 8 | 3.10 | 1.27 | 0.056 |
| σ | C 3 - C 4 | σ* | C 13 - C 14 | 3.12 | 1.26 | 0.056 |
| σ | C 3 - C 4 | σ* | C 36 - C 37 | 2.69 | 1.24 | 0.052 |
| σ | C 3 - C 14 | σ* | C 1 - C 2 | 2.94 | 1.27 | 0.055 |
| σ | C 3 - C 14 | σ* | C 2 - C 3 | 3.86 | 1.25 | 0.062 |
| σ | C 3 - C 14 | σ* | C 3 - C 4 | 4.20 | 1.27 | 0.065 |
| σ | C 3 - C 14 | σ* | C 4 - C 5 | 3.11 | 1.27 | 0.056 |
| σ | C 3 - C 14 | σ* | C 13 - C 14 | 4.08 | 1.26 | 0.064 |
| σ | C 3 - C 14 | σ* | C 13 - C 31 | 3.03 | 1.25 | 0.055 |
| σ | C 3 - C 14 | σ* | C 14 - C 34 | 3.69 | 1.23 | 0.060 |
| σ | C 3 - C 14 | σ* | C 33 - C 34 | 2.63 | 1.21 | 0.050 |
| σ | C 4 - C 5 | σ* | C 3 - C 4 | 4.16 | 1.26 | 0.065 |
| σ | C 4 - C 5 | σ* | C 3 - C 14 | 3.14 | 1.27 | 0.057 |
| σ | C 4 - C 5 | σ* | C 4 - C 36 | 3.56 | 1.23 | 0.059 |
| σ | C 4 - C 5 | σ* | C 5 - C 6 | 3.78 | 1.24 | 0.061 |
| σ | C 4 - C 5 | σ* | C 5 - C 8 | 4.11 | 1.27 | 0.065 |
| σ | C 4 - C 5 | σ* | C 6 - C 7 | 2.99 | 1.27 | 0.055 |
| σ | C 4 - C 5 | σ* | C 8 - C 9 | 3.09 | 1.26 | 0.056 |
| σ | C 4 - C 5 | σ* | C 35 - C 36 | 2.70 | 1.24 | 0.052 |
| π | C 4 - C 5 | π* | C 6 - C 7 | 20.39 | 0.28 | 0.068 |
| π | C 4 - C 5 | π* | C 8 - C 38 | 22.32 | 0.27 | 0.071 |
| π | C 4 - C 5 | π* | C 36 - C 37 | 18.53 | 0.27 | 0.063 |
| σ | C 4 - C 36 | σ* | C 2 - C 3 | 3.33 | 1.24 | 0.057 |
| σ | C 4 - C 36 | σ* | C 3 - C 4 | 3.67 | 1.26 | 0.061 |
| σ | C 4 - C 36 | σ* | C 4 - C 5 | 3.81 | 1.26 | 0.062 |
| σ | C 4 - C 36 | σ* | C 5 - C 6 | 3.27 | 1.24 | 0.057 |
| σ | C 4 - C 36 | σ* | C 35 - C 36 | 3.69 | 1.24 | 0.061 |
| σ | C 4 - C 36 | σ* | C 35 - H 53 | 2.36 | 1.13 | 0.046 |
| σ | C 4 - C 36 | σ* | C 36 - C 37 | 2.96 | 1.23 | 0.054 |
| σ | C 4 - C 36 | σ* | C 37 - F 51 | 3.68 | 0.92 | 0.052 |
| σ | C 5 - C 6 | σ* | C 1 - C 6 | 3.79 | 1.25 | 0.062 |
| σ | C 5 - C 6 | σ* | C 1 - C 17 | 3.23 | 1.22 | 0.056 |
| σ | C 5 - C 6 | σ* | C 4 - C 5 | 3.78 | 1.25 | 0.062 |
| σ | C 5 - C 6 | σ* | C 4 - C 36 | 3.23 | 1.22 | 0.056 |
| σ | C 5 - C 6 | σ* | C 5 - C 8 | 3.94 | 1.26 | 0.063 |
| σ | C 5 - C 6 | σ* | C 6 - C 7 | 3.94 | 1.26 | 0.063 |
| σ | C 5 - C 6 | σ* | C 7 - C 19 | 3.30 | 1.20 | 0.056 |
| σ | C 5 - C 6 | σ* | C 8 - C 38 | 3.30 | 1.20 | 0.056 |
| σ | C 5 - C 8 | σ* | C 1 - C 6 | 2.86 | 1.27 | 0.054 |
| σ | C 5 - C 8 | σ* | C 3 - C 4 | 3.16 | 1.27 | 0.057 |
| σ | C 5 - C 8 | σ* | C 4 - C 5 | 4.18 | 1.27 | 0.065 |
| σ | C 5 - C 8 | σ* | C 5 - C 6 | 3.88 | 1.25 | 0.062 |
| σ | C 5 - C 8 | σ* | C 8 - C 9 | 4.13 | 1.26 | 0.065 |
| σ | C 5 - C 8 | σ* | C 8 - C 38 | 3.40 | 1.22 | 0.058 |
| σ | C 5 - C 8 | σ* | C 9 - C 23 | 3.09 | 1.25 | 0.056 |
| σ | C 5 - C 8 | σ* | C 38 - C 39 | 2.49 | 1.20 | 0.049 |
| σ | C 6 - C 7 | σ* | C 1 - C 2 | 3.16 | 1.27 | 0.057 |
| σ | C 6 - C 7 | σ* | C 1 - C 6 | 4.18 | 1.27 | 0.065 |
| σ | C 6 - C 7 | σ* | C 4 - C 5 | 2.86 | 1.27 | 0.054 |
| σ | C 6 - C 7 | σ* | C 5 - C 6 | 3.88 | 1.25 | 0.062 |
| σ | C 6 - C 7 | σ* | C 7 - C 10 | 4.13 | 1.26 | 0.065 |
| σ | C 6 - C 7 | σ* | C 7 - C 19 | 3.40 | 1.22 | 0.057 |
| σ | C 6 - C 7 | σ* | C 10 - C 21 | 3.09 | 1.25 | 0.056 |
| σ | C 6 - C 7 | σ* | C 19 - C 20 | 2.49 | 1.20 | 0.049 |
| π | C 6 - C 7 | π* | C 1 - C 2 | 22.07 | 0.29 | 0.071 |
| π | C 6 - C 7 | π* | C 4 - C 5 | 19.88 | 0.29 | 0.068 |
| π | C 6 - C 7 | π* | C 9 - C 10 | 18.55 | 0.29 | 0.066 |
| σ | C 7 - C 10 | σ* | C 1 - C 6 | 3.29 | 1.26 | 0.058 |

Table S7: Continued

| C_40_H_10_F_6_/B3LYP/cc-pVDZ | | |  | E(2) | E(j)-E(i) | F (i, j) |
| --- | --- | --- | --- | --- | --- | --- |
| Donor NBO (i) | Types | Acceptor NBO (j) | Types | kcal/mol | a.u. | a.u. |
| σ | C 7 - C 10 | σ* | C 6 - C 7 | 4.19 | 1.27 | 0.065 |
| σ | C 7 - C 10 | σ* | C 7 - C 19 | 3.29 | 1.21 | 0.056 |
| σ | C 7 - C 10 | σ* | C 9 - C 10 | 3.79 | 1.25 | 0.062 |
| σ | C 7 - C 10 | σ* | C 9 - C 23 | 2.92 | 1.25 | 0.054 |
| σ | C 7 - C 10 | σ* | C 10 - C 21 | 3.81 | 1.25 | 0.062 |
| σ | C 7 - C 10 | σ* | C 18 - C 19 | 2.44 | 1.26 | 0.050 |
| σ | C 7 - C 10 | σ* | C 21 - C 25 | 2.71 | 1.25 | 0.052 |
| σ | C 7 - C 19 | σ* | C 5 - C 6 | 3.34 | 1.23 | 0.058 |
| σ | C 7 - C 19 | σ* | C 6 - C 7 | 3.51 | 1.26 | 0.060 |
| σ | C 7 - C 19 | σ* | C 7 - C 10 | 3.36 | 1.25 | 0.058 |
| σ | C 7 - C 19 | σ* | C 9 - C 10 | 3.10 | 1.24 | 0.056 |
| σ | C 7 - C 19 | σ* | C 18 - C 19 | 3.66 | 1.26 | 0.061 |
| σ | C 7 - C 19 | σ* | C 18 - F 42 | 4.59 | 0.92 | 0.058 |
| σ | C 7 - C 19 | σ* | C 19 - C 20 | 2.76 | 1.19 | 0.051 |
| σ | C 7 - C 19 | σ* | C 20 - F 54 | 3.68 | 0.93 | 0.052 |
| σ | C 8 - C 9 | σ* | C 4 - C 5 | 3.30 | 1.26 | 0.058 |
| σ | C 8 - C 9 | σ* | C 5 - C 8 | 4.19 | 1.27 | 0.065 |
| σ | C 8 - C 9 | σ* | C 8 - C 38 | 3.29 | 1.21 | 0.056 |
| σ | C 8 - C 9 | σ* | C 9 - C 10 | 3.78 | 1.25 | 0.062 |
| σ | C 8 - C 9 | σ* | C 9 - C 23 | 3.81 | 1.25 | 0.062 |
| σ | C 8 - C 9 | σ* | C 10 - C 21 | 2.92 | 1.25 | 0.054 |
| σ | C 8 - C 9 | σ* | C 23 - C 24 | 2.71 | 1.25 | 0.052 |
| σ | C 8 - C 9 | σ* | C 37 - C 38 | 2.44 | 1.26 | 0.050 |
| σ | C 8 - C 38 | σ* | C 5 - C 6 | 3.35 | 1.23 | 0.058 |
| σ | C 8 - C 38 | σ* | C 5 - C 8 | 3.51 | 1.26 | 0.060 |
| σ | C 8 - C 38 | σ* | C 8 - C 9 | 3.36 | 1.25 | 0.058 |
| σ | C 8 - C 38 | σ* | C 9 - C 10 | 3.10 | 1.24 | 0.056 |
| σ | C 8 - C 38 | σ* | C 37 - C 38 | 3.65 | 1.26 | 0.061 |
| σ | C 8 - C 38 | σ* | C 37 - F 51 | 4.59 | 0.92 | 0.058 |
| σ | C 8 - C 38 | σ* | C 38 - C 39 | 2.75 | 1.19 | 0.051 |
| σ | C 8 - C 38 | σ* | C 39 - F 55 | 3.68 | 0.93 | 0.052 |
| π | C 8 - C 38 | π* | C 4 - C 5 | 17.95 | 0.28 | 0.064 |
| π | C 8 - C 38 | π* | C 9 - C 10 | 19.66 | 0.28 | 0.067 |
| π | C 8 - C 38 | π* | C 36 - C 37 | 25.91 | 0.27 | 0.074 |
| π | C 8 - C 38 | π* | C 39 - C 40 | 17.00 | 0.28 | 0.066 |
| σ | C 9 - C 10 | σ* | C 7 - C 10 | 3.88 | 1.25 | 0.062 |
| σ | C 9 - C 10 | σ* | C 7 - C 19 | 3.26 | 1.20 | 0.056 |
| σ | C 9 - C 10 | σ* | C 8 - C 9 | 3.88 | 1.25 | 0.062 |
| σ | C 9 - C 10 | σ* | C 8 - C 38 | 3.26 | 1.20 | 0.056 |
| σ | C 9 - C 10 | σ* | C 9 - C 23 | 3.81 | 1.24 | 0.061 |
| σ | C 9 - C 10 | σ* | C 10 - C 21 | 3.81 | 1.24 | 0.061 |
| σ | C 9 - C 10 | σ* | C 21 - C 22 | 2.83 | 1.21 | 0.052 |
| σ | C 9 - C 10 | σ* | C 23 - C 40 | 2.83 | 1.21 | 0.052 |
| π | C 9 - C 10 | LP ( 1) | C 19 | 0.56 | 0.12 | 0.009 |
| π | C 9 - C 10 | π* | C 6 - C 7 | 19.24 | 0.28 | 0.066 |
| π | C 9 - C 10 | π* | C 8 - C 38 | 23.57 | 0.26 | 0.072 |
| π | C 9 - C 10 | π* | C 21 - C 25 | 19.21 | 0.28 | 0.066 |
| π | C 9 - C 10 | π* | C 23 - C 24 | 19.22 | 0.28 | 0.066 |
| σ | C 9 - C 23 | σ* | C 5 - C 8 | 3.06 | 1.26 | 0.056 |
| σ | C 9 - C 23 | σ* | C 7 - C 10 | 3.19 | 1.25 | 0.056 |
| σ | C 9 - C 23 | σ* | C 8 - C 9 | 3.88 | 1.25 | 0.062 |
| σ | C 9 - C 23 | σ* | C 9 - C 10 | 3.96 | 1.25 | 0.063 |
| σ | C 9 - C 23 | σ* | C 23 - C 24 | 3.54 | 1.25 | 0.060 |
| σ | C 9 - C 23 | σ* | C 23 - C 40 | 2.87 | 1.21 | 0.053 |
| σ | C 9 - C 23 | σ* | C 24 - H 52 | 2.39 | 1.12 | 0.046 |
| σ | C 9 - C 23 | σ* | C 40 - H 47 | 2.24 | 1.12 | 0.045 |
| σ | C 10 - C 21 | σ* | C 6 - C 7 | 3.06 | 1.26 | 0.056 |
| σ | C 10 - C 21 | σ* | C 7 - C 10 | 3.88 | 1.25 | 0.062 |
| σ | C 10 - C 21 | σ* | C 8 - C 9 | 3.19 | 1.25 | 0.056 |
| σ | C 10 - C 21 | σ* | C 9 - C 10 | 3.96 | 1.25 | 0.063 |
| σ | C 10 - C 21 | σ* | C 21 - C 22 | 2.87 | 1.21 | 0.053 |
| σ | C 10 - C 21 | σ* | C 21 - C 25 | 3.54 | 1.25 | 0.059 |
| σ | C 10 - C 21 | σ* | C 22 - H 49 | 2.24 | 1.12 | 0.045 |
| σ | C 10 - C 21 | σ* | C 25 - H 46 | 2.39 | 1.12 | 0.046 |
| σ | C 11 - C 12 | σ* | C 1 - C 2 | 3.27 | 1.26 | 0.057 |
| σ | C 11 - C 12 | σ* | C 2 - C 11 | 4.21 | 1.27 | 0.065 |
| σ | C 11 - C 12 | σ* | C 11 - C 16 | 3.45 | 1.22 | 0.058 |
| σ | C 11 - C 12 | σ* | C 12 - C 13 | 3.88 | 1.26 | 0.062 |
| σ | C 11 - C 12 | σ* | C 12 - C 28 | 3.79 | 1.25 | 0.061 |
| σ | C 11 - C 12 | σ* | C 13 - C 31 | 3.05 | 1.25 | 0.055 |
| σ | C 11 - C 12 | σ* | C 15 - C 16 | 2.60 | 1.27 | 0.052 |
| σ | C 11 - C 12 | σ* | C 28 - C 29 | 2.67 | 1.25 | 0.052 |
| π | C 11 - C 12 | π* | C 1 - C 2 | 22.40 | 0.28 | 0.071 |
| π | C 11 - C 12 | π* | C 13 - C 14 | 19.49 | 0.28 | 0.066 |
| π | C 11 - C 12 | π* | C 15 - C 16 | 18.37 | 0.28 | 0.066 |
| π | C 11 - C 12 | π* | C 28 - C 29 | 21.25 | 0.28 | 0.070 |
| σ | C 11 - C 16 | σ* | C 2 - C 3 | 3.42 | 1.23 | 0.058 |
| σ | C 11 - C 16 | σ* | C 2 - C 11 | 3.67 | 1.26 | 0.061 |
| σ | C 11 - C 16 | σ* | C 11 - C 12 | 3.70 | 1.25 | 0.061 |
| σ | C 11 - C 16 | σ* | C 12 - C 13 | 3.11 | 1.25 | 0.056 |
| σ | C 11 - C 16 | σ* | C 15 - C 16 | 4.05 | 1.27 | 0.064 |
| σ | C 11 - C 16 | σ* | C 15 - H 45 | 2.59 | 1.13 | 0.049 |
| σ | C 11 - C 16 | σ* | C 16 - C 26 | 2.52 | 1.19 | 0.049 |
| σ | C 11 - C 16 | σ* | C 26 - F 41 | 3.54 | 0.92 | 0.051 |
| σ | C 12 - C 13 | σ* | C 11 - C 12 | 3.90 | 1.25 | 0.063 |
| σ | C 12 - C 13 | σ* | C 11 - C 16 | 3.23 | 1.22 | 0.056 |
| σ | C 12 - C 13 | σ* | C 12 - C 28 | 3.88 | 1.24 | 0.062 |
| σ | C 12 - C 13 | σ* | C 13 - C 14 | 3.91 | 1.25 | 0.063 |

Table S7: Continued

| C_40_H_10_F_6_/B3LYP/cc-pVDZ | | |  | E(2) | E(j)-E(i) | F (i, j) |
| --- | --- | --- | --- | --- | --- | --- |
| Donor NBO (i) | Types | Acceptor NBO (j) | Types | kcal/mol | a.u. | a.u. |
| σ | C 12 - C 13 | σ* | C 13 - C 14 | 3.91 | 1.25 | 0.063 |
| σ | C 12 - C 13 | σ* | C 13 - C 31 | 3.88 | 1.24 | 0.062 |
| σ | C 12 - C 13 | σ* | C 14 - C 34 | 3.23 | 1.22 | 0.056 |
| σ | C 12 - C 13 | σ* | C 27 - C 28 | 2.85 | 1.21 | 0.053 |
| σ | C 12 - C 13 | σ* | C 31 - C 32 | 2.85 | 1.21 | 0.053 |
| σ | C 12 - C 28 | σ* | C 2 - C 11 | 3.17 | 1.26 | 0.057 |
| σ | C 12 - C 28 | σ* | C 11 - C 12 | 3.95 | 1.25 | 0.063 |
| σ | C 12 - C 28 | σ* | C 12 - C 13 | 4.00 | 1.25 | 0.063 |
| σ | C 12 - C 28 | σ* | C 13 - C 14 | 3.13 | 1.25 | 0.056 |
| σ | C 12 - C 28 | σ* | C 27 - C 28 | 2.81 | 1.21 | 0.052 |
| σ | C 12 - C 28 | σ* | C 27 - H 44 | 2.21 | 1.12 | 0.045 |
| σ | C 12 - C 28 | σ* | C 28 - C 29 | 3.50 | 1.24 | 0.059 |
| σ | C 12 - C 28 | σ* | C 29 - H 56 | 2.42 | 1.12 | 0.047 |
| σ | C 13 - C 14 | σ* | C 3 - C 4 | 3.27 | 1.26 | 0.057 |
| σ | C 13 - C 14 | σ* | C 3 - C 14 | 4.21 | 1.27 | 0.065 |
| σ | C 13 - C 14 | σ* | C 12 - C 13 | 3.88 | 1.26 | 0.062 |
| σ | C 13 - C 14 | σ* | C 12 - C 28 | 3.05 | 1.25 | 0.055 |
| σ | C 13 - C 14 | σ* | C 13 - C 31 | 3.79 | 1.25 | 0.061 |
| σ | C 13 - C 14 | σ* | C 14 - C 34 | 3.45 | 1.23 | 0.058 |
| σ | C 13 - C 14 | σ* | C 30 - C 31 | 2.67 | 1.25 | 0.052 |
| σ | C 13 - C 14 | σ* | C 34 - C 35 | 2.60 | 1.27 | 0.052 |
| π | C 13 - C 14 | π* | C 11 - C 12 | 19.48 | 0.28 | 0.066 |
| π | C 13 - C 14 | π* | C 30 - C 31 | 21.25 | 0.28 | 0.070 |
| π | C 13 - C 14 | π* | C 34 - C 35 | 18.37 | 0.28 | 0.066 |
| σ | C 13 - C 31 | σ* | C 3 - C 14 | 3.17 | 1.26 | 0.057 |
| σ | C 13 - C 31 | σ* | C 11 - C 12 | 3.13 | 1.25 | 0.056 |
| σ | C 13 - C 31 | σ* | C 12 - C 13 | 4.00 | 1.25 | 0.063 |
| σ | C 13 - C 31 | σ* | C 13 - C 14 | 3.95 | 1.25 | 0.063 |
| σ | C 13 - C 31 | σ* | C 30 - C 31 | 3.50 | 1.24 | 0.059 |
| σ | C 13 - C 31 | σ* | C 30 - H 43 | 2.42 | 1.12 | 0.047 |
| σ | C 13 - C 31 | σ* | C 31 - C 32 | 2.81 | 1.21 | 0.052 |
| σ | C 13 - C 31 | σ* | C 32 - H 48 | 2.21 | 1.12 | 0.045 |
| σ | C 14 - C 34 | σ* | C 2 - C 3 | 3.42 | 1.23 | 0.058 |
| σ | C 14 - C 34 | σ* | C 3 - C 14 | 3.67 | 1.26 | 0.061 |
| σ | C 14 - C 34 | σ* | C 12 - C 13 | 3.12 | 1.25 | 0.056 |
| σ | C 14 - C 34 | σ* | C 13 - C 14 | 3.70 | 1.25 | 0.061 |
| σ | C 14 - C 34 | σ* | C 33 - C 34 | 2.52 | 1.19 | 0.049 |
| σ | C 14 - C 34 | σ* | C 33 - F 50 | 3.54 | 0.92 | 0.051 |
| σ | C 14 - C 34 | σ* | C 34 - C 35 | 4.05 | 1.27 | 0.064 |
| σ | C 14 - C 34 | σ* | C 35 - H 53 | 2.59 | 1.13 | 0.049 |
| σ | C 15 - C 16 | σ* | C 11 - C 12 | 3.03 | 1.28 | 0.056 |
| σ | C 15 - C 16 | σ* | C 11 - C 16 | 4.59 | 1.25 | 0.068 |
| σ | C 15 - C 16 | σ* | C 15 - C 17 | 3.02 | 1.27 | 0.055 |
| σ | C 15 - C 16 | σ* | C 15 - H 45 | 1.34 | 1.16 | 0.035 |
| σ | C 15 - C 16 | σ* | C 16 - C 26 | 3.37 | 1.23 | 0.058 |
| σ | C 15 - C 16 | σ* | C 17 - C 18 | 3.53 | 1.26 | 0.060 |
| σ | C 15 - C 16 | σ* | C 26 - C 27 | 2.11 | 1.34 | 0.047 |
| π | C 15 - C 16 | π* | C 11 - C 12 | 18.39 | 0.30 | 0.068 |
| π | C 15 - C 16 | π* | C 17 - C 18 | 19.81 | 0.28 | 0.068 |
| π | C 15 - C 16 | π* | C 26 - C 27 | 19.45 | 0.29 | 0.069 |
| σ | C 15 - C 17 | σ* | C 1 - C 6 | 3.14 | 1.27 | 0.056 |
| σ | C 15 - C 17 | σ* | C 1 - C 17 | 4.21 | 1.24 | 0.065 |
| σ | C 15 - C 17 | σ* | C 15 - C 16 | 3.05 | 1.29 | 0.056 |
| σ | C 15 - C 17 | σ* | C 15 - H 45 | 1.10 | 1.15 | 0.032 |
| σ | C 15 - C 17 | σ* | C 16 - C 26 | 3.97 | 1.21 | 0.062 |
| σ | C 15 - C 17 | σ* | C 17 - C 18 | 3.49 | 1.25 | 0.059 |
| σ | C 15 - C 17 | σ* | C 18 - C 19 | 2.73 | 1.27 | 0.053 |
| σ | C 15 - H 45 | σ* | C 1 - C 17 | 4.96 | 1.07 | 0.065 |
| σ | C 15 - H 45 | σ* | C 11 - C 16 | 5.39 | 1.06 | 0.068 |
| σ | C 15 - H 45 | σ* | C 15 - C 16 | 1.17 | 1.11 | 0.032 |
| σ | C 15 - H 45 | σ* | C 15 - C 17 | 0.86 | 1.08 | 0.027 |
| σ | C 16 - C 26 | σ* | C 2 - C 11 | 3.23 | 1.28 | 0.057 |
| σ | C 16 - C 26 | σ* | C 11 - C 16 | 2.81 | 1.24 | 0.053 |
| σ | C 16 - C 26 | σ* | C 15 - C 16 | 3.92 | 1.29 | 0.064 |
| σ | C 16 - C 26 | σ* | C 15 - C 17 | 2.42 | 1.26 | 0.049 |
| σ | C 16 - C 26 | σ* | C 26 - C 27 | 3.79 | 1.32 | 0.063 |
| σ | C 16 - C 26 | σ* | C 27 - H 44 | 2.69 | 1.14 | 0.050 |
| σ | C 17 - C 18 | σ* | C 1 - C 2 | 3.12 | 1.29 | 0.057 |
| σ | C 17 - C 18 | σ* | C 1 - C 17 | 3.42 | 1.26 | 0.059 |
| σ | C 17 - C 18 | σ* | C 15 - C 16 | 2.08 | 1.30 | 0.047 |
| σ | C 17 - C 18 | σ* | C 15 - C 17 | 3.75 | 1.27 | 0.062 |
| σ | C 17 - C 18 | σ* | C 18 - C 19 | 4.08 | 1.29 | 0.065 |
| σ | C 17 - C 18 | σ* | C 19 - C 20 | 3.65 | 1.22 | 0.060 |
| π | C 17 - C 18 | LP ( 1) | C 19 | 55.07 | 0.14 | 0.096 |
| π | C 17 - C 18 | π* | C 1 - C 2 | 19.66 | 0.30 | 0.069 |
| π | C 17 - C 18 | π* | C 15 - C 16 | 20.75 | 0.30 | 0.072 |
| π | C 17 - C 18 | π* | C 17 - C 18 | 1.11 | 0.28 | 0.016 |
| σ | C 18 - C 19 | σ* | C 7 - C 10 | 2.94 | 1.30 | 0.055 |
| σ | C 18 - C 19 | σ* | C 7 - C 19 | 4.07 | 1.26 | 0.064 |
| σ | C 18 - C 19 | σ* | C 15 - C 17 | 3.01 | 1.29 | 0.056 |
| σ | C 18 - C 19 | σ* | C 17 - C 18 | 3.85 | 1.28 | 0.063 |
| σ | C 18 - C 19 | σ* | C 19 - C 20 | 3.63 | 1.24 | 0.060 |
| σ | C 18 - C 19 | σ* | C 20 - C 22 | 1.87 | 1.36 | 0.045 |
| σ | C 18 - F 42 | σ* | C 1 - C 17 | 2.26 | 1.58 | 0.054 |
| σ | C 18 - F 42 | σ* | C 7 - C 19 | 2.22 | 1.56 | 0.053 |
| σ | C 19 - C 20 | σ* | C 6 - C 7 | 3.14 | 1.28 | 0.057 |
| σ | C 19 - C 20 | σ* | C 7 - C 19 | 3.10 | 1.23 | 0.055 |
| σ | C 19 - C 20 | σ* | C 17 - C 18 | 2.77 | 1.25 | 0.053 |
| σ | C 19 - C 20 | σ* | C 18 - C 19 | 3.84 | 1.28 | 0.063 |
| σ | C 19 - C 20 | σ* | C 20 - C 22 | 3.56 | 1.33 | 0.061 |
| σ | C 19 - C 20 | σ* | C 22 - H 49 | 2.63 | 1.14 | 0.049 |
| σ | C 20 - C 22 | σ* | C 18 - C 19 | 3.00 | 1.32 | 0.056 |
| σ | C 20 - C 22 | σ* | C 19 - C 20 | 3.98 | 1.25 | 0.063 |
| σ | C 20 - C 22 | σ* | C 21 - C 22 | 2.95 | 1.28 | 0.055 |
| σ | C 20 - C 22 | σ* | C 21 - C 25 | 3.19 | 1.31 | 0.058 |
| σ | C 20 - C 22 | σ* | C 22 - H 49 | 1.60 | 1.19 | 0.039 |
| π | C 20 - C 22 | LP ( 1) | C 19 | 31.12 | 0.16 | 0.083 |
| π | C 20 - C 22 | π* | C 20 - C 22 | 0.56 | 0.32 | 0.012 |
| π | C 20 - C 22 | π* | C 21 - C 25 | 15.97 | 0.32 | 0.067 |
| σ | C 20 - F 54 | σ* | C 7 - C 19 | 1.94 | 1.55 | 0.049 |
| σ | C 20 - F 54 | σ* | C 21 - C 22 | 2.10 | 1.56 | 0.051 |
| σ | C 21 - C 22 | σ* | C 9 - C 10 | 3.42 | 1.25 | 0.058 |
| σ | C 21 - C 22 | σ* | C 10 - C 21 | 3.36 | 1.24 | 0.058 |
| σ | C 21 - C 22 | σ* | C 20 - C 22 | 2.67 | 1.31 | 0.053 |
| σ | C 21 - C 22 | σ* | C 20 - F 54 | 4.65 | 0.93 | 0.059 |
| σ | C 21 - C 22 | σ* | C 21 - C 25 | 3.19 | 1.25 | 0.056 |
| σ | C 21 - C 22 | σ* | C 22 - H 49 | 1.08 | 1.12 | 0.031 |
| σ | C 21 - C 22 | σ* | C 24 - C 25 | 2.19 | 1.28 | 0.048 |
| σ | C 21 - C 25 | σ* | C 7 - C 10 | 3.39 | 1.26 | 0.058 |
| σ | C 21 - C 25 | σ* | C 10 - C 21 | 4.01 | 1.26 | 0.063 |
| σ | C 21 - C 25 | σ* | C 20 - C 22 | 1.85 | 1.32 | 0.044 |
| σ | C 21 - C 25 | σ* | C 21 - C 22 | 3.36 | 1.23 | 0.057 |
| σ | C 21 - C 25 | σ* | C 24 - C 25 | 2.83 | 1.29 | 0.054 |
| σ | C 21 - C 25 | σ* | C 24 - H 52 | 2.67 | 1.14 | 0.049 |
| σ | C 21 - C 25 | σ* | C 25 - H 46 | 1.02 | 1.14 | 0.030 |
| π | C 21 - C 25 | π* | C 9 - C 10 | 18.76 | 0.28 | 0.066 |
| π | C 21 - C 25 | π* | C 20 - C 22 | 18.18 | 0.28 | 0.067 |
| π | C 21 - C 25 | π* | C 23 - C 24 | 22.03 | 0.28 | 0.070 |
| σ | C 22 - H 49 | σ* | C 10 - C 21 | 4.42 | 1.09 | 0.062 |
| σ | C 22 - H 49 | σ* | C 19 - C 20 | 5.58 | 1.03 | 0.068 |
| σ | C 22 - H 49 | σ* | C 20 - C 22 | 1.04 | 1.15 | 0.031 |
| σ | C 22 - H 49 | σ* | C 20 - F 54 | 1.54 | 0.78 | 0.031 |
| σ | C 22 - H 49 | σ* | C 21 - C 22 | 0.69 | 1.06 | 0.024 |
| σ | C 23 - C 24 | σ* | C 8 - C 9 | 3.39 | 1.26 | 0.058 |
| σ | C 23 - C 24 | σ* | C 9 - C 23 | 4.01 | 1.26 | 0.063 |
| σ | C 23 - C 24 | σ* | C 23 - C 40 | 3.36 | 1.23 | 0.057 |
| σ | C 23 - C 24 | σ* | C 24 - C 25 | 2.83 | 1.29 | 0.054 |
| σ | C 23 - C 24 | σ* | C 24 - H 52 | 1.02 | 1.14 | 0.030 |
| σ | C 23 - C 24 | σ* | C 25 - H 46 | 2.67 | 1.14 | 0.049 |
| σ | C 23 - C 24 | σ* | C 39 - C 40 | 1.85 | 1.32 | 0.044 |
| π | C 23 - C 24 | π* | C 9 - C 10 | 18.77 | 0.28 | 0.066 |
| π | C 23 - C 24 | π* | C 21 - C 25 | 22.03 | 0.28 | 0.070 |
| π | C 23 - C 24 | π* | C 39 - C 40 | 18.18 | 0.28 | 0.067 |
| σ | C 23 - C 40 | σ* | C 9 - C 10 | 3.42 | 1.25 | 0.058 |
| σ | C 23 - C 40 | σ* | C 9 - C 23 | 3.36 | 1.24 | 0.058 |
| σ | C 23 - C 40 | σ* | C 23 - C 24 | 3.19 | 1.25 | 0.056 |
| σ | C 23 - C 40 | σ* | C 24 - C 25 | 2.19 | 1.28 | 0.048 |
| σ | C 23 - C 40 | σ* | C 39 - C 40 | 2.67 | 1.31 | 0.053 |
| σ | C 23 - C 40 | σ* | C 39 - F 55 | 4.65 | 0.93 | 0.059 |
| σ | C 23 - C 40 | σ* | C 40 - H 47 | 1.08 | 1.12 | 0.031 |
| σ | C 24 - C 25 | σ* | C 21 - C 22 | 3.72 | 1.24 | 0.061 |
| σ | C 24 - C 25 | σ* | C 21 - C 25 | 3.14 | 1.27 | 0.056 |
| σ | C 24 - C 25 | σ* | C 23 - C 24 | 3.14 | 1.27 | 0.056 |
| σ | C 24 - C 25 | σ* | C 23 - C 40 | 3.72 | 1.24 | 0.061 |
| σ | C 24 - C 25 | σ* | C 24 - H 52 | 1.30 | 1.15 | 0.035 |
| σ | C 24 - C 25 | σ* | C 25 - H 46 | 1.30 | 1.15 | 0.035 |
| σ | C 24 - H 52 | σ* | C 9 - C 23 | 4.96 | 1.08 | 0.066 |
| σ | C 24 - H 52 | σ* | C 21 - C 25 | 4.40 | 1.09 | 0.062 |
| σ | C 24 - H 52 | σ* | C 23 - C 24 | 0.70 | 1.09 | 0.025 |
| σ | C 24 - H 52 | σ* | C 24 - C 25 | 0.82 | 1.12 | 0.027 |
| σ | C 25 - H 46 | σ* | C 10 - C 21 | 4.96 | 1.08 | 0.066 |
| σ | C 25 - H 46 | σ* | C 21 - C 25 | 0.70 | 1.09 | 0.025 |
| σ | C 25 - H 46 | σ* | C 23 - C 24 | 4.40 | 1.09 | 0.062 |
| σ | C 25 - H 46 | σ* | C 24 - C 25 | 0.82 | 1.12 | 0.027 |
| σ | C 26 - C 27 | σ* | C 15 - C 16 | 2.90 | 1.34 | 0.056 |
| σ | C 26 - C 27 | σ* | C 16 - C 26 | 4.01 | 1.26 | 0.064 |
| σ | C 26 - C 27 | σ* | C 27 - C 28 | 2.85 | 1.27 | 0.054 |
| σ | C 26 - C 27 | σ* | C 27 - H 44 | 1.64 | 1.18 | 0.039 |
| σ | C 26 - C 27 | σ* | C 28 - C 29 | 3.22 | 1.31 | 0.058 |
| π | C 26 - C 27 | π* | C 15 - C 16 | 15.68 | 0.32 | 0.065 |
| π | C 26 - C 27 | π* | C 28 - C 29 | 15.90 | 0.31 | 0.067 |
| σ | C 26 - F 41 | σ* | C 11 - C 16 | 2.03 | 1.57 | 0.051 |
| σ | C 26 - F 41 | σ* | C 27 - C 28 | 2.00 | 1.56 | 0.050 |
| σ | C 27 - C 28 | σ* | C 12 - C 13 | 3.40 | 1.25 | 0.058 |
| σ | C 27 - C 28 | σ* | C 12 - C 28 | 3.30 | 1.24 | 0.057 |
| σ | C 27 - C 28 | σ* | C 26 - C 27 | 2.68 | 1.30 | 0.053 |
| σ | C 27 - C 28 | σ* | C 26 - F 41 | 5.05 | 0.92 | 0.061 |
| σ | C 27 - C 28 | σ* | C 27 - H 44 | 1.04 | 1.12 | 0.031 |
| σ | C 27 - C 28 | σ* | C 28 - C 29 | 3.14 | 1.24 | 0.056 |
| σ | C 27 - C 28 | σ* | C 29 - C 30 | 2.18 | 1.28 | 0.047 |
| σ | C 27 - H 44 | σ* | C 12 - C 28 | 4.34 | 1.09 | 0.062 |
| σ | C 27 - H 44 | σ* | C 16 - C 26 | 5.42 | 1.04 | 0.067 |
| σ | C 27 - H 44 | σ* | C 26 - C 27 | 1.04 | 1.15 | 0.031 |
| σ | C 27 - H 44 | σ* | C 26 - F 41 | 1.38 | 0.77 | 0.029 |
| σ | C 27 - H 44 | σ* | C 27 - C 28 | 0.67 | 1.05 | 0.024 |
| σ | C 28 - C 29 | σ* | C 11 - C 12 | 3.44 | 1.27 | 0.059 |
| σ | C 28 - C 29 | σ* | C 12 - C 28 | 3.98 | 1.26 | 0.063 |
| σ | C 28 - C 29 | σ* | C 26 - C 27 | 1.77 | 1.32 | 0.043 |
| σ | C 28 - C 29 | σ* | C 27 - C 28 | 3.29 | 1.22 | 0.057 |
| σ | C 28 - C 29 | σ* | C 29 - C 30 | 2.79 | 1.29 | 0.054 |
| σ | C 28 - C 29 | σ* | C 29 - H 56 | 1.02 | 1.14 | 0.030 |
| σ | C 28 - C 29 | σ* | C 30 - H 43 | 2.61 | 1.14 | 0.049 |
| π | C 28 - C 29 | π* | C 11 - C 12 | 20.43 | 0.28 | 0.069 |
| π | C 28 - C 29 | π* | C 26 - C 27 | 17.78 | 0.28 | 0.066 |
| π | C 28 - C 29 | π* | C 30 - C 31 | 21.95 | 0.28 | 0.070 |
| σ | C 29 - C 30 | σ* | C 27 - C 28 | 3.75 | 1.24 | 0.061 |
| σ | C 29 - C 30 | σ* | C 28 - C 29 | 3.13 | 1.27 | 0.056 |
| σ | C 29 - C 30 | σ* | C 29 - H 56 | 1.27 | 1.15 | 0.034 |
| σ | C 29 - C 30 | σ* | C 30 - C 31 | 3.13 | 1.27 | 0.056 |
| σ | C 29 - C 30 | σ* | C 30 - H 43 | 1.27 | 1.15 | 0.034 |
| σ | C 29 - C 30 | σ* | C 31 - C 32 | 3.75 | 1.24 | 0.061 |
| σ | C 29 - H 56 | σ* | C 12 - C 28 | 4.89 | 1.09 | 0.065 |
| σ | C 29 - H 56 | σ* | C 28 - C 29 | 0.68 | 1.09 | 0.024 |
| σ | C 29 - H 56 | σ* | C 29 - C 30 | 0.80 | 1.12 | 0.027 |
| σ | C 29 - H 56 | σ* | C 30 - C 31 | 4.42 | 1.09 | 0.062 |
| σ | C 30 - C 31 | σ* | C 13 - C 14 | 3.44 | 1.27 | 0.059 |
| σ | C 30 - C 31 | σ* | C 13 - C 31 | 3.98 | 1.26 | 0.063 |
| σ | C 30 - C 31 | σ* | C 29 - C 30 | 2.80 | 1.29 | 0.054 |
| σ | C 30 - C 31 | σ* | C 29 - H 56 | 2.61 | 1.14 | 0.049 |

Table S7: Continued

| C_40_H_10_F_6_/B3LYP/cc-pVDZ | | |  | E(2) | E(j)-E(i) | F (i, j) |
| --- | --- | --- | --- | --- | --- | --- |
| Donor NBO (i) | Types | Acceptor NBO (j) | Types | kcal/mol | a.u. | a.u. |
| σ | C 30 - C 31 | σ* | C 29 - H 56 | 2.61 | 1.14 | 0.049 |
| σ | C 30 - C 31 | σ* | C 30 - H 43 | 1.02 | 1.14 | 0.030 |
| σ | C 30 - C 31 | σ* | C 31 - C 32 | 3.29 | 1.22 | 0.057 |
| σ | C 30 - C 31 | σ* | C 32 - C 33 | 1.77 | 1.32 | 0.043 |
| π | C 30 - C 31 | π* | C 13 - C 14 | 20.42 | 0.28 | 0.069 |
| π | C 30 - C 31 | π* | C 28 - C 29 | 21.95 | 0.28 | 0.070 |
| π | C 30 - C 31 | π* | C 32 - C 33 | 17.77 | 0.28 | 0.066 |
| σ | C 30 - H 43 | σ* | C 13 - C 31 | 4.89 | 1.09 | 0.065 |
| σ | C 30 - H 43 | σ* | C 28 - C 29 | 4.42 | 1.09 | 0.062 |
| σ | C 30 - H 43 | σ* | C 29 - C 30 | 0.80 | 1.12 | 0.027 |
| σ | C 30 - H 43 | σ* | C 30 - C 31 | 0.68 | 1.09 | 0.024 |
| σ | C 31 - C 32 | σ* | C 12 - C 13 | 3.40 | 1.25 | 0.058 |
| σ | C 31 - C 32 | σ* | C 13 - C 31 | 3.30 | 1.24 | 0.057 |
| σ | C 31 - C 32 | σ* | C 29 - C 30 | 2.18 | 1.28 | 0.047 |
| σ | C 31 - C 32 | σ* | C 30 - C 31 | 3.13 | 1.24 | 0.056 |
| σ | C 31 - C 32 | σ* | C 32 - C 33 | 2.68 | 1.30 | 0.053 |
| σ | C 31 - C 32 | σ* | C 32 - H 48 | 1.04 | 1.12 | 0.031 |
| σ | C 31 - C 32 | σ* | C 33 - F 50 | 5.05 | 0.92 | 0.061 |
| σ | C 32 - C 33 | σ* | C 30 - C 31 | 3.22 | 1.31 | 0.058 |
| σ | C 32 - C 33 | σ* | C 31 - C 32 | 2.85 | 1.27 | 0.054 |
| σ | C 32 - C 33 | σ* | C 32 - H 48 | 1.64 | 1.19 | 0.039 |
| σ | C 32 - C 33 | σ* | C 33 - C 34 | 4.01 | 1.26 | 0.064 |
| σ | C 32 - C 33 | σ* | C 34 - C 35 | 2.90 | 1.34 | 0.056 |
| π | C 32 - C 33 | π* | C 30 - C 31 | 15.89 | 0.31 | 0.067 |
| π | C 32 - C 33 | π* | C 34 - C 35 | 15.67 | 0.32 | 0.065 |
| σ | C 32 - H 48 | σ* | C 13 - C 31 | 4.34 | 1.09 | 0.062 |
| σ | C 32 - H 48 | σ* | C 31 - C 32 | 0.67 | 1.05 | 0.024 |
| σ | C 32 - H 48 | σ* | C 32 - C 33 | 1.03 | 1.15 | 0.031 |
| σ | C 32 - H 48 | σ* | C 33 - C 34 | 5.42 | 1.04 | 0.067 |
| σ | C 32 - H 48 | σ* | C 33 - F 50 | 1.38 | 0.77 | 0.029 |
| σ | C 33 - C 34 | σ* | C 3 - C 14 | 3.23 | 1.28 | 0.057 |
| σ | C 33 - C 34 | σ* | C 14 - C 34 | 2.81 | 1.24 | 0.053 |
| σ | C 33 - C 34 | σ* | C 32 - C 33 | 3.79 | 1.32 | 0.063 |
| σ | C 33 - C 34 | σ* | C 32 - H 48 | 2.69 | 1.14 | 0.050 |
| σ | C 33 - C 34 | σ* | C 34 - C 35 | 3.92 | 1.29 | 0.064 |
| σ | C 33 - C 34 | σ* | C 35 - C 36 | 2.43 | 1.26 | 0.049 |
| σ | C 33 - F 50 | σ* | C 14 - C 34 | 2.03 | 1.57 | 0.051 |
| σ | C 33 - F 50 | σ* | C 31 - C 32 | 2.00 | 1.56 | 0.050 |
| σ | C 34 - C 35 | σ* | C 13 - C 14 | 3.03 | 1.28 | 0.056 |
| σ | C 34 - C 35 | σ* | C 14 - C 34 | 4.59 | 1.25 | 0.068 |
| σ | C 34 - C 35 | σ* | C 32 - C 33 | 2.11 | 1.34 | 0.047 |
| σ | C 34 - C 35 | σ* | C 33 - C 34 | 3.37 | 1.23 | 0.058 |
| σ | C 34 - C 35 | σ* | C 35 - C 36 | 3.03 | 1.27 | 0.055 |
| σ | C 34 - C 35 | σ* | C 35 - H 53 | 1.34 | 1.16 | 0.035 |
| σ | C 34 - C 35 | σ* | C 36 - C 37 | 3.53 | 1.26 | 0.060 |
| π | C 34 - C 35 | π* | C 13 - C 14 | 18.39 | 0.30 | 0.068 |
| π | C 34 - C 35 | π* | C 32 - C 33 | 19.44 | 0.29 | 0.069 |
| π | C 34 - C 35 | π* | C 36 - C 37 | 19.80 | 0.28 | 0.068 |
| σ | C 35 - C 36 | σ* | C 4 - C 5 | 3.13 | 1.27 | 0.056 |
| σ | C 35 - C 36 | σ* | C 4 - C 36 | 4.21 | 1.24 | 0.065 |
| σ | C 35 - C 36 | σ* | C 33 - C 34 | 3.97 | 1.21 | 0.062 |
| σ | C 35 - C 36 | σ* | C 34 - C 35 | 3.05 | 1.29 | 0.056 |
| σ | C 35 - C 36 | σ* | C 35 - H 53 | 1.11 | 1.15 | 0.032 |
| σ | C 35 - C 36 | σ* | C 36 - C 37 | 3.49 | 1.25 | 0.059 |
| σ | C 35 - C 36 | σ* | C 37 - C 38 | 2.73 | 1.27 | 0.053 |
| σ | C 35 - H 53 | σ* | C 4 - C 36 | 4.95 | 1.07 | 0.065 |
| σ | C 35 - H 53 | σ* | C 14 - C 34 | 5.40 | 1.06 | 0.068 |
| σ | C 35 - H 53 | σ* | C 34 - C 35 | 1.17 | 1.11 | 0.032 |
| σ | C 35 - H 53 | σ* | C 35 - C 36 | 0.86 | 1.08 | 0.027 |
| σ | C 36 - C 37 | σ* | C 3 - C 4 | 3.12 | 1.29 | 0.057 |
| σ | C 36 - C 37 | σ* | C 4 - C 36 | 3.42 | 1.26 | 0.059 |
| σ | C 36 - C 37 | σ* | C 34 - C 35 | 2.08 | 1.31 | 0.047 |
| σ | C 36 - C 37 | σ* | C 35 - C 36 | 3.75 | 1.27 | 0.062 |
| σ | C 36 - C 37 | σ* | C 37 - C 38 | 4.08 | 1.29 | 0.065 |
| σ | C 36 - C 37 | σ* | C 38 - C 39 | 3.65 | 1.22 | 0.060 |
| π | C 36 - C 37 | π* | C 4 - C 5 | 18.42 | 0.30 | 0.067 |
| π | C 36 - C 37 | π* | C 8 - C 38 | 19.29 | 0.28 | 0.068 |
| π | C 36 - C 37 | π* | C 34 - C 35 | 20.74 | 0.30 | 0.072 |
| π | C 36 - C 37 | π* | C 36 - C 37 | 1.11 | 0.28 | 0.016 |
| σ | C 37 - C 38 | σ* | C 8 - C 9 | 2.94 | 1.30 | 0.055 |
| σ | C 37 - C 38 | σ* | C 8 - C 38 | 4.07 | 1.26 | 0.064 |
| σ | C 37 - C 38 | σ* | C 35 - C 36 | 3.01 | 1.29 | 0.056 |
| σ | C 37 - C 38 | σ* | C 36 - C 37 | 3.85 | 1.28 | 0.063 |
| σ | C 37 - C 38 | σ* | C 38 - C 39 | 3.63 | 1.24 | 0.060 |
| σ | C 37 - C 38 | σ* | C 39 - C 40 | 1.88 | 1.36 | 0.045 |
| σ | C 37 - F 51 | σ* | C 4 - C 36 | 2.26 | 1.58 | 0.054 |
| σ | C 37 - F 51 | σ* | C 8 - C 38 | 2.22 | 1.56 | 0.053 |
| σ | C 38 - C 39 | σ* | C 5 - C 8 | 3.14 | 1.28 | 0.057 |
| σ | C 38 - C 39 | σ* | C 8 - C 38 | 3.10 | 1.23 | 0.055 |
| σ | C 38 - C 39 | σ* | C 36 - C 37 | 2.77 | 1.25 | 0.053 |
| σ | C 38 - C 39 | σ* | C 37 - C 38 | 3.84 | 1.28 | 0.063 |
| σ | C 38 - C 39 | σ* | C 39 - C 40 | 3.57 | 1.33 | 0.061 |
| σ | C 38 - C 39 | σ* | C 40 - H 47 | 2.63 | 1.14 | 0.049 |
| σ | C 39 - C 40 | σ* | C 23 - C 24 | 3.19 | 1.31 | 0.058 |
| σ | C 39 - C 40 | σ* | C 23 - C 40 | 2.95 | 1.28 | 0.055 |
| σ | C 39 - C 40 | σ* | C 37 - C 38 | 3.00 | 1.32 | 0.056 |
| σ | C 39 - C 40 | σ* | C 38 - C 39 | 3.99 | 1.25 | 0.063 |
| σ | C 39 - C 40 | σ* | C 40 - H 47 | 1.60 | 1.19 | 0.039 |
| π | C 39 - C 40 | π* | C 8 - C 38 | 12.06 | 0.30 | 0.059 |
| π | C 39 - C 40 | π* | C 23 - C 24 | 15.98 | 0.32 | 0.067 |
| π | C 39 - C 40 | π* | C 39 - C 40 | 0.56 | 0.32 | 0.012 |
| σ | C 39 - F 55 | σ* | C 8 - C 38 | 1.94 | 1.55 | 0.049 |
| σ | C 39 - F 55 | σ* | C 23 - C 40 | 2.10 | 1.56 | 0.051 |
| σ | C 40 - H 47 | σ* | C 9 - C 23 | 4.42 | 1.09 | 0.062 |
| σ | C 40 - H 47 | σ* | C 23 - C 40 | 0.69 | 1.06 | 0.024 |
| σ | C 40 - H 47 | σ* | C 38 - C 39 | 5.58 | 1.03 | 0.068 |
| σ | C 40 - H 47 | σ* | C 39 - C 40 | 1.04 | 1.15 | 0.031 |
| σ | C 40 - H 47 | σ* | C 39 - F 55 | 1.54 | 0.78 | 0.031 |
| LP ( 1) | C 19 | π* | C 6 - C 7 | 61.77 | 0.16 | 0.101 |
| LP ( 1) | C 19 | π* | C 17 - C 18 | 88.28 | 0.14 | 0.113 |
| LP ( 1) | C 19 | π* | C 20 - C 22 | 53.21 | 0.16 | 0.100 |
| LP ( 1) | F 41 | σ* | C 16 - C 26 | 1.46 | 1.55 | 0.043 |
| LP ( 1) | F 41 | σ* | C 26 - C 27 | 1.42 | 1.65 | 0.043 |
| LP ( 2) | F 41 | σ* | C 16 - C 26 | 6.52 | 0.94 | 0.070 |
| LP ( 2) | F 41 | σ* | C 26 - C 27 | 5.97 | 1.04 | 0.071 |
| LP ( 2) | F 41 | σ* | C 27 - C 28 | 0.52 | 0.95 | 0.020 |
| LP ( 3) | F 41 | π* | C 26 - C 27 | 20.46 | 0.45 | 0.089 |
| LP ( 1) | F 42 | σ* | C 17 - C 18 | 1.72 | 1.58 | 0.047 |
| LP ( 1) | F 42 | σ* | C 18 - C 19 | 1.19 | 1.61 | 0.039 |
| LP ( 2) | F 42 | σ* | C 1 - C 17 | 0.53 | 0.97 | 0.020 |
| LP ( 2) | F 42 | σ* | C 17 - C 18 | 5.76 | 0.98 | 0.067 |
| LP ( 2) | F 42 | σ* | C 18 - C 19 | 7.17 | 1.00 | 0.076 |
| LP ( 3) | F 42 | π* | C 17 - C 18 | 18.42 | 0.43 | 0.088 |
| LP ( 1) | F 50 | σ* | C 32 - C 33 | 1.42 | 1.65 | 0.043 |
| LP ( 1) | F 50 | σ* | C 33 - C 34 | 1.46 | 1.55 | 0.043 |
| LP ( 2) | F 50 | σ* | C 31 - C 32 | 0.52 | 0.95 | 0.020 |
| LP ( 2) | F 50 | σ* | C 32 - C 33 | 5.97 | 1.04 | 0.071 |
| LP ( 2) | F 50 | σ* | C 33 - C 34 | 6.52 | 0.94 | 0.070 |
| LP ( 3) | F 50 | π* | C 32 - C 33 | 20.46 | 0.45 | 0.089 |
| LP ( 1) | F 51 | σ* | C 36 - C 37 | 1.72 | 1.58 | 0.047 |
| LP ( 1) | F 51 | σ* | C 37 - C 38 | 1.19 | 1.61 | 0.039 |
| LP ( 2) | F 51 | σ* | C 4 - C 36 | 0.53 | 0.97 | 0.020 |
| LP ( 2) | F 51 | σ* | C 36 - C 37 | 5.76 | 0.98 | 0.067 |
| LP ( 2) | F 51 | σ* | C 37 - C 38 | 7.17 | 1.00 | 0.076 |
| LP ( 3) | F 51 | π* | C 36 - C 37 | 18.42 | 0.43 | 0.088 |
| LP ( 1) | F 54 | σ* | C 19 - C 20 | 1.13 | 1.53 | 0.037 |
| LP ( 1) | F 54 | σ* | C 20 - C 22 | 1.76 | 1.65 | 0.048 |
| LP ( 2) | F 54 | σ* | C 19 - C 20 | 7.77 | 0.92 | 0.076 |
| LP ( 2) | F 54 | σ* | C 20 - C 22 | 5.37 | 1.04 | 0.067 |
| LP ( 2) | F 54 | σ* | C 21 - C 22 | 0.58 | 0.95 | 0.021 |
| LP ( 3) | F 54 | π* | C 20 - C 22 | 20.93 | 0.44 | 0.090 |
| LP ( 1) | F 55 | σ* | C 38 - C 39 | 1.13 | 1.53 | 0.037 |
| LP ( 1) | F 55 | σ* | C 39 - C 40 | 1.76 | 1.65 | 0.048 |
| LP ( 2) | F 55 | σ* | C 23 - C 40 | 0.58 | 0.95 | 0.021 |
| LP ( 2) | F 55 | σ* | C 38 - C 39 | 7.77 | 0.93 | 0.076 |
| LP ( 2) | F 55 | σ* | C 39 - C 40 | 5.37 | 1.04 | 0.067 |
| LP ( 3) | F 55 | π* | C 39 - C 40 | 20.94 | 0.44 | 0.090 |

E(2) : means energy of hyper conjugative interaction (stabilization energy).

E(j)-E(i) : Energy difference between donor and acceptor i and j NBO orbitals.

F (i, j) is the Fock matrix element between i and j NBO orbitals.
